# Supplementary material for: Is the effectiveness of policy-driven mitigation measures on carabid populations driven by landscape and farmland heterogeneity? Applying a modelling approach in the Dutch agroecosystems
Source: PLoS One. 2022 Dec 27;17(12):e0279639. doi: 10.1371/journal.pone.0279639 (PMC9794068; doi:10.1371/journal.pone.0279639)
Supplement: S1 Appendix — (DOCX) [file pone.0279639.s001.docx]

# Appendix A Parametrization of ALMaSS landscape model for the Dutch landscapes

The approach used broadly follows the one presented by Topping et al. (2016), but required specific Dutch conditions and datasets to be taken into account. We described in details, first, the input data used (section 1), and second, the process of generating a complete simulation landscape for ALMaSS divided into two main tasks: generation of landscape map as input for ALMaSS (section 2), and farm classification (section 3).

1. **Input data**
   1. **Land cover / land use information**

For generation of simulation landscapes we used vector map layers from the digital object-oriented topographic database of the Netherlands (TOP10NL) with level of details corresponding with topographic maps at the scale of 1:10 000 (Figure A1). At present, it is the most precise information level of topographic object databases available in the Netherlands (available for browsing at the PDOK viewer https://www.pdok.nl/viewer/, also as WMS service).

We used nine classes of objects from TOP10NL to map 45 different layers of spatial information (Table A1). Individual layers of land use / land cover information together with information on agricultural fields derived from the Agricultural Area Netherlands (Agrarisch Areaal Nederland) were then combined into a single raster landscape map in a step-by-step process (see section 2).

- 1. **The Land Parcel Identification System (LPIS)**

LPIS records information on all agriculturally managed reference parcels (geographically delimited areas with unique identification codes) in the EU Member States, and serves as a controlling mechanism under the CAP. In the Netherlands, LPIS is managed by the Netherlands Enterprise Agency (Rijksdienst voor Ondernemend Nederland). Information on type of crops cultivated in reference parcels, ID numbers of agricultural holdings enabling the grouping of individual reference parcels into farm units, as well as farm types were obtained for 2015 from the agricultural register ‘Basisregistratie Percelen’. View and download services for the most recent agricultural register are available at <https://data.overheid.nl/>.

- 1. **Soil maps**

The ALMaSS landscape simulator modifies the actual management on each field based on the dominant soil type, as soil cultivation on sandy soils is different than on clay soils for many crops.

We used soil maps for Holland available at https://data.overheid.nl/data/dataset/ngr-grondsoortenkaart-voor-het-mestbeleid-en-glb--versie-december-2017, where soils are classified into sandy, clay, loess and peat. Dominant soil type was mapped for each field parcel.


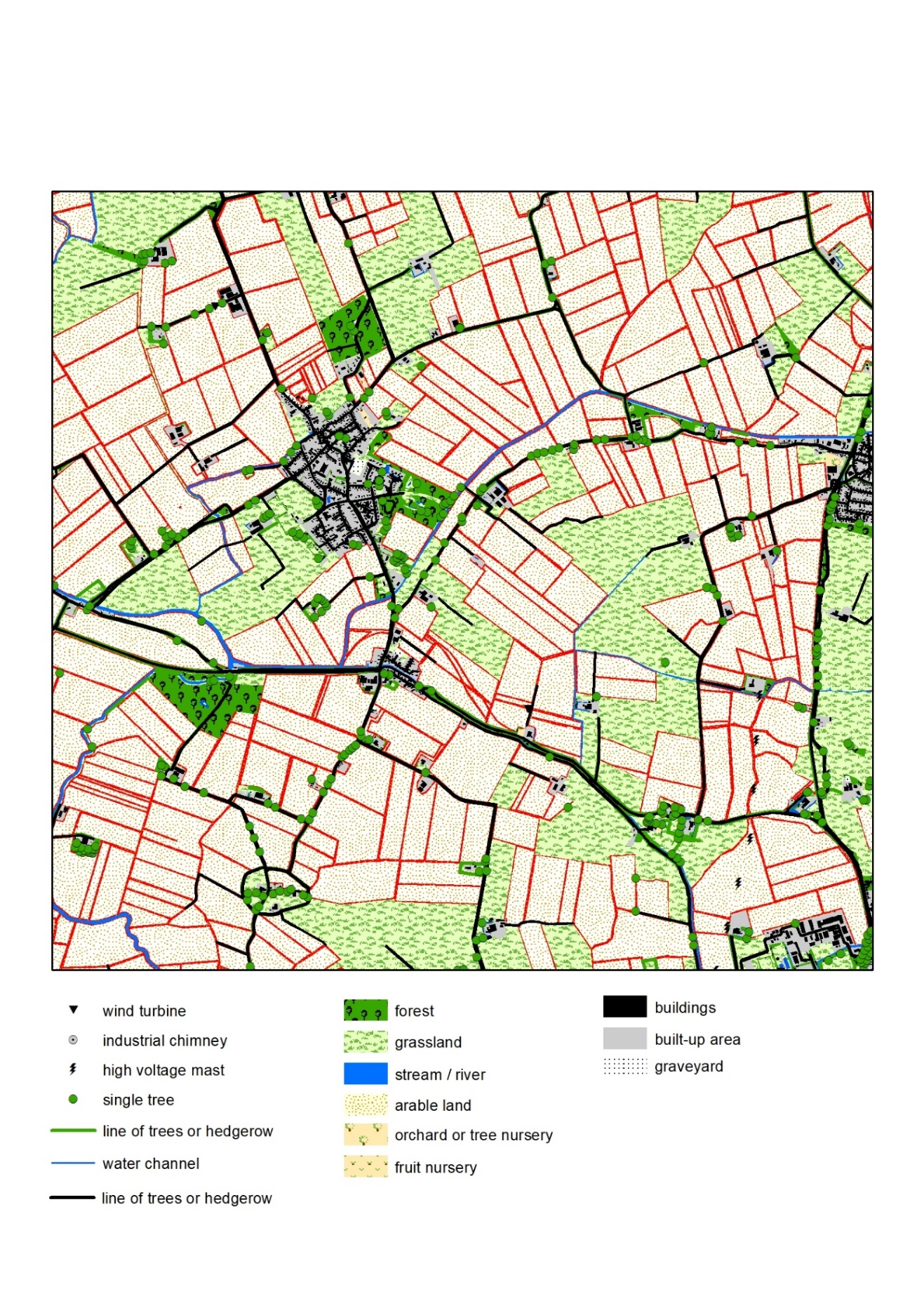


*Figure A1 Example of layers available from the TOP10NL database. Field parcels from the BRP are marked in red outlines*

1. **Generation of ALMaSS landscape map**

The aim of this task is to generate a landscape raster map of 1-m spatial resolution with complete coverage; hence all cells must be classified in accordance with their landscape element type. This was done by combining individual layers of land use / land cover information together with information on agricultural fields into a single raster landscape map in a step by step process. As layers from different data sources were used, this resulted in inconsistencies related to spatial alignment of features (overlaps or gaps between features). In addition, some objects were represented as points or lines and therefore as dimensionless had to be first pre-processed in order to change them into two-dimensional ones. However, this process increased the number of inconsistencies in the combined map even more, so a special step-by-step procedure was applied to be able to obtain a landscape raster map with no gaps in information and with removed sliver polygons.

The overall process to generate the ALMaSS landscape map consisted of the following steps:

1. Pre-processing of field parcel map;
2. Clipping the national datasets to the extent of a given study area of 10 km x 10 km;
3. Converting the input vector data to raster format (with spatial resolution of 1 m), object class by object class;
4. Combining individual layers into thematic maps (e.g., transportation theme, built-up theme),
5. Stacking of thematic maps to generate raw landscape map;
6. Removing of inconsistencies in the landscape raw map (multi-stage process);
7. Reclassification and regionalization of resulting landscape map;
8. Exporting results;
9. Generating reference files for ALMaSS.

All handling and analysis of spatial data were done using Python 2.7 (https://docs.python.org/2.7/) and the Python library arcpy to access ArcGIS features (ESRI 2010), or directly in ArcGIS 10.4. The entire process of producing Dutch landscape models for ALMaSS has been programmed in Python and R scripts:

- Script *landscape_NL_part1.py* covers points (2) – (5);
- Script *landscape_NL_part2.py* covers points (6) – (8);
- Script *ALMaSS_input_files_NL.r* covers point (9).
  1. **Pre-processing of field parcel map**

According to the agricultural register, field parcel map consists of both parcels managed as arable land (with different crops) and parcels with other, non-crop land cover types, such as forest or different types of field margins. All parcels with non-crop land cover types were treated as non-arable land and therefore non-managed. They were grouped according to land cover, and have assigned a classification ‘Code’ (Table A2).

- 1. **Processing the raw landscape map**

Steps (2-5) were programmed in the python script *landscape_NL_part1.py*. The first section of the script (*Setup*) describes the Python libraries, paths to input and output data, as well as processing environment. The second section of the script deals with clipping of the national datasets into a given study area. The third section of the script (*Conversion*) convert the original vector data into raster format. For linear and point features (such as streams or single trees), first the Euclidian distance from the feature was calculated, and then the cut-off value was applied to add the buffer (dimension) to the feature (Table A1).

The third section of the script (*Themes*) collects the raster layers into the following thematic maps: nature areas, water, cultivable, communication, built-up areas, and cultural features (Table A1). In cases where two or more of the layers in a theme overlap, the layer with the higher reclassification code is prioritized. The fourth section (*Stack*) stacks the thematic maps in a sequence such that the final map shows the ecological meaningful layers on top (result: ‘MapRaw’). The order of thematic layer was as follows: agricultural fields first -> other cultivable areas in an empty space -> natural dry areas in an empty space -> built-up areas in an empty space (without buildings) -> wet natural areas on top -> cultural features on top -> freshwater on top -> communication areas on top -> buildings on top -> sea added if necessary (Figure A2). After this process there was still number of cells without land cover type (i.e., ‘background’), as well as substantial number of ‘sliver’ polygons which need to be removed. These steps (6-8) were programmed in the python script *landscape_NL_part2.py*.


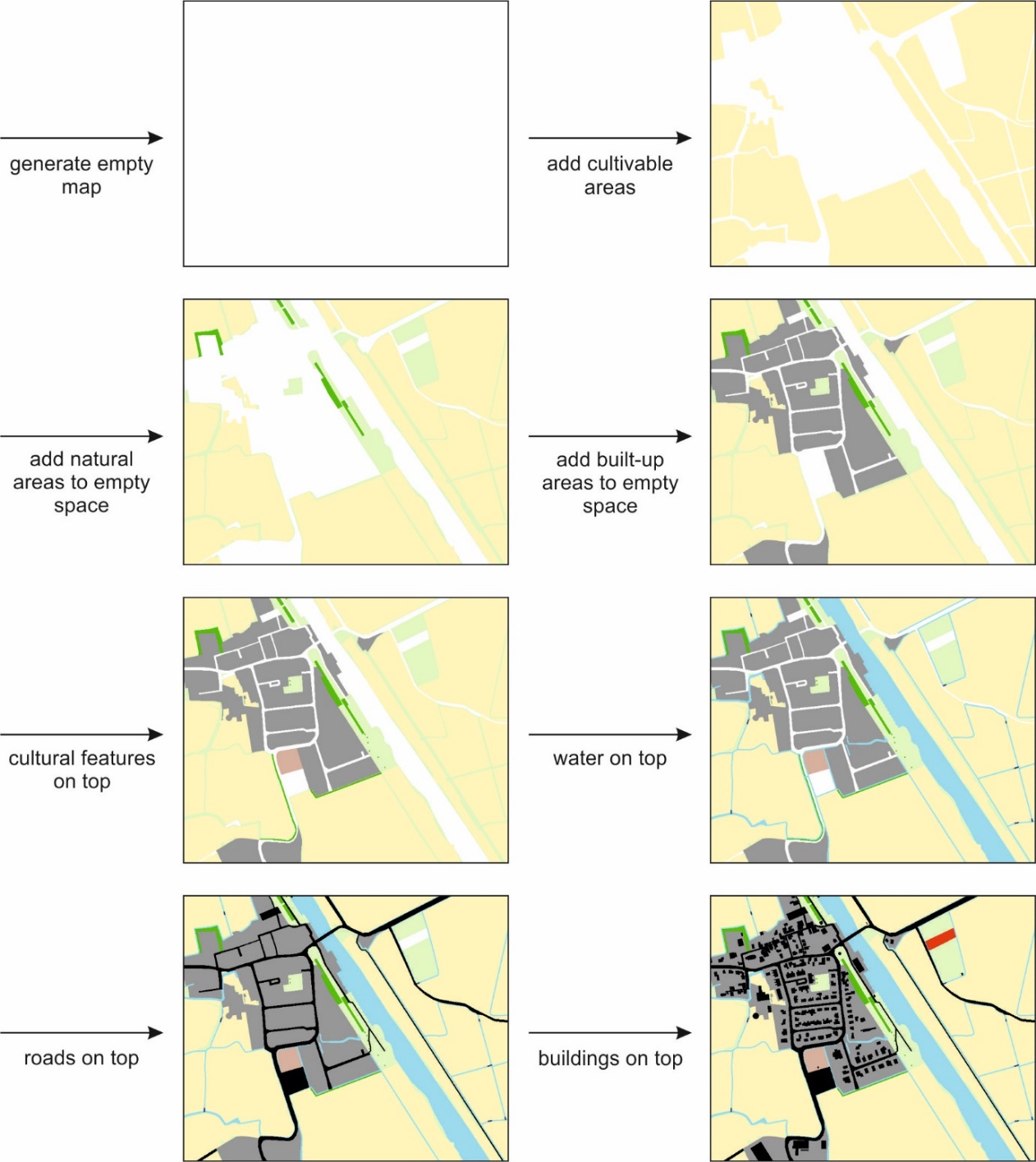


*Figure A2 Illustration of the stacking procedure. Non-classified areas (‘background’) is marked in red*

- 1. **Removing of inconsistencies in the raw landscape map**

The next step (6) was to get rid of sliver polygons and fill gaps (i.e., remove ‘background’ pixels with no landscape element type assigned) in order to generate landscape map with complete coverage. This was done in a multi-step process (programmed in script *landscape_NL_part2_new.py*):

1. Reclassification of big ‘background’ polygons (> 1ha) with at least 80% coverage of arable land according to the BDOT10k database into agricultural parcels (together with an update of agricultural parcel layers);
2. Removal of elongated sliver polygons of ‘background’, e.g., ones along road verges. ‘Background’ polygons for removal were selected as those for which ‘area/area of minimum bounding circle < 0.05 and area/length < 4’. Identified polygons were converted into field margins if neighboring with agricultural parcels, otherwise set as 'wasteland';
3. Elimination of small parts (< 1000 px) of artificially divided (by e.g. hedgerow or stream) field polygons;
4. Elimination of elongated sliver polygons of ‘background’ along rivers and their classification as ‘riverside plants’;
5. Filling the remaining ‘background’ gaps based on neighborhood analysis, i.e., gaps surrounded by built-up areas were classified as ‘yards’ (part of built-up areas), and the rest as ‘grassland’;
6. Cleaning of elongated sliver field polygons, e.g. left overs located between line of trees/hedgerow and road verge. Field polygons for elimination were selected as those for which ‘area/area of minimum bounding circle < 0.1 and area/length < 4’, and converted into ‘wasteland’.

Such procedure allowed for removal of most of problems we recognized on raw landscape map (i.e., after stacking; see examples on Figure A3).

- 1. **Reclassification and regionalization, exporting results**

Step (7) is finalizing the landscape as a final input map for ALMaSS. The landscape map contains more details than are used in ALMaSS. Therefore, to be consistent with landscape element types used in ALMaSS we used simple reclassification based on a text file (Table A3). All features in the ALMaSS landscape map, consisting both of single and multiple raster cells, have a unique value that is common to all cells within the feature. This was achieved by regionalizing the raster before exporting the map as a final ASCII file. Besides that, in this part of the script, attribute tables of landscape elements and agriculturally managed areas (including individual fields and permanent crops) are exported (step 8) to be further processed in step (9).

- 1. **Generating reference files for ALMaSS**

Script ALMaSS_files_prep.py was used to generate polygon and farm reference files for ALMaSS. As each polygon on the final ALMaSS landscape map only contains one value which is the unique ID of the polygon, all additional information on polygons need to be described in separate files. The polygon reference file is a text file containing unique IDs on all polygons in the landscape, with information of their landscape element type, the number of cells belonging to each polygon, a reference number to a farm owner/holding, and optionally the soil type of each polygon. The farm reference file is a text file relating farm reference numbers to farm types (see section 3).


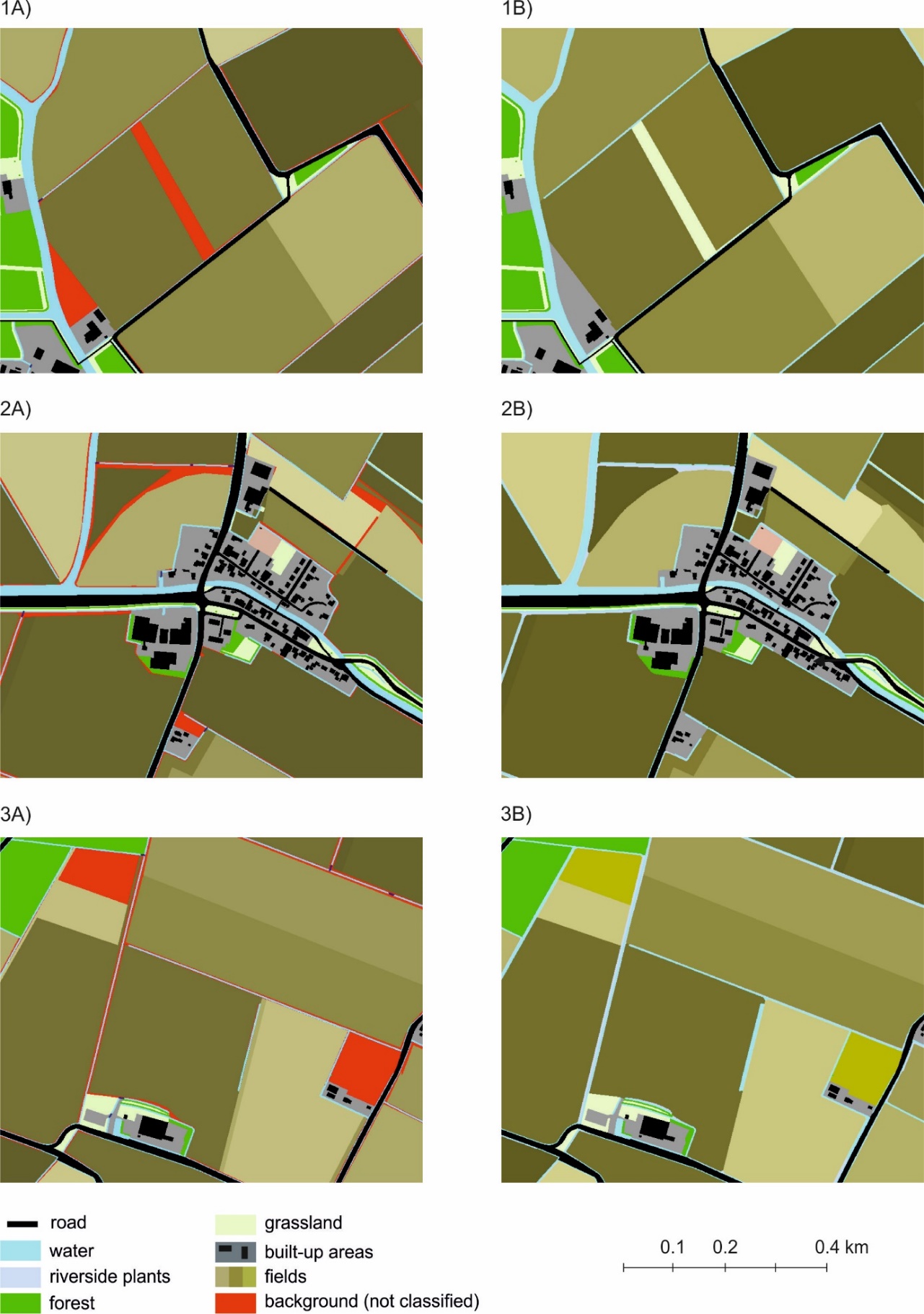


*Figure A3 example of sliver polygon elimination: (A) raw landscape map (before sliver polygon elimination), (B) final landscape map (after removal of sliver polygons)*

Table A1 Description of the individual layers used in the final landscape map, the theme in which they are grouped, and original data type. “Code” stands for the numerical value assigned to different objects in the second section of the script. “Cut-off” stands for the cut-off value which was used to add buffers to point and line objects

| **Layer** | **Description** | **Code** | **Theme** | **Type** | **cut-off [m]** |
| --- | --- | --- | --- | --- | --- |
| bos: gemengd bos | mixed forest | 511 | Natural | polygon |  |
| bos: loofbos | deciduous forest | 512 | Natural | polygon |  |
| bos: naaldbos | coniferous forest | 513 | Natural | polygon |  |
| bos: griend | river plants | 520 | Natural | polygon |  |
| grasland | grassland | 530 | Natural | polygon |  |
| populieren | poplars | 514 | Natural | polygon |  |
| basaltblokken, steenglooiing | stone flooring | 540 | Natural | polygon |  |
| heide | moorland | 550 | Natural | polygon |  |
| zand | sand | 560 | Natural | polygon |  |
|  | wasteland | 570 | Natural | polygon |  |
| waterloop | rivers (> 6m width) | 114 | Water | polygon |  |
| meer, plas, ven, vijver | lakes (> 80m2) | 120 | Water | polygon |  |
|  | lake buffer | 110 | Water | polygon | 2.05 |
| zee & droogvallend | see | 150 | Water | polygon |  |
| waterloop | water line, medium (3-6m) | 113 | Water | line | 2.25 |
| waterloop | water line, small (0.5-3m) | 112 | Water | line | 1.01 |
| greppel, droge sloot | dry channel | 111 | Water | line | 1.01 |
| dam, koedam | embankment/dike | 130 | Water | line | 1.2 |
| aanlegsteiger | quay | 141 | Water | line | 2 |
| aanlegsteiger | quay | 142 | Water | polygon |  |
|  | water buffer zone | 100 | Water | polygon | 1.5 |
| boomgaard | orchard | 610 | Cultivable | polygon |  |
| boomkwekerij | tree nursery | 620 | Cultivable | polygon |  |
| fruitkwekerij | fruit nursery | 630 | Cultivable | polygon |  |
| rand | field boundary | 900 | Cultivable | polygon |  |
| akkerland | arable land | 1000+ | Cultivable | polygon |  |
| wegdeel | area under large roads (>7m) | 219 | Road | polygon |  |
|  | verge of large road | 215 | Road | polygon | 2 |
|  | area under medium road (4-7m) | 218 | Road | polygon |  |
|  | verge of medium road | 214 | Road | polygon | 2 |
|  | area under small road (2-4m) | 217 | Road | polygon |  |
|  | verge of small road | 213 | Road | polygon | 1 |
|  | area under local, often unpaved road | 216 | Road | polygon |  |
| spoorbaanlichaam | area under railway including side verges, tracks | 220 | Road | polygon |  |
| fietsers, bromfietsers | bike road | 212 | Road | line | 1.01 |
| voetgangers | pedestrians | 211 | Road | line | 1.01 |
| spoorbaandeel | railway with 1 track | 221 | Road | line | 2.5 |
|  | railway with 2 track | 222 | Road | line | 4.5 |
|  | railway with 3 track | 223 | Road | line | 6.5 |
|  | railway with 4 track | 224 | Road | line | 8.5 |
| zendmast | transmission tower | 231 | Road | point | 2.5 |
| windturbine | wind turbines | 232 | Road | point | 1.5 |
| windmolentje | wind mill | 233 | Road | point | 4 |
| hoogspanningsmast | high voltage mast | 234 | Road | point | 1.5 |
| bebouwd gebied | build up areas | 411 | Building | polygon |  |
| overig | areas with bulildings on countryside | 412 | Building | polygon |  |
| bedrijventerrein | business park | 421 | Building | polygon |  |
| begraafplaats | cemetery | 422 | Building | polygon |  |
| bungalowpark | bungalow park | 423 | Building | polygon |  |
| camping, kampeerterrein | camping, camping | 423 | Building | polygon |  |
| caravanpark | caravan park | 423 | Building | polygon |  |
| dierentuin, safaripark | zoo, safari park | 424 | Building | polygon |  |
| erebegraafplaats | honorary cemetery | 422 | Building | polygon |  |
| golfterrein | golf course | 425 | Building | polygon |  |
| grafheuvel | burial mound | 422 | Building | polygon |  |
| grindwinning | gravel extraction | 429 | Building | polygon |  |
| groeve | quarry | 430 | Building | polygon |  |
| park | park | 427 | Building | polygon |  |
| plantsoen | park | 427 | Building | polygon |  |
| sportterrein, sportcomplex | sports field, sports complex | 426 | Building | polygon |  |
| stortplaats | dump | 431 | Building | polygon |  |
| tennispark | tennis park | 426 | Building | polygon |  |
| transformatorstation | transformer station | 436 | Building | polygon |  |
| tuincentrum | garden center | 437 | Building | polygon |  |
| vliegveld, luchthaven | airport, airport | 432 | Building | polygon |  |
| volkstuinen | allotments | 428 | Building | polygon |  |
| werf | yard | 428 | Building | polygon |  |
| windturbinepark | wind turbine park | 433 | Building | polygon |  |
| zandwinning | sand extraction | 434 | Building | polygon |  |
| zenderpark | transmitter park | 438 | Building | polygon |  |
| ziekenhuiscomplex | hospital complex | 439 | Building | polygon |  |
| zoutwinning | salt extraction | 435 | Building | polygon |  |
| zwembadcomplex | pool complex | 426 | Building | polygon |  |
| gebouw | buildings | 450 | Building | polygon |  |
| schoorsteen | indutrial chimney | 441 | Building | point | 1.5 |
| boom | tree | 321 | Cultural | point | 2 |
| heg, haag | hedgerow | 322 | Cultural | line | 2 |
| bomenrij | line of trees | 323 | Cultural | line | 2 |
| hekwerk | fence | 330 | Cultural | line | 1.01 |
| dodenakker | graveyard | 310 | Cultural | polygon |  |

Table A2 Description of non-crop (‘nature’) land cover types occurring in the agricultural register

| **Land cover NL** | **Land cover (translation)** | **Land cover group** |
| --- | --- | --- |
| Bomenrij en solitaire boom | Tree row and single tree | Tree row and single trees |
| Bos (SBL-regeling) | Forest (SBL-management) | Forest |
| Bos (set aside regeling) | Forest (set aside management) | Forest |
| Bos zonder herplantplicht | Forest without replanting obligation | Forest |
| Bos, blijvend, met herplantplicht | Permanent forest with replanting obligation | Forest |
| Bossingel en bosje | Coppice | Coppice |
| Elzensingel | Alder | Forest |
| Griendje | Coppice | Coppice |
| Hakhoutbosje | Grove | Forest |
| Houtwal en houtsingel | Grove | Forest |
| Knip- of scheerheg | Hedgerow | Hedgerow |
| Knotboom | Tree | Tree row and single trees |
| Natuurterreinen (incl. heide) | Nature | Nature |
| Natuurvriendelijke oever | River plants | River plants |
| Poel en klein historisch water | Pond | Pond |
| Rand, grenzend aan blijvend grasland of een blijvende teelt, hoofdzakelijk bestaand uit blijvend gras | Edge, adjacent to permanent grassland or permanent cultivation, mainly consisting of permanent grass | Field edge |
| Rand, grenzend aan blijvend grasland of een blijvende teelt, hoofdzakelijk bestaand uit een ander gew | Edge, adjacent to permanent grassland or permanent cultivation, mainly consisting of different plants | Field edge |
| Rand, grenzend aan blijvend grasland of een blijvende teelt, hoofdzakelijk bestaand uit tijdelijk gras | Edge, adjacent to permanent grassland or permanent cultivation, mainly consisting of temporary grass | Field edge |
| Rand, grenzend aan bouwland, hoofdzakelijk bestaand uit blijvend gras | Edge, adjacent to arable land, mainly consisting of permanent grass | Field edge |
| Rand, grenzend aan bouwland, hoofdzakelijk bestaand uit een ander gewas dan gras | Edge, adjacent to arable land, mainly consisting of a different crop than grass | Field edge |
| Rand, grenzend aan bouwland, hoofdzakelijk bestaand uit tijdelijk gras | Edge, adjacent to arable land, mainly consisting of temporary grass | Field edge |
| Sloot, grenzend aan beheerde akkerrand | Ditch, adjacent to managed field edge | Ditch |
| Struweelhaag | Hedgerow | Hedgerow |
| Wilgenhakhout | Willow | Forest |

Table A3 Reclassification values used to convert landscape element codes into codes used by ALMaSS

| **Layer** | **Code** | **ALMaSS code** |
| --- | --- | --- |
| mixed forest | 511 | 60 |
| deciduous forest | 512 | 40 |
| coniferous forest | 513 | 50 |
| river plants | 520 | 98 |
| grassland | 530 | 110 |
| poplars | 514 | 40 |
| stone flooring | 540 | 69 |
| moorland | 550 | 205 |
| sand | 560 | 101 |
| wasteland | 570 | 209 |
| rivers (> 6m width) | 114 | 96 |
| lakes (> 80m2) | 120 | 90 |
| lake buffer | 110 | 98 |
| see | 150 | 80 |
| water line, medium (3-6m) | 113 | 96 |
| water line, small (0.5-3m) | 112 | 207 |
| dry channel | 111 | 222 |
| embankment/dike | 130 | 140 |
| quay | 141 | 5 |
| water buffer zone | 100 | 226 |
| orchard | 610 | 56 |
| tree nursery | 620 | 214 |
| fruit nursery | 630 | 214 |
| field boundary | 900 | 160 |
| arable land | 1000+ | 20 |
| area under large roads | 219 | 121 |
| verge of large road | 215 | 13 |
| area under medium road | 218 | 121 |
| verge of medium road | 214 | 13 |
| area under small road | 217 | 122 |
| verge of small road | 213 | 13 |
| area under local, often unpaved road | 216 | 123 |
| area under railway including side verges, tracks | 220 | 13 |
| bike road | 212 | 123 |
| pedestrians | 211 | 123 |
| railway with 1 track | 221 | 118 |
| railway with 2 track | 222 | 118 |
| railway with 3 track | 223 | 118 |
| railway with 4 track | 224 | 118 |
| transmission tower | 231 | 212 |
| wind turbines | 232 | 211 |
| wind mill | 233 | 211 |
| high voltage mast | 234 | 212 |
| build up areas | 411 | 8 |
| areas with bulildings on countryside | 412 | 9 |
| business park | 421 | 16 |
| cemetery | 422 | 204 |
| bungalow park | 423 | 12 |
| camping, camping | 423 | 12 |
| caravan park | 423 | 12 |
| zoo, safari park | 424 | 14 |
| honorary cemetery | 422 | 204 |
| golf course | 425 | 12 |
| burial mound | 422 | 204 |
| gravel extraction | 429 | 115 |
| quarry | 430 | 115 |
| park | 427 | 14 |
| sports field, sports complex | 426 | 12 |
| dump | 431 | 224 |
| tennis park | 426 | 12 |
| transformer station | 436 | 8 |
| garden center | 437 | 214 |
| airport, airport | 432 | 12 |
| allotments | 428 | 12 |
| yard | 428 | 12 |
| wind turbine park | 433 | 9 |
| sand extraction | 434 | 115 |
| transmitter park | 438 | 9 |
| hospital complex | 439 | 16 |
| salt extraction | 435 | 115 |
| pool complex | 426 | 12 |
| buildings | 450 | 5 |
| indutrial chimney | 441 | 5 |
| tree | 321 | 213 |
| hedgerow | 322 | 130 |
| line of trees | 323 | 41 |
| fence | 330 | 225 |
| graveyard | 310 | 204 |

1. **Farm classification**

As original detailed farm classification from the Netherlands Enterprise Agency (Rijksdienst voor Ondernemend Nederland, RVO) included 38 different farm types, it was simplified (based on clustered analysis taking into account similarities in acreage of different crop types) to a number of 15 farm types (Table A4). Based on data on crops cultivated by farms of different types, crop rotation schemes were prepared for each farm type individually (Table A5). The crop rotation scheme consists of 100 crop entries with multiple entries of each crop type in accordance with typical agricultural farming practices. At the start of the simulation, a random crop in the rotation is taken as the starting point for each arable field and the next crop in the list is assumed to be grown in the same field in the following year. After four years, all fields of one farm type would have raised each of the 100 crops in the rotation list once. If a specific crop, e.g., maize for silage growth in the animal grazing farm, occurs 13 times out of 100 in the rotation (Table A5) it will on average occur on 13% of all fields covered by that rotation at any point in time.

Table A4 Classification of farm types in the Netherlands used in the landscape model in ALMaSS

| **Original farm type** | **Farm type after cluster analysis** |
| --- | --- |
| Animal combinations mainly grazing | Animal grazing |
| Animal combinations mainly livestock |  |
| Goat |  |
| Dairy |  |
| Laying hens farms for consumption eggs | Poultry |
| Other livestock |  |
| Other poultry |  |
| Cut flower | Flower |
| Flower bulb |  |
| Other horticulture |  |
| Arable / grazing livestock combinations | Arable grazing |
| Other crops / animal combinations |  |
| Broiler |  |
| Other pig | Pig and calf |
| Breeding sows |  |
| Meat calf |  |
| Arable vegetable mainly feed crop |  |
| Starch potato | Starch potato |
| Wheat, oilseed and protein crop | Wheat |
| Arable vegetable | Arable vegetable |
| Crop combinations |  |
| Other arable |  |
| Horse and pony | Horse and sheep |
| Other grazing livestock |  |
| Sheep |  |
| Grazing livestock farms with mainly feed crops |  |
| Other cattle |  |
| Fruit | Fruit |
| Greenhouse vegetable | Greenhouse vegetable |
| Other greenhouse vegetable |  |
| Open field vegetable | Vegetable |
| Other permanent crop | Permanent crop |
| Tree nursery |  |
| Vineyards | Vineyards |
| Mushroom | Other |
| Not classified |  |
| Pot and bedding plant |  |

**References**

Topping, C.J., Dalby. L., Skov, F., 2016. Landscape structure and management alter the outcome of a pesticide ERA: Evaluating impacts of endocrine disruption using the ALMaSS European Brown Hare model. Science of the Total Environment, 541: 1477-1488.

Table A5 The proportion of each crop or group of crops assumed to be grown by each farm type in the Netherlands

| **Farm ref. no.** | **32** | **33** | **34** | **35** | **36** | **37** | **38** | **39** | **40** | **41** | **42** | **43** | **44** | **45** | **46** |
| --- | --- | --- | --- | --- | --- | --- | --- | --- | --- | --- | --- | --- | --- | --- | --- |
| **Farm type / Crop** | **Animal grazing** | **Arable grazing** | **Arable vegetable** | **Flower** | **Fruit** | **Greenhouse vegetable** | **Horse and sheep** | **Other** | **Pig and calf** | **Poultry** | **Starch potato** | **Vegetable** | **Vineyards** | **Wheat** | **Permanent crops** |
| **Winter wheat** | 1 | 13 | 25 | 3 | 4 | 6 | 1 | 1 | 2 | 12 | 6 | 2 | 5 | 64 | 1 |
| **Spring barley** | 0 | 4 | 5 | 3 | 1 | 6 | 1 | 0 | 2 | 4 | 20 | 4 | 0 | 11 | 0 |
| **Maize for silage** | 13 | 17 | 8 | 6 | 3 | 10 | 9 | 7 | 42 | 24 | 5 | 9 | 14 | 10 | 7 |
| **Carrots** | 0 | 5 | 11 | 4 | 1 | 20 | 0 | 1 | 1 | 3 | 0 | 23 | 0 | 1 | 1 |
| **Cabbage** | 0 | 1 | 2 | 3 | 0 | 7 | 0 | 0 | 0 | 1 | 1 | 31 | 0 | 0 | 0 |
| **Potatoes** | 1 | 19 | 25 | 5 | 2 | 4 | 0 | 3 | 4 | 10 | 49 | 5 | 0 | 1 | 3 |
| **Beet** | 0 | 7 | 10 | 2 | 1 | 1 | 0 | 1 | 2 | 4 | 14 | 2 | 0 | 5 | 1 |
| **Permanent grassland grazed** | 59 | 13 | 2 | 3 | 3 | 10 | 56 | 8 | 29 | 25 | 1 | 2 | 14 | 2 | 8 |
| **Grassland in rotation grazed** | 25 | 14 | 6 | 3 | 2 | 12 | 15 | 4 | 13 | 12 | 2 | 5 | 3 | 4 | 4 |
| **Tulips** | 0 | 1 | 1 | 59 | 0 | 6 | 0 | 1 | 0 | 0 | 0 | 0 | 0 | 0 | 1 |
| **Fruits** | 0 | 0 | 0 | 1 | 5 | 6 | 0 | 0 | 0 | 0 | 0 | 9 | 0 | 0 | 0 |
| **Orchards** | 0 | 1 | 1 | 1 | 78 | 3 | 0 | 12 | 0 | 1 | 0 | 1 | 1 | 0 | 12 |
| **Tree plantations** | 0 | 0 | 0 | 0 | 0 | 0 | 0 | 0 | 0 | 0 | 0 | 0 | 0 | 0 | 0 |
| **Vineyards** | 0 | 0 | 0 | 0 | 0 | 0 | 0 | 0 | 0 | 0 | 0 | 0 | 62 | 0 | 0 |
| **Permanent Set Aside** | 0 | 0 | 0 | 0 | 0 | 0 | 0 | 12 | 0 | 0 | 0 | 0 | 0 | 0 | 12 |
| **Young forest** | 0 | 0 | 0 | 0 | 0 | 0 | 0 | 22 | 0 | 0 | 0 | 0 | 0 | 0 | 22 |
| **Other** | 1 | 5 | 4 | 5 | 1 | 8 | 17 | 27 | 6 | 3 | 3 | 6 | 1 | 3 | 27 |
