# Supplementary material for: Is the effectiveness of policy-driven mitigation measures on carabid populations driven by landscape and farmland heterogeneity? Applying a modelling approach in the Dutch agroecosystems
Source: PLoS One. 2022 Dec 27;17(12):e0279639. doi: 10.1371/journal.pone.0279639 (PMC9794068; doi:10.1371/journal.pone.0279639)
Supplement: S2 Appendix — (DOCX) [file pone.0279639.s002.docx]

# Appendix B. Structural and farming heterogeneity of studied landscapes

Table B1 Summary of structural and farming heterogeneity metrics for studied landscapes

|  | **Study area** | **Landscape heterogeneity** | | | | **Farming heterogeneity** | | | | |
| --- | --- | --- | --- | --- | --- | --- | --- | --- | --- | --- |
|  | **Share of herbaceous semi-natural habitats** | **Share of woody semi-natural habitats** | **Landscape diversity** | **Landscape shape index** | **Farming diversity** | **Share of animal farms** | **Number of fields** | **Mean field size** | **Field margins density** |  |
| **1** | 4.6 | 1.3 | 0.71 | 134.6 | 1.07 | 53 | 2243 | 3.6 | 0.028 |  |
| **2** | 6.8 | 1.5 | 0.93 | 156.6 | 1.40 | 33 | 2454 | 2.9 | 0.036 |  |
| **3** | 9.1 | 4.2 | 0.99 | 141.3 | 1.41 | 24 | 1864 | 3.8 | 0.033 |  |
| **4** | 5.1 | 5.2 | 0.91 | 98.5 | 0.96 | 10 | 1642 | 4.5 | 0.025 |  |
| **5** | 6.4 | 5.9 | 0.78 | 93.6 | 1.10 | 14 | 1195 | 6.7 | 0.025 |  |
| **6** | 7.2 | 6.5 | 1.04 | 150.9 | 0.83 | 85 | 3070 | 2.2 | 0.038 |  |
| **7** | 7.5 | 9.0 | 1.08 | 100.5 | 1.59 | 43 | 3718 | 1.7 | 0.044 |  |
| **8** | 6.4 | 12.5 | 1.10 | 128.7 | 1.60 | 51 | 2712 | 2.3 | 0.036 |  |
| **9** | 11.7 | 9.8 | 1.35 | 170.9 | 1.81 | 53 | 3330 | 1.4 | 0.048 |  |
| **10** | 6.1 | 1.5 | 0.81 | 98.2 | 0.81 | 8 | 1998 | 3.9 | 0.030 |  |

Table B2 Classification of landscape metrics’ values into classes

| **Share of semi-natural habitats [%]** | | **Landscape shape index (LSI)** | | **Landscape diversity** | | **Farm type diversity** | | **Share of animal farms [%]** | | **Fields fragmentation** |
| --- | --- | --- | --- | --- | --- | --- | --- | --- | --- | --- |
| values | class | values | class | values | class | values | class | values | class | Classes defined based on combined values of no. of fields, mean field size & density of field boundaries (see figure D1 below). |
| < 10 | low | < 120 | low | < 0.85 | low | < 1.00 | low | < 40 | low |  |
| 10 - 15 | medium | 120 - 160 | medium | 0.85 - 1.20 | medium | 1.00 - 1.50 | medium | 40 - 60 | medium |  |
| > 15 | high | > 160 | high | > 1.20 | high | > 1.50 | high | > 60 | high |  |

Table B3 Classes of landscape metrics in analyzed study areas

| **Metric** | **Study area** | | | | | | | | | |  |
| --- | --- | --- | --- | --- | --- | --- | --- | --- | --- | --- | --- |
|  | **1** | **2** | **3** | **4** | **5** | **6** | **7** | **8** | **9** | **10** |  |
| ***Landscape heterogeneity*** | | | | | | | | | | | |
| Share of semi-natural habitats [%] | low | low | medium | medium | medium | medium | high | high | high | low |  |
| Landscape shape index (LSI) | medium | medium | medium | low | low | medium | low | medium | high | low |  |
| Landscape diversity | low | medium | medium | medium | low | medium | medium | medium | high | low |  |
| ***Farming heterogeneity*** | | | | | | | | | | |  |
| Farming diversity | medium | medium | medium | low | medium | low | high | high | high | low |  |
| Share of animal farms | medium | low | low | low | low | high | medium | medium | medium | low |  |
| Fields fragmentation* | medium | medium | medium | low | low | high | high | medium | high | medium |  |


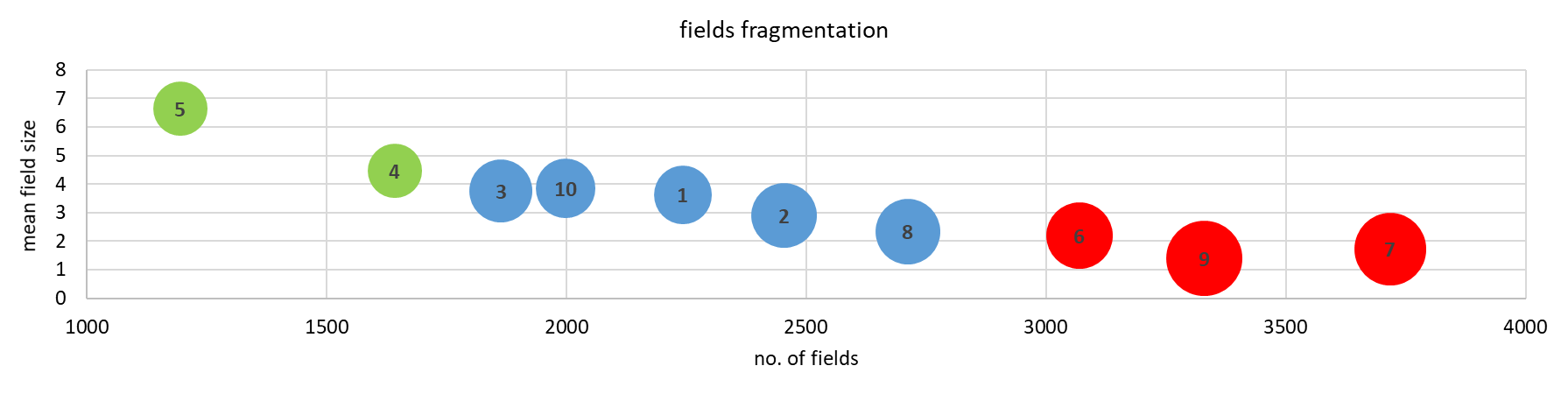


low fragmentation

medium fragmentation

high fragmentation

Figure B1 Relationship between no. of fields, mean field size and density of field boundaries in analyzed study areas (numbers of study areas are provided in circles). The size of the circle indicates the density of field boundaries (bigger circle = higher density). Colors of circle indicates general fields fragmentation: low (green) for landscapes with small number of big fields, medium (blue) for landscapes with low to medium number of fields and mean field sizes, and high (red) for landscapes with high no. of fields and low to medium mean field sizes

**Visual overview of analyzed study areas**

A)


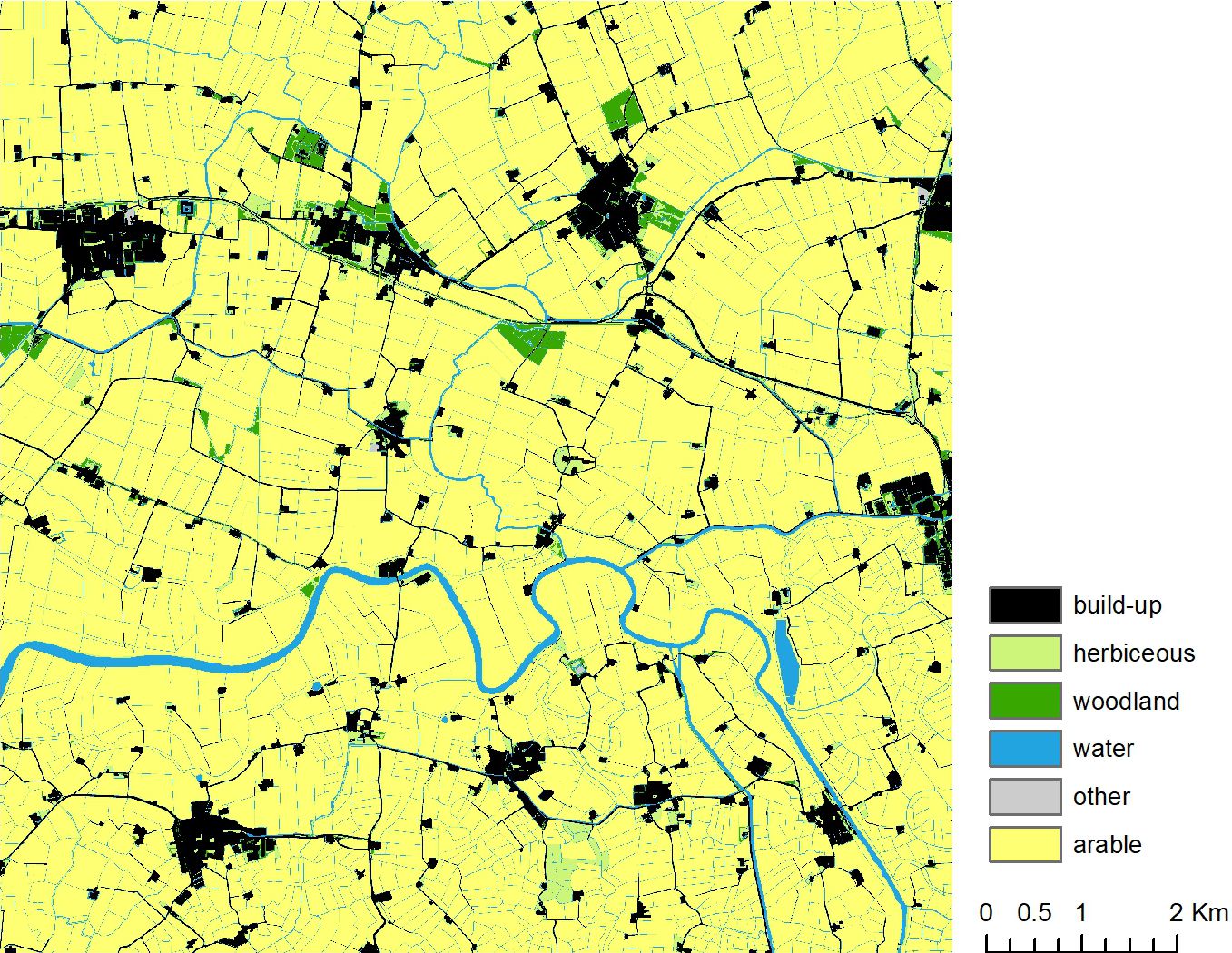


B)

**
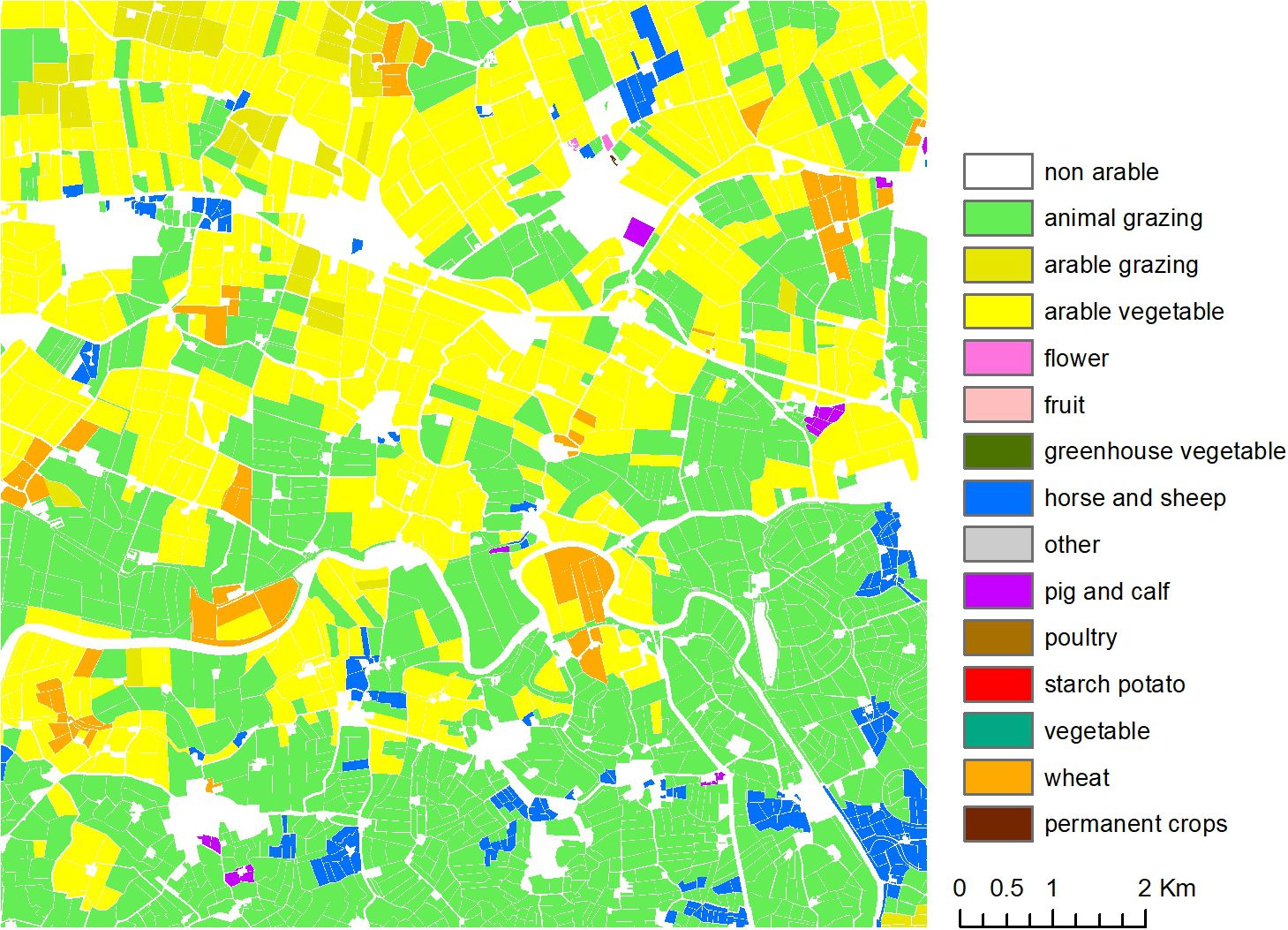
**

*Figure B2 Visual overview of study area 1. 10x10 km. mapping the basic elements of the landscape visible at this scale (A). and farm types according to classification presented in Appendix A. Table A4 (B)*

A)


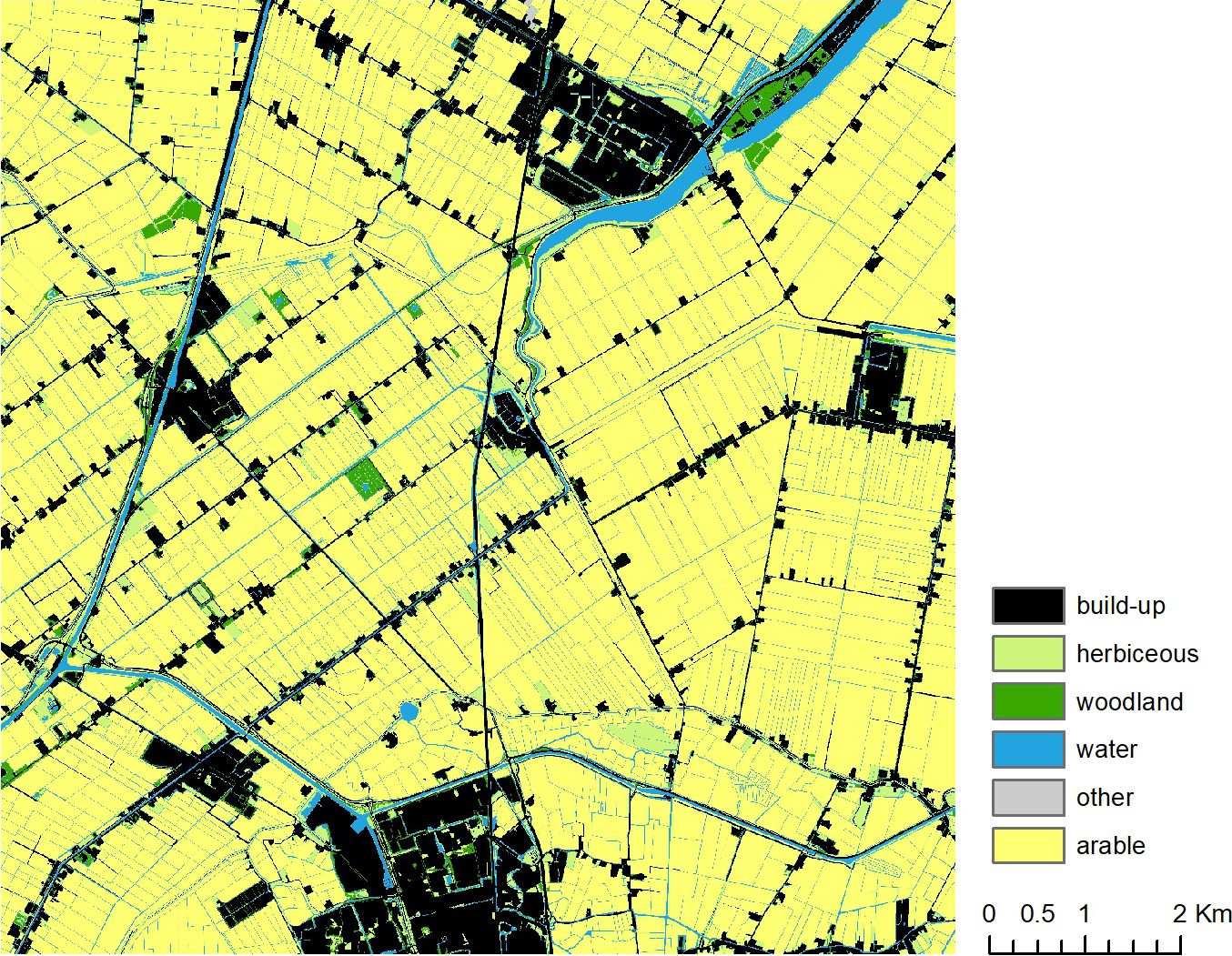


B)


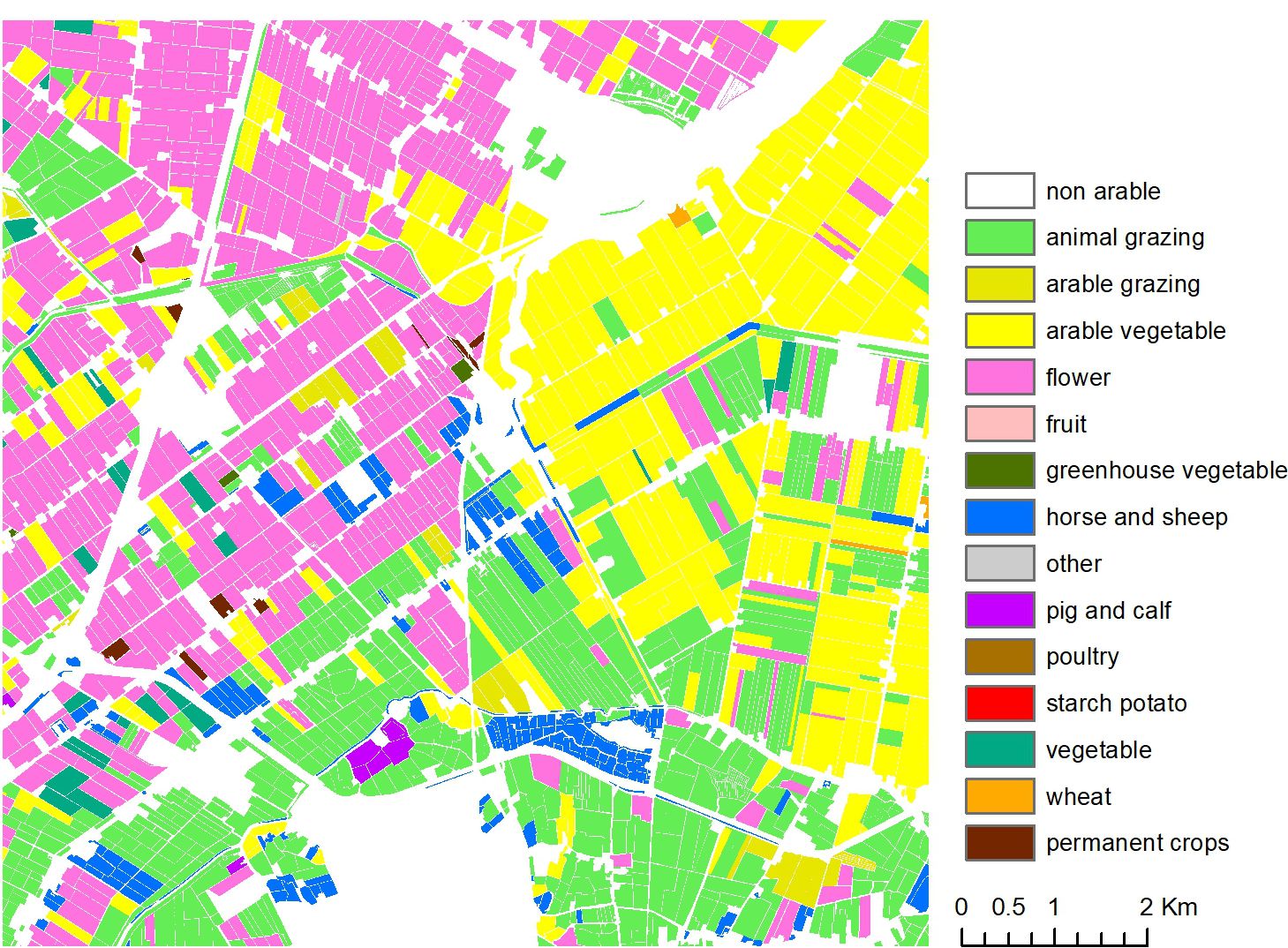


*Figure B3 Visual overview of study area 2. 10x10 km. mapping the basic elements of the landscape visible at this scale (A). and farm types according to classification presented in Appendix A. Table A4 (B)*

A)


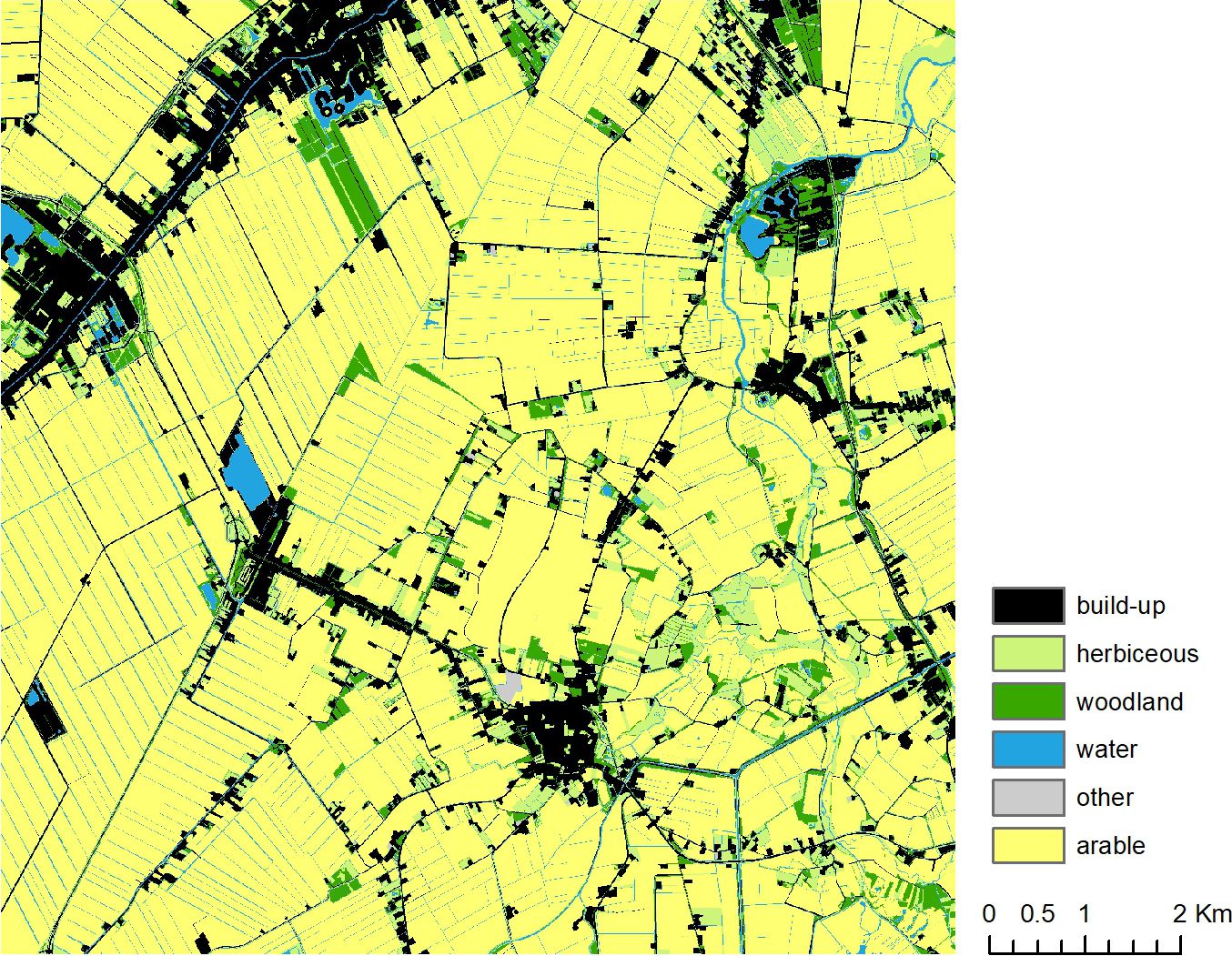


B)


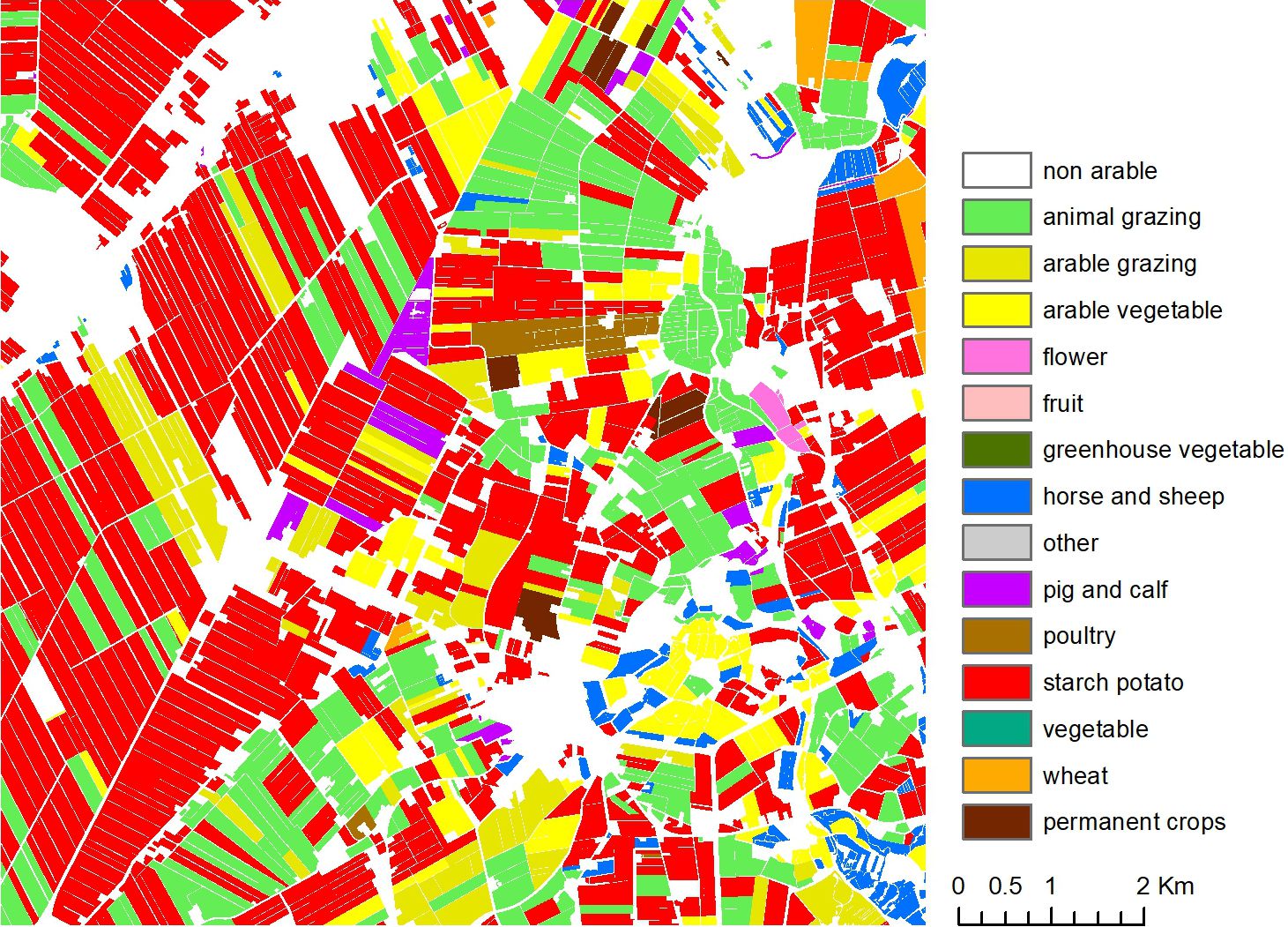


*Figure B4 Visual overview of study area 3. 10x10 km. mapping the basic elements of the landscape visible at this scale (A). and farm types according to classification presented in Appendix A. Table A4 (B)*

A)


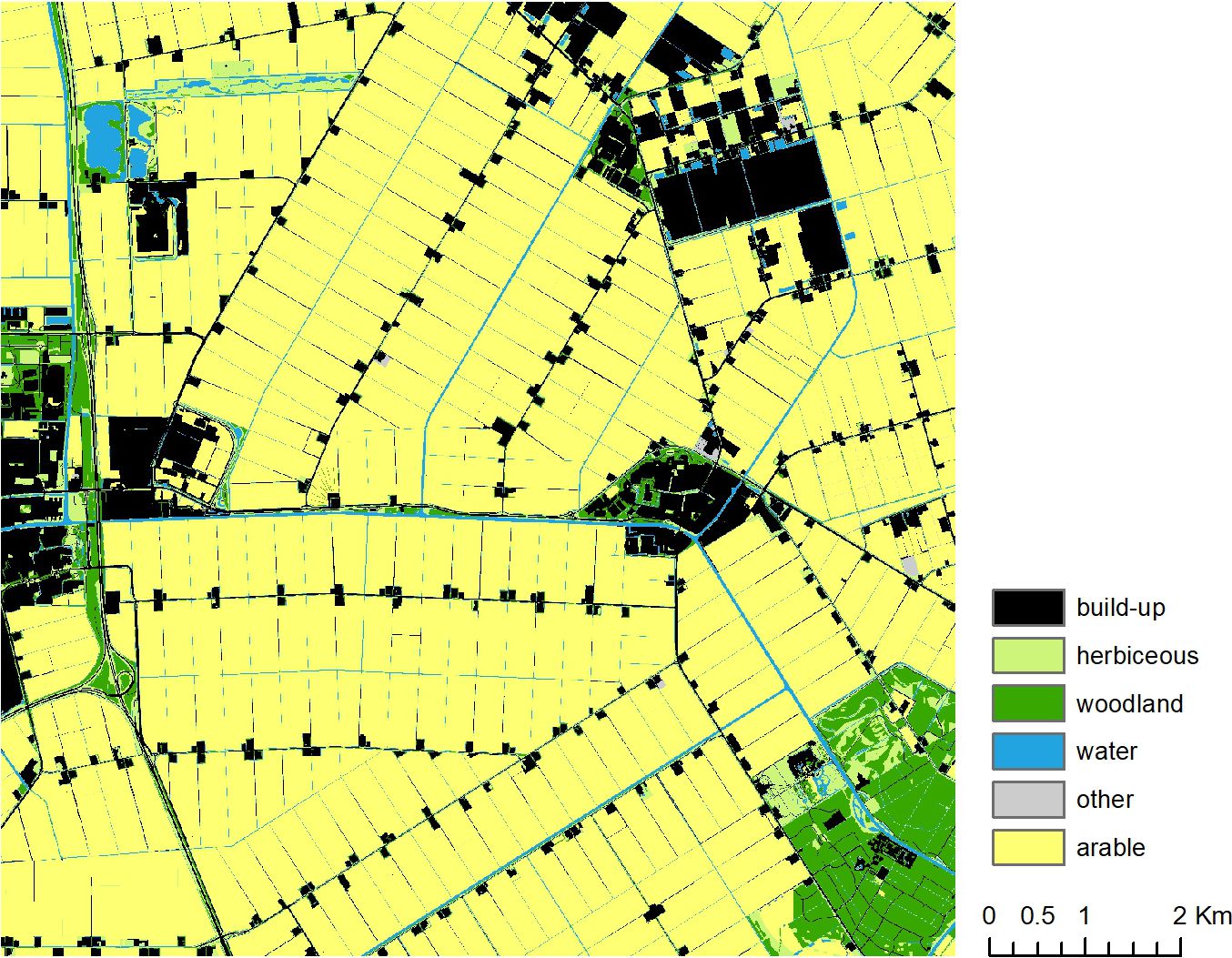


B)


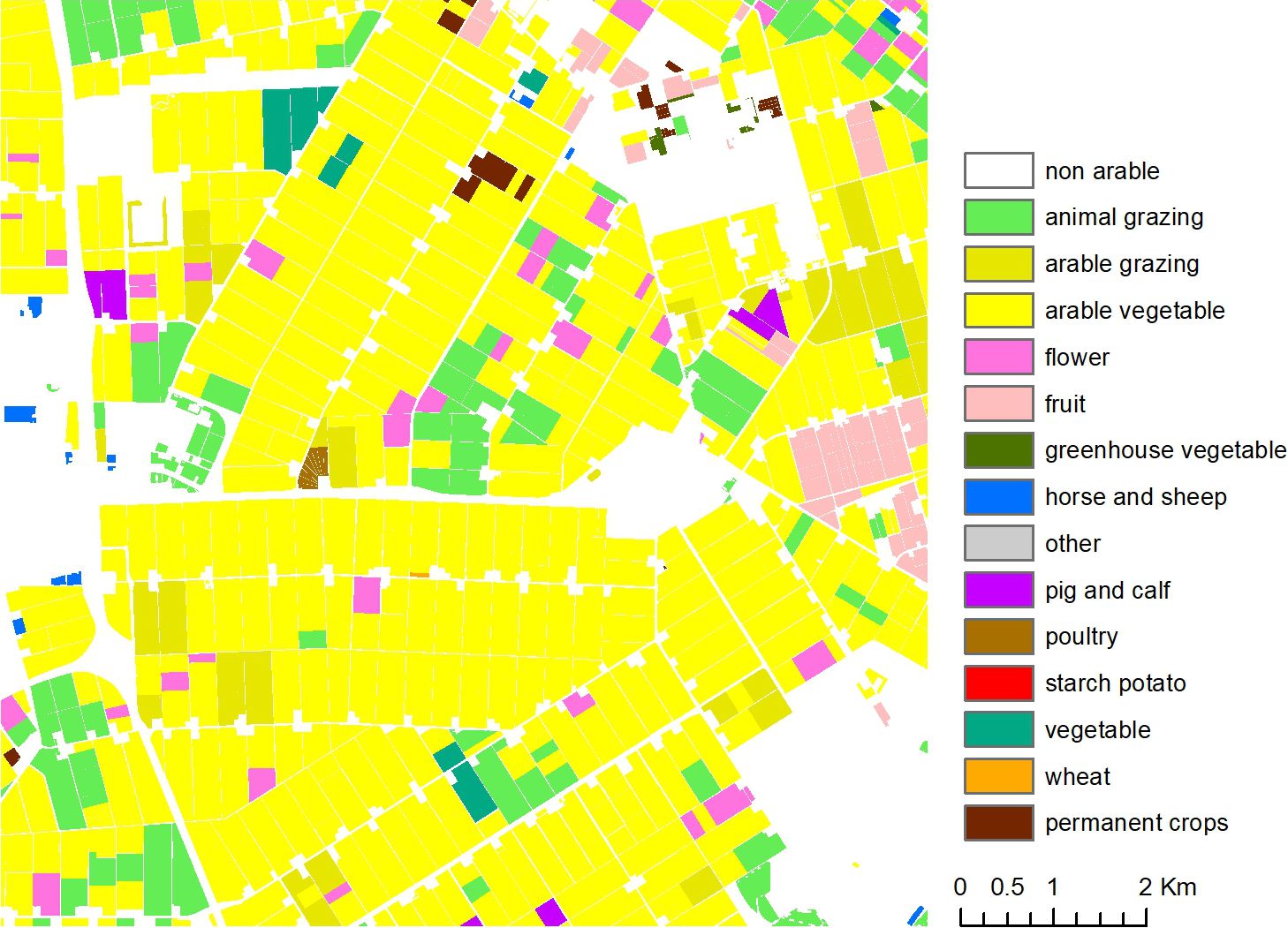


*Figure B5 Visual overview of study area 4. 10x10 km. mapping the basic elements of the landscape visible at this scale (A). and farm types according to classification presented in Appendix A. Table A4 (B)*

A)


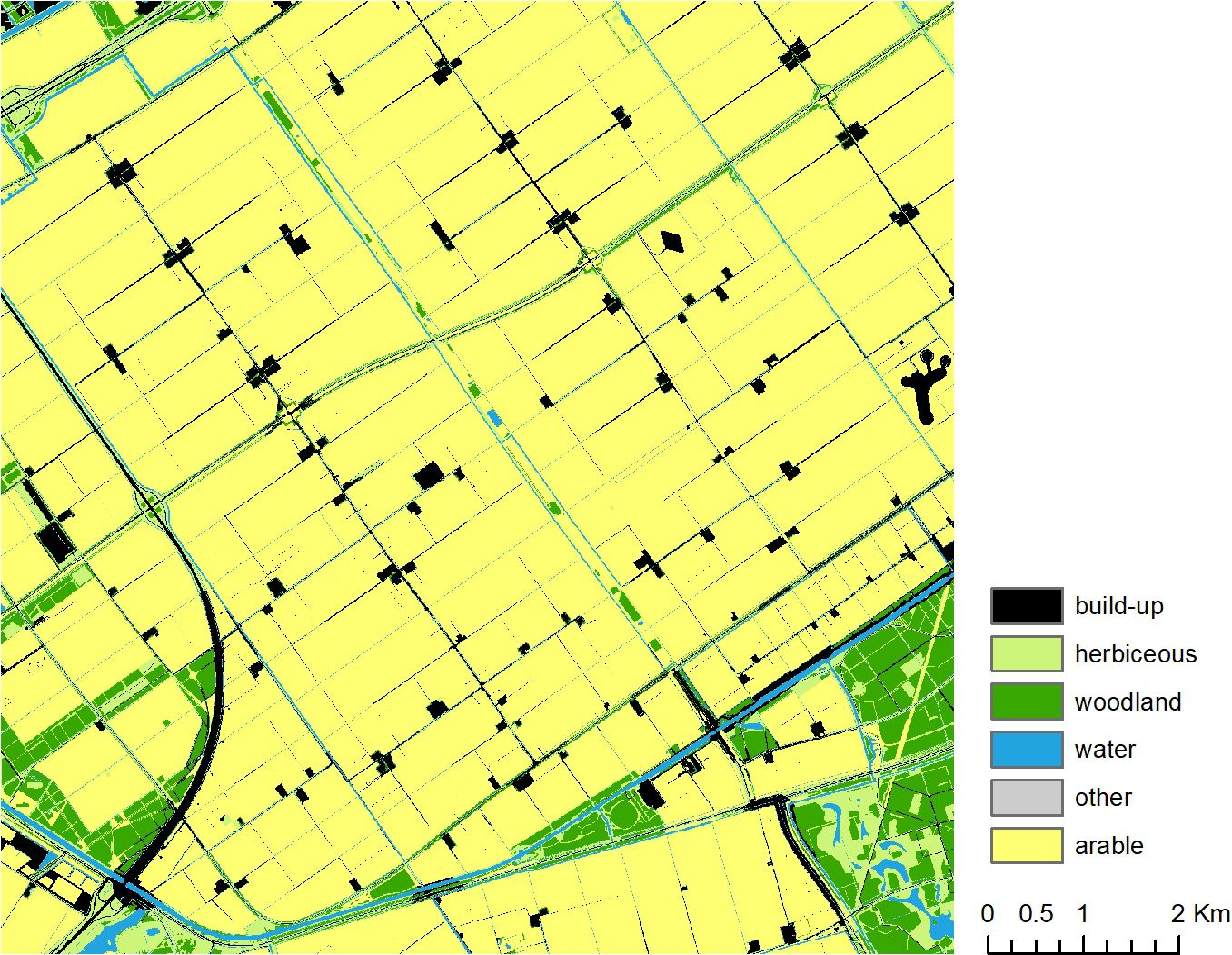


B)


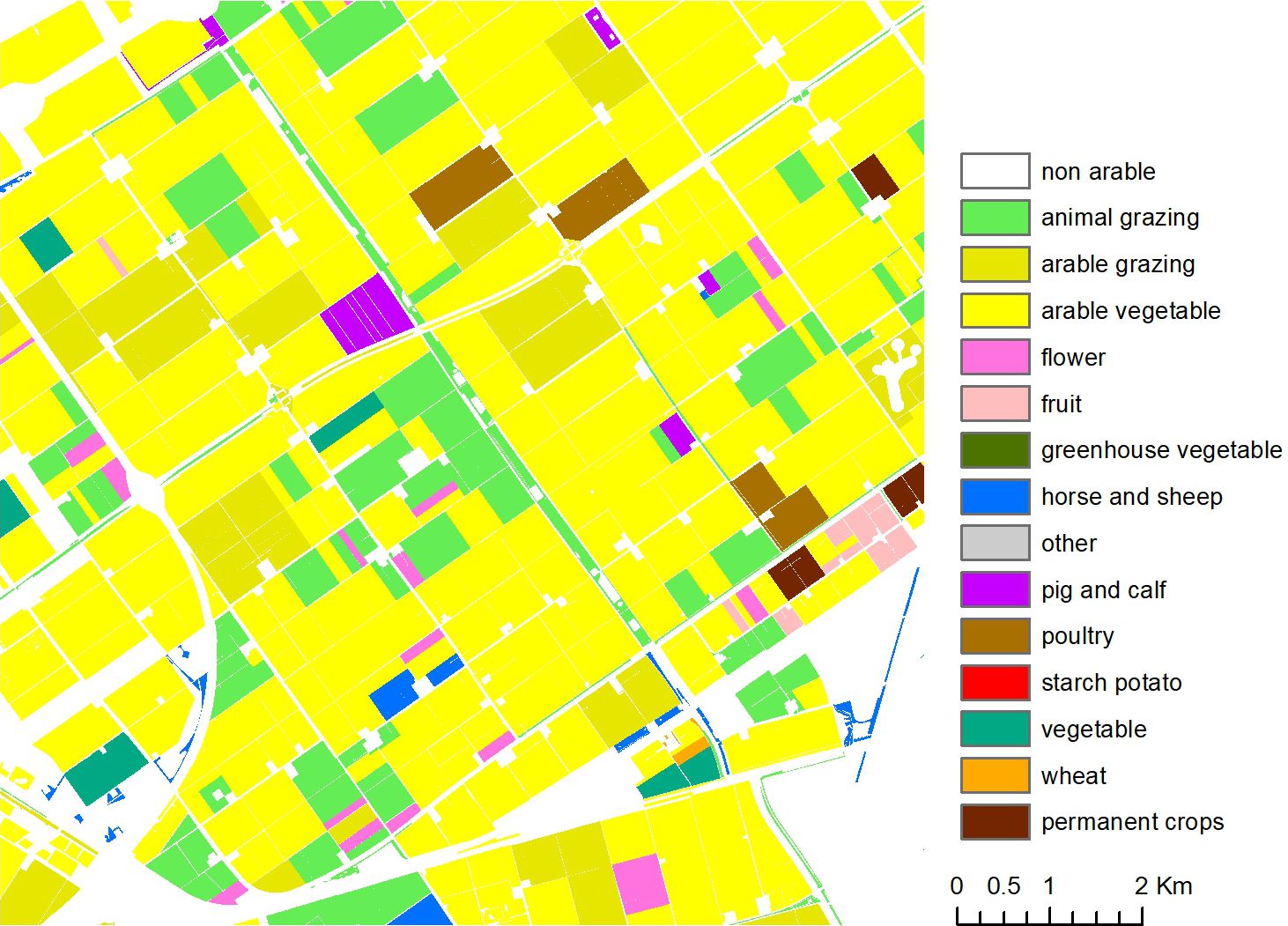


*Figure B6 Visual overview of study area 5. 10x10 km. mapping the basic elements of the landscape visible at this scale (A). and farm types according to classification presented in Appendix A. Table A4 (B)*

A)


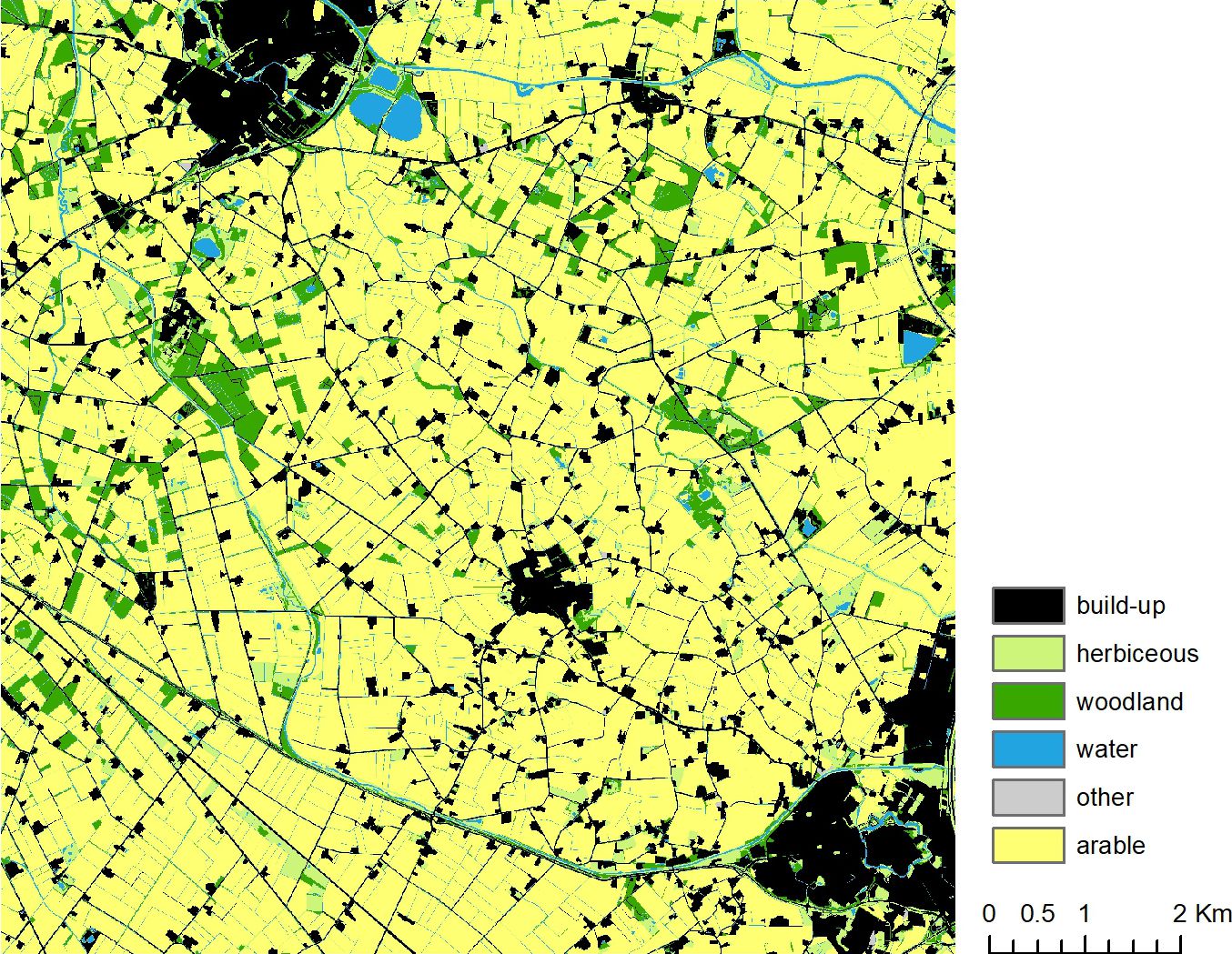


B)


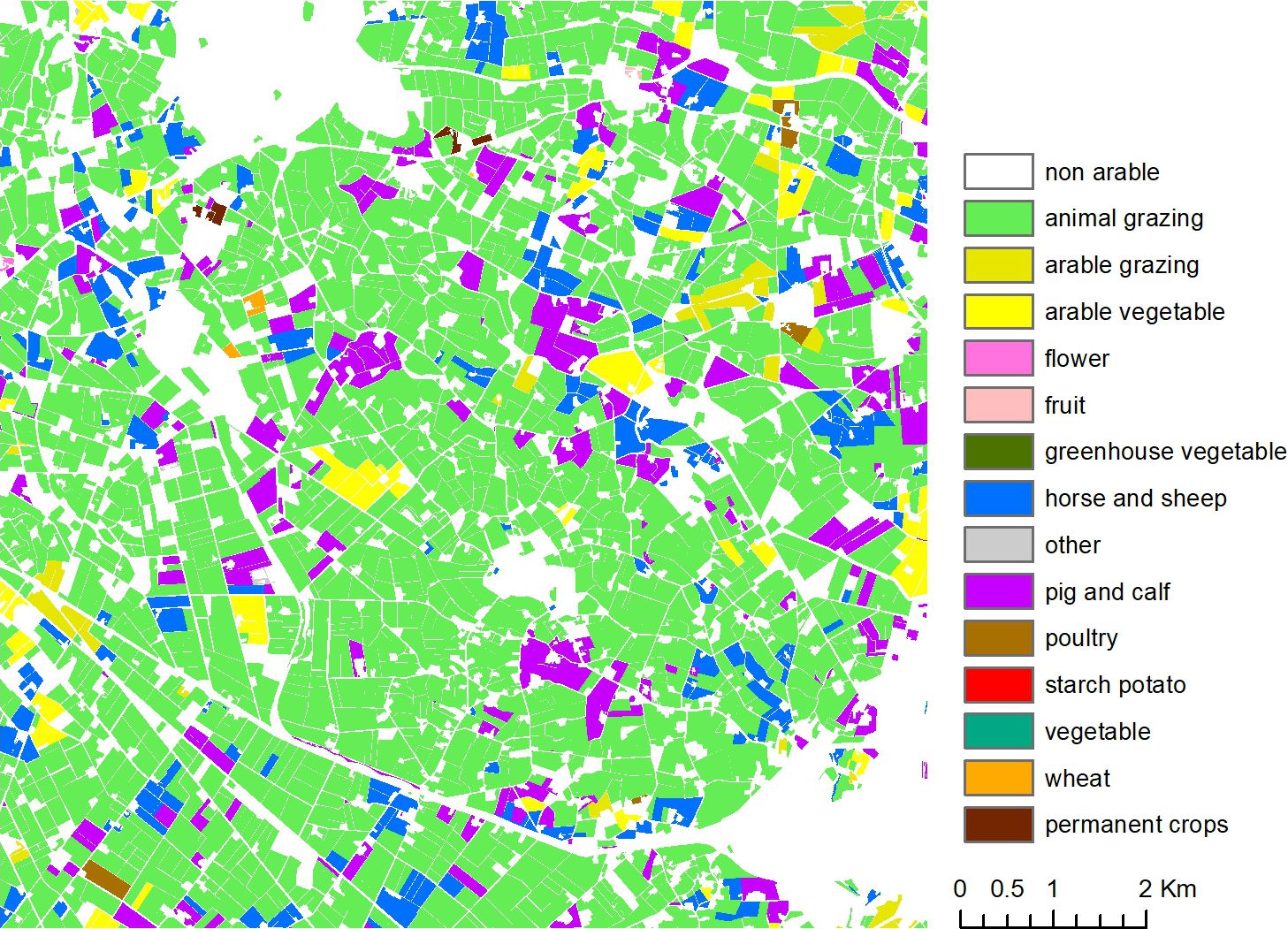


*Figure B7 Visual overview of study area 6. 10x10 km. mapping the basic elements of the landscape visible at this scale (A). and farm types according to classification presented in Appendix A. Table A4 (B)*

A)


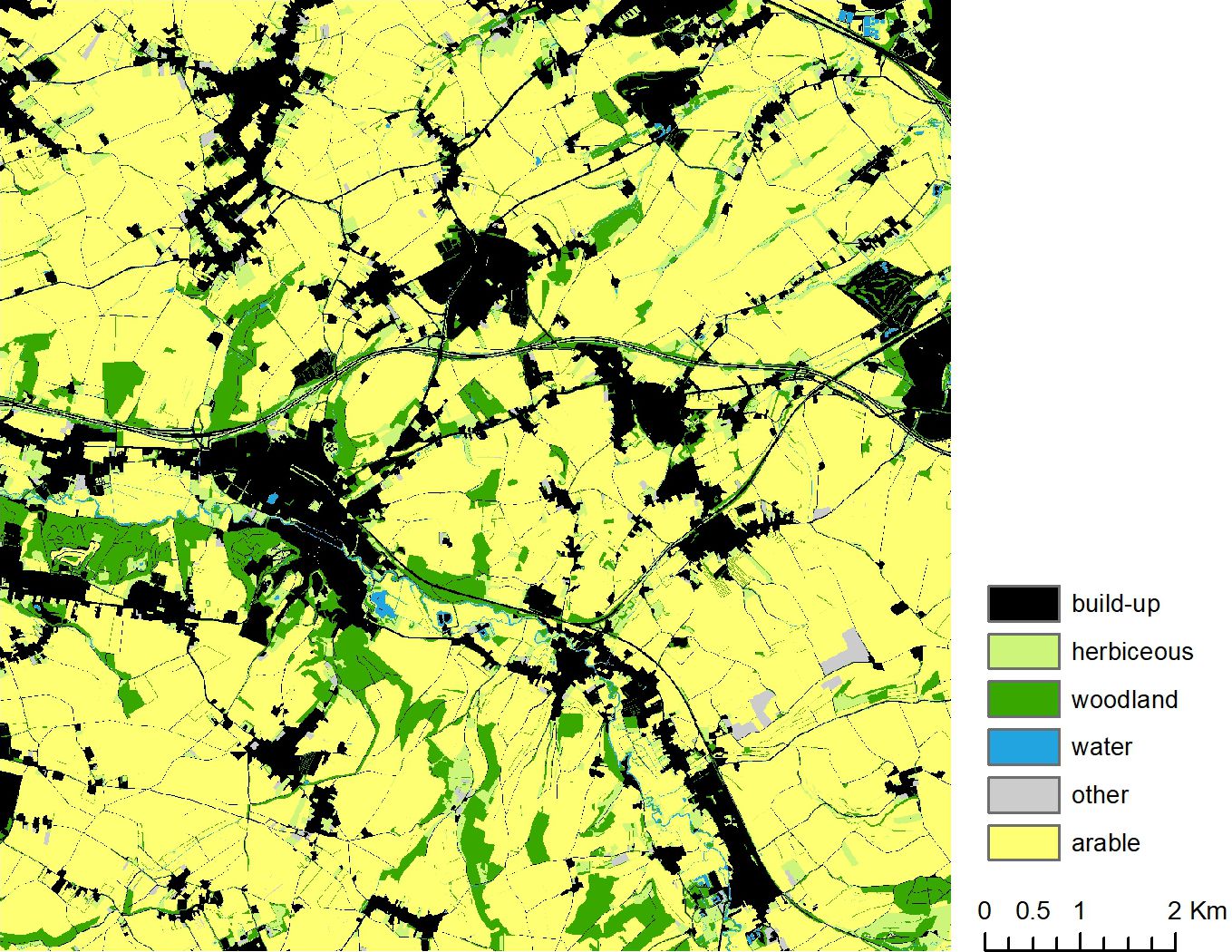


B)


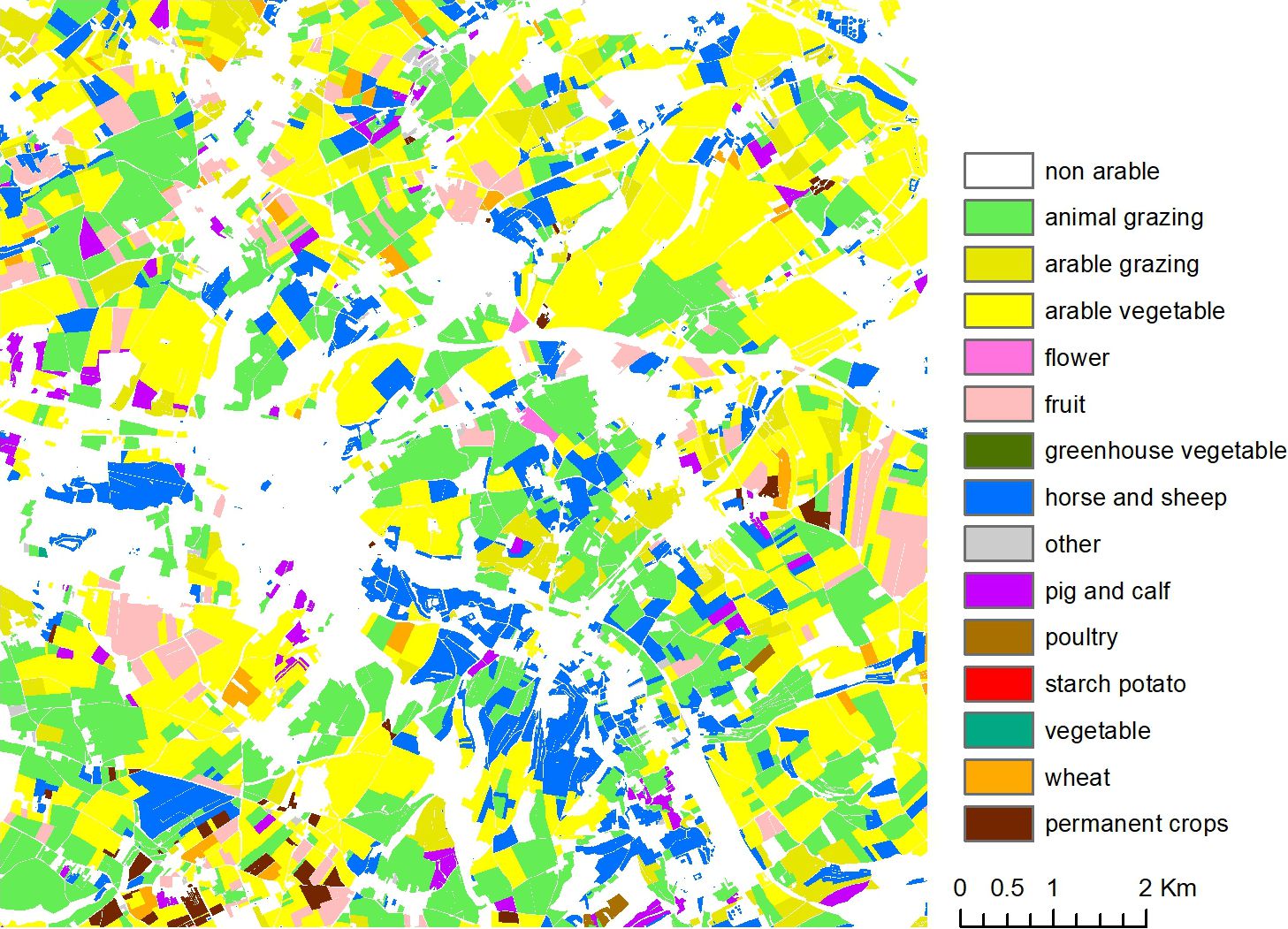


*Figure B8 Visual overview of study area 7. 10x10 km. mapping the basic elements of the landscape visible at this scale (A). and farm types according to classification presented in Appendix A. Table A4 (B)*

A)


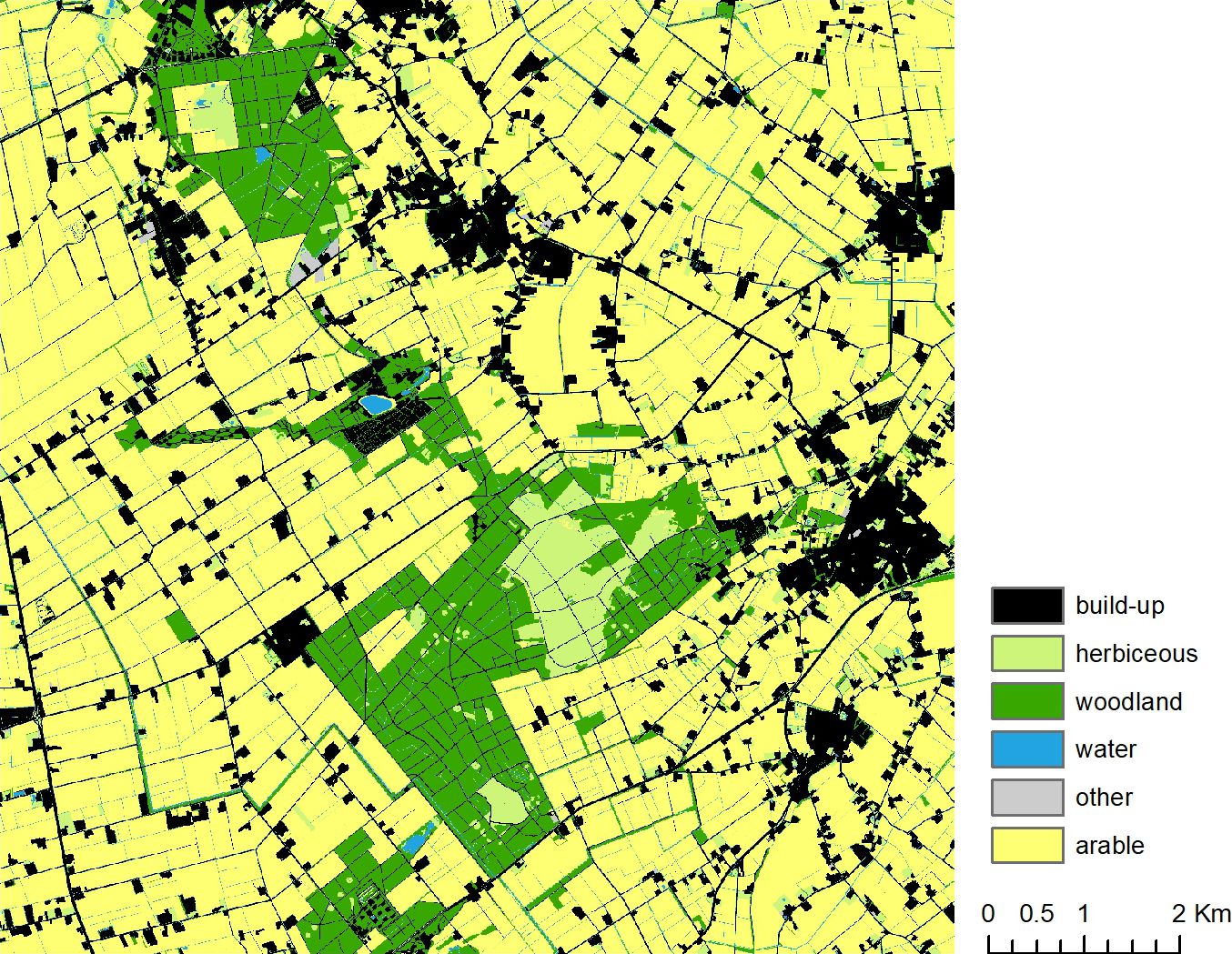


B)

*
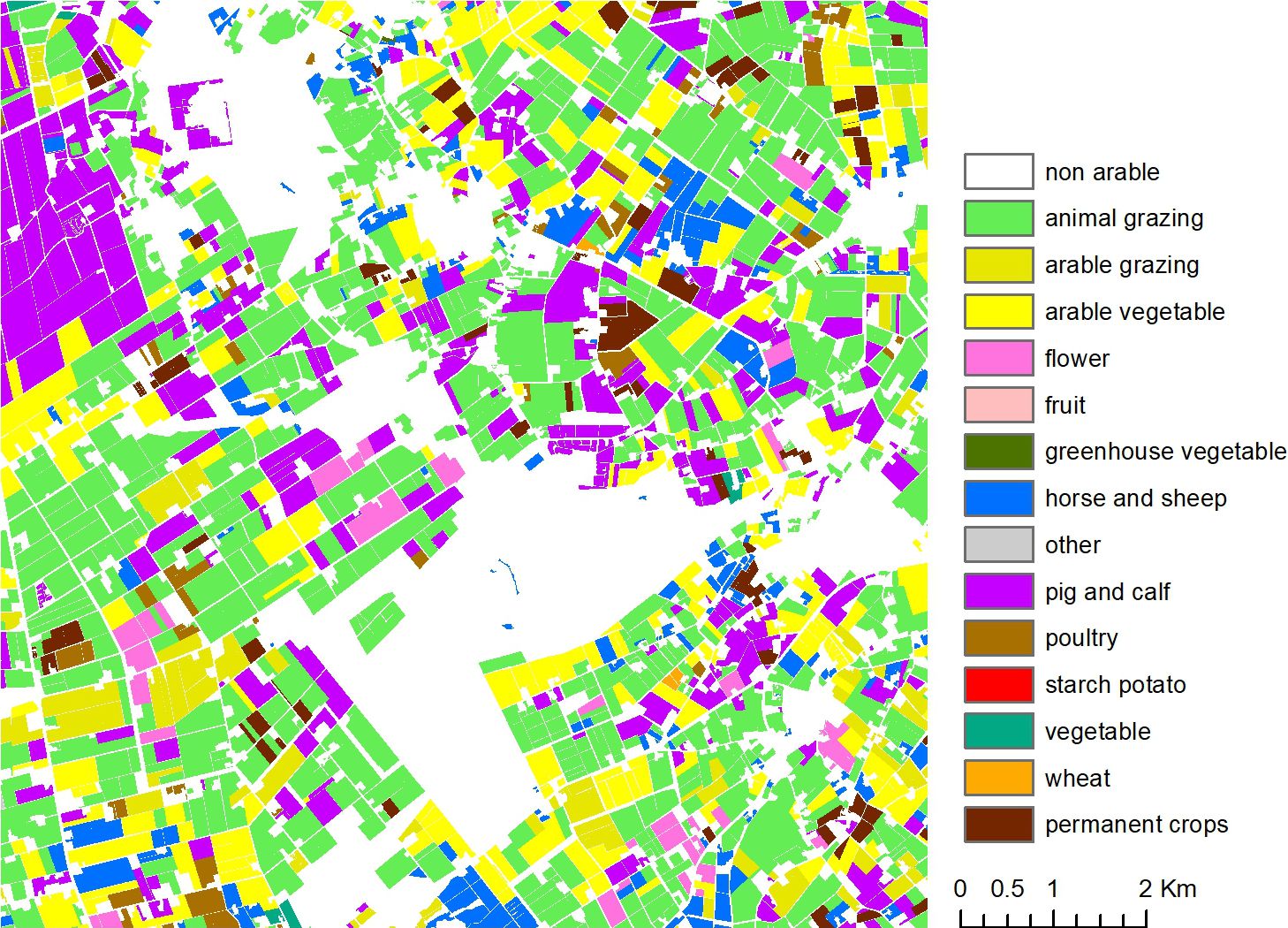
*

*Figure B9 Visual overview of study area 8. 10x10 km. mapping the basic elements of the landscape visible at this scale (A). and farm types according to classification presented in Appendix A. Table A4 (B)*

A)


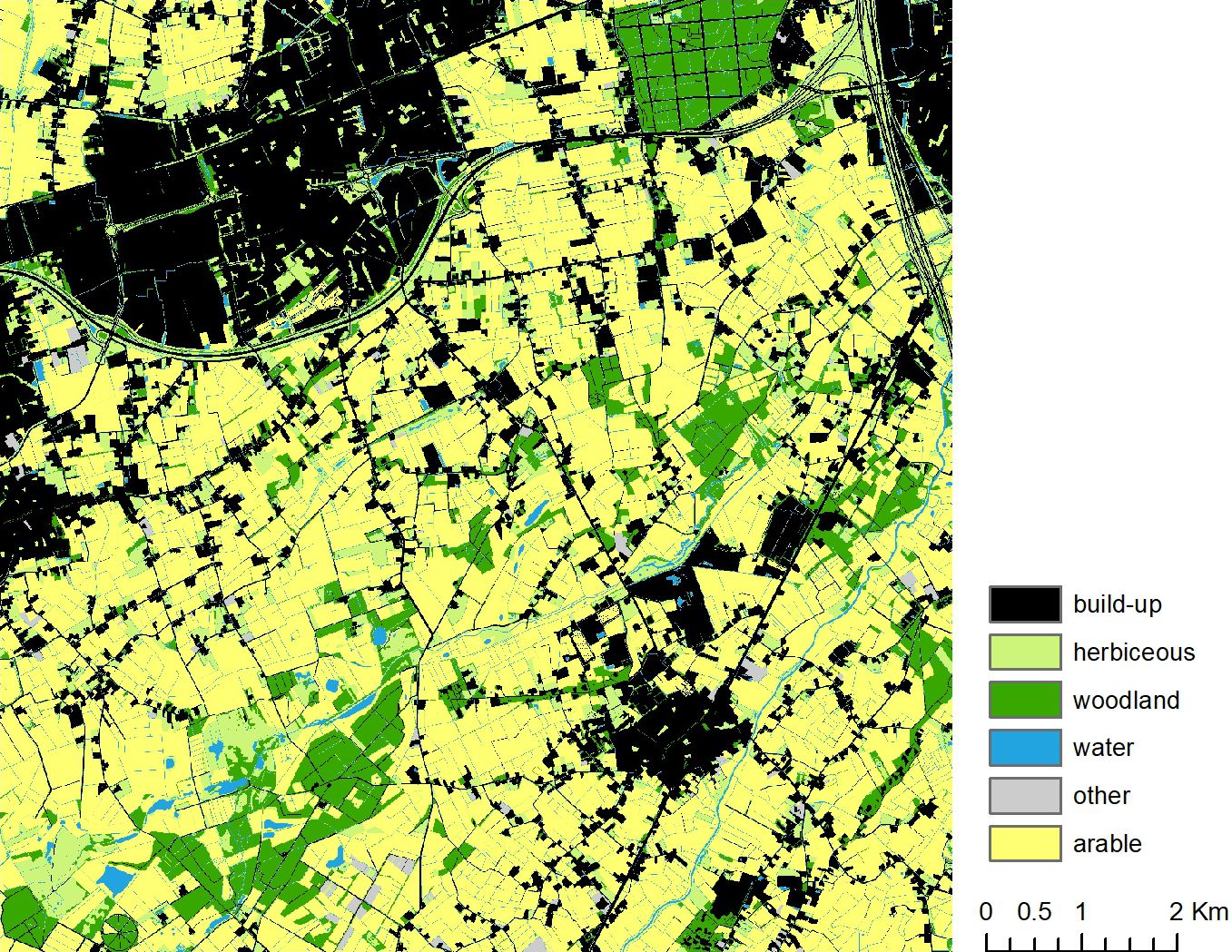


B)


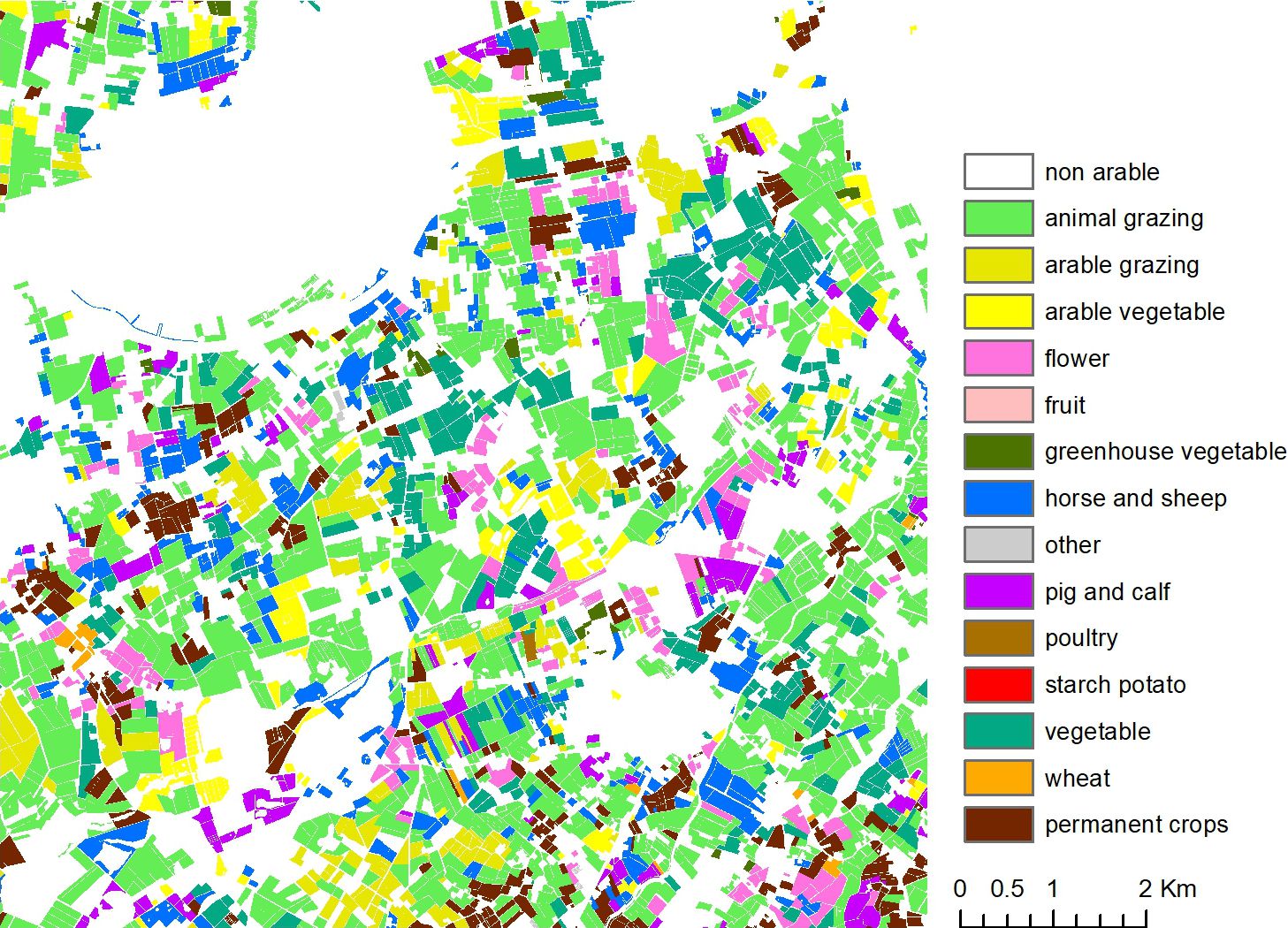


*Figure B10 Visual overview of study area 9. 10x10 km. mapping the basic elements of the landscape visible at this scale (A). and farm types according to classification presented in Appendix A. Table A4 (B)*

A)


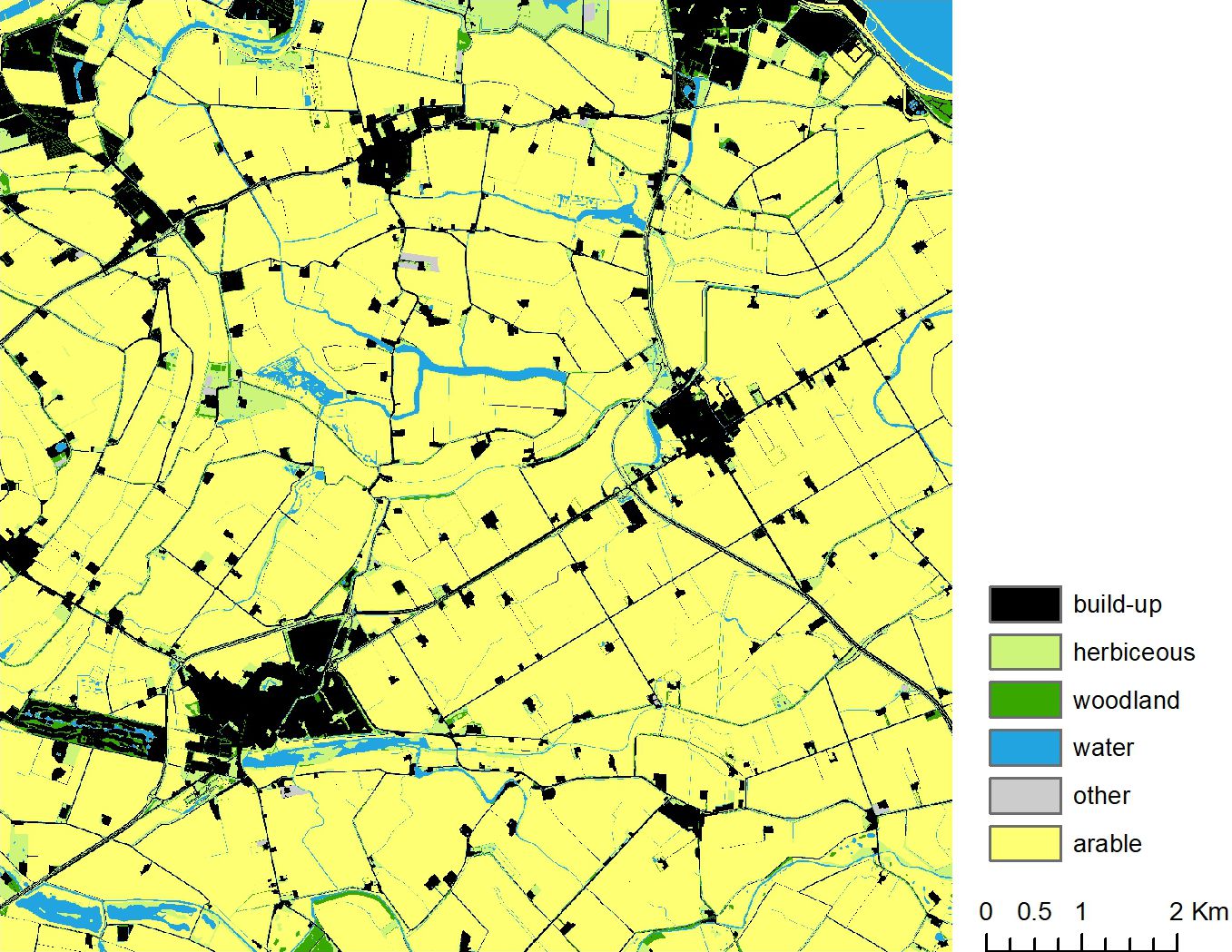


B)


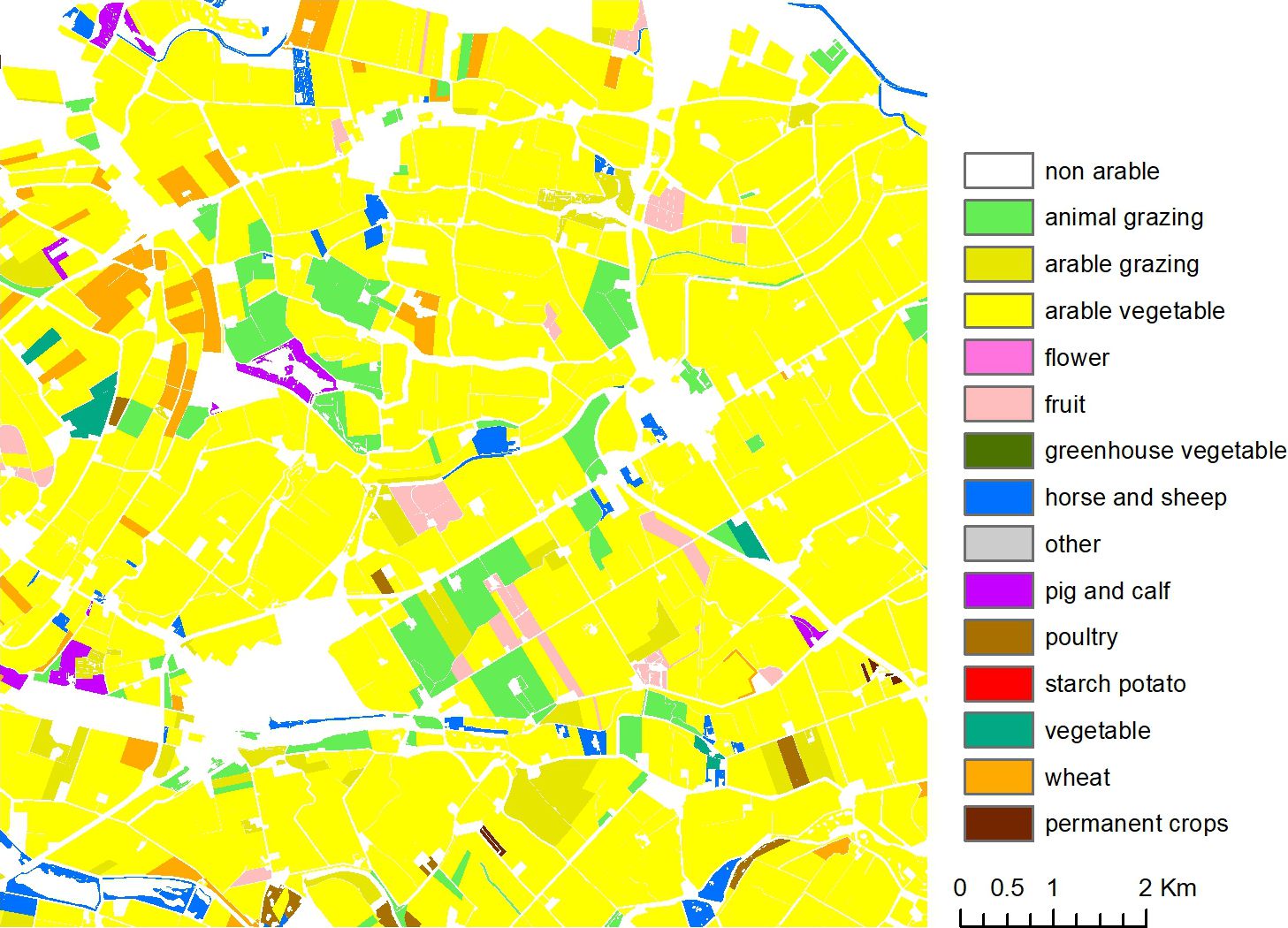


*Figure B11 Visual overview of study area 10. 10x10 km. mapping the basic elements of the landscape visible at this scale (A). and farm types according to classification presented in Appendix A. Table A4 (B)*
