# Supplementary material for: Is the effectiveness of policy-driven mitigation measures on carabid populations driven by landscape and farmland heterogeneity? Applying a modelling approach in the Dutch agroecosystems
Source: PLoS One. 2022 Dec 27;17(12):e0279639. doi: 10.1371/journal.pone.0279639 (PMC9794068; doi:10.1371/journal.pone.0279639)
Supplement: S4 Appendix — (DOCX) [file pone.0279639.s004.docx]

# Appendix D. Results – supplementary data

This appendix includes supplementary figures and tables for the Results section divided into the following sections: beetle populations in baseline ‘worst-case’ scenarios (1), the impacts on beetle populations in pesticide-related scenarios (2), landscape-related scenarios (3), and combined effects of both types of scenarios (4).

1. Beetle populations in baseline ‘worst-case’ scenarios

Table D1 Estimated regression parameters, standard errors, t‐values and P‐values for the regression models with lowest AIC for the mean overall beetle density in baseline ‘worst-case’ with DT50 of 25 days (A) and 1.3 days (B).

|  | **Estimate** | **Std. error** | ***t* value** | ***P*-value** |
| --- | --- | --- | --- | --- |
| 1. Model significant at *p* < 0.01, adjusted *R-squared* = 0.98 | | | | |
| (Intercept) | -2.89E+04 | 3.41E+04 | -0.846 | 0.436 |
| diversity_land | 3.12E+05 | 5.87E+04 | 5.311 | 0.003 |
| diversity_farm | -3.15E+04 | 2.63E+04 | -1.199 | 0.284 |
| fields_no | -3.30E+01 | 1.45E+01 | -2.284 | 0.071 |
| animal_farms_px | 7.66E-03 | 5.11E-04 | 14.995 | 0.000 |
| 1. Model significant at *p* < 0.01, adjusted *R-squared* = 0.97 | | | | |
| (Intercept) | -1.71E+04 | 3.54E+04 | -0.482 | 0.647 |
| diversity_land | 2.65E+05 | 5.26E+04 | 5.034 | 0.002 |
| fields_no | -3.14E+01 | 1.47E+01 | -2.133 | 0.077 |
| animal_farms_px | 7.67E-03 | 5.10E-04 | 15.021 | 0.000 |

Table D2 Estimated regression parameters, standard errors, t‐values and P‐values for the regression models with lowest AIC for the mean beetle abundance in baseline ‘worst-case’ with DT50 of 25 days (A) and 1.3 days (B).

|  | **Estimate** | **Std. error** | ***t* value** | ***P*-value** |
| --- | --- | --- | --- | --- |
| 1. Model significant at *p* < 0.01, adjusted *R-squared* = 0.97 | | | | |
| (Intercept) | 8.78E+00 | 7.35E-01 | 11.949 | 0.000 |
| LSI | 1.02E-02 | 6.73E-03 | 1.516 | 0.190 |
| diversity_land | 2.45E+00 | 1.29E+00 | 1.890 | 0.117 |
| fields_no | -9.20E-04 | 3.14E-04 | -2.928 | 0.033 |
| animal_farms_px | 1.47E-07 | 1.27E-08 | 11.575 | 0.000 |
| 1. Model significant at *p* < 0.01, adjusted *R-squared* = 0.97 | | | | |
| (Intercept) | 8.47E+00 | 7.19E-01 | 11.768 | 0.000 |
| LSI | 7.47E-03 | 6.59E-03 | 1.133 | 0.308 |
| diversity_land | 2.93E+00 | 1.27E+00 | 2.314 | 0.069 |
| fields_no | -8.48E-04 | 3.08E-04 | -2.756 | 0.040 |
| animal_farms_px | 1.50E-07 | 1.25E-08 | 12.012 | 0.000 |

Table D3 Estimated regression parameters, standard errors, t‐values and P‐values for the regression models with lowest AIC for the mean beetle occupancy in baseline ‘worst-case’ with DT50 of 25 days (A) and 1.3 days (B).

|  | **Estimate** | **Std. error** | ***t* value** | ***P*-value** |
| --- | --- | --- | --- | --- |
| 1. Model significant at *p* < 0.01, adjusted *R-squared* = 0.95 | | | | |
| (Intercept) | 1.79E+01 | 4.86E+00 | 3.686 | 0.008 |
| diversity_land | 2.89E+01 | 4.99E+00 | 5.782 | 0.001 |
| animal_farms_px | 6.04E-07 | 5.73E-08 | 10.55 | 0.000 |
| 1. Model significant at *p* < 0.01, adjusted *R-squared* = 0.96 | | | | |
| (Intercept) | 2.42E+01 | 4.60E+00 | 5.262 | 0.002 |
| LSI | -4.67E-02 | 4.01E-02 | -1.166 | 0.288 |
| diversity_land | 2.99E+01 | 5.18E+00 | 5.768 | 0.001 |
| animal_farms_px | 6.10E-07 | 5.94E-08 | 10.269 | 0.000 |

1. Decreasing insecticides’ toxicity

Table D4 Estimated regression parameters, standard errors, t‐values and P‐values for the regression models with lowest AIC for the change in mean overall beetle density in relation to the baseline ‘worst-case’ with DT50 of 25 days (A) and 1.3 days (B).

|  | **Estimate** | **Std. error** | ***t* value** | ***P*-value** |
| --- | --- | --- | --- | --- |
| 1. Model significant at *p* < 0.01, adjusted *R-squared* = 0.73 | | | | |
| (Intercept) | 3.30E+01 | 5.35E+00 | 6.176 | 0.000 |
| diversity_farm | -8.02E+00 | 3.80E+00 | -2.114 | 0.072 |
| animal_farms_px | -3.83E-07 | 8.19E-08 | -4.669 | 0.002 |
| 1. Model significant at *p* < 0.01, adjusted *R-squared* = 0.84 | | | | |
| (Intercept) | 4.56E+01 | 5.42E+00 | 8.424 | 0.000 |
| diversity_farm | -1.16E+01 | 3.85E+00 | -3.010 | 0.020 |
| animal_farms_px | -5.18E-07 | 8.30E-08 | -6.245 | 0.000 |

Table D5 Estimated regression parameters, standard errors, t‐values and P‐values for the regression models with lowest AIC for the change in mean beetle abundance in relation to the baseline ‘worst-case’ with DT50 of 25 days (A) and 1.3 days (B).

|  | **Estimate** | **Std. error** | ***t* value** | ***P*-value** |
| --- | --- | --- | --- | --- |
| 1. Model significant at *p* = 0.02, adjusted *R-squared* = 0.45 | | | | |
| (Intercept) | -3.281 | 1.936 | -1.695 | 0.129 |
| fields_no | 0.002 | 0.001 | 2.880 | 0.021 |
| 1. Model significant at *p* = 0.16, adjusted *R-squared* = 0.24 | | | | |
| (Intercept) | -1.05E-01 | 2.81E+00 | -0.037 | 0.971 |
| fields_no | 2.89E-03 | 1.34E-03 | 2.155 | 0.068 |
| animal_farms_px | -1.07E-07 | 6.54E-08 | -1.635 | 0.146 |

Table D6 Estimated regression parameters, standard errors, t‐values and P‐values for the regression models with lowest AIC for the change in mean beetle occupancy in relation to the baseline ‘worst-case’ with DT50 of 25 days (A) and 1.3 days (B).

|  | **Estimate** | **Std. error** | ***t* value** | ***P*-value** |
| --- | --- | --- | --- | --- |
| 1. Model significant at *p* < 0.01, adjusted *R-squared* = 0.92 | | | | |
| (Intercept) | 3.79E+01 | 3.69E+00 | 10.255 | 0.000 |
| diversity_land | -9.86E+00 | 5.61E+00 | -1.758 | 0.129 |
| diversity_farm | -7.01E+00 | 2.93E+00 | -2.397 | 0.054 |
| animal_farms_px | -3.56E-07 | 4.44E-08 | -8.021 | 0.000 |
| 1. Model significant at *p* < 0.01, adjusted *R-squared* = 0.97 | | | | |
| (Intercept) | 4.61E+01 | 2.86E+00 | 16.094 | 0.000 |
| diversity_land | -1.55E+01 | 4.35E+00 | -3.559 | 0.012 |
| diversity_farm | -6.48E+00 | 2.27E+00 | -2.855 | 0.029 |
| animal_farms_px | -4.07E-07 | 3.44E-08 | -11.826 | 0.000 |

1. Introducing field margins

Table D7 Estimated regression parameters, standard errors, t‐values and P‐values for the regression models with lowest AIC for the change in mean overall beetle density in relation to the baseline ‘worst-case’ with DT50 of 25 days (A) and 1.3 days (B).

|  | **Estimate** | **Std. error** | ***t* value** | ***P*-value** |
| --- | --- | --- | --- | --- |
| 1. Model significant at *p* = 0.02, adjusted *R-squared* = 0.79 | | | | |
| (Intercept) | 1.72E+01 | 2.44E+00 | 7.056 | 0.001 |
| LSI | -3.89E-02 | 2.36E-02 | -1.649 | 0.160 |
| diversity_farm | -4.05E+00 | 2.03E+00 | -1.996 | 0.102 |
| fields_no | 1.71E-03 | 9.66E-04 | 1.770 | 0.137 |
| animal_farms_px | -1.55E-07 | 4.74E-08 | -3.265 | 0.022 |
| 1. Model significant at *p* = 0.01, adjusted *R-squared* = 0.74 | | | | |
| (Intercept) | 1.57E+01 | 2.23E+00 | 7.052 | 0.000 |
| diversity_farm | -6.14E+00 | 2.04E+00 | -3.003 | 0.024 |
| fields_no | 2.07E-03 | 1.10E-03 | 1.884 | 0.109 |
| animal_farms_px | -2.07E-07 | 4.54E-08 | -4.566 | 0.004 |

Table D8 Estimated regression parameters, standard errors, t‐values and P‐values for the regression models with lowest AIC for the change in mean beetle abundance in relation to the baseline ‘worst-case’ with DT50 of 25 days (A) and 1.3 days (B).

|  | **Estimate** | **Std. error** | ***t* value** | ***P*-value** |
| --- | --- | --- | --- | --- |
| 1. Model significant at *p* = 0.05, adjusted *R-squared* = 0.55 | | | | |
| (Intercept) | 2.15E+00 | 7.16E-01 | 3.003 | 0.024 |
| LSI | -2.14E-02 | 6.24E-03 | -3.426 | 0.014 |
| diversity_land | 2.20E+00 | 8.08E-01 | 2.717 | 0.035 |
| animal_farms_px | 2.30E-08 | 9.25E-09 | 2.485 | 0.048 |
| 1. Model significant at *p* = 0.08, adjusted *R-squared* = 0.47 | | | | |
| (Intercept) | 1.52E+00 | 9.97E-01 | 1.525 | 0.178 |
| LSI | -2.42E-02 | 8.69E-03 | -2.785 | 0.032 |
| diversity_land | 3.01E+00 | 1.13E+00 | 2.674 | 0.037 |
| animal_farms_px | 2.84E-08 | 1.29E-08 | 2.204 | 0.070 |

Table D9 Estimated regression parameters, standard errors, t‐values and P‐values for the regression models with lowest AIC for the change in mean beetle occupancy in relation to the baseline ‘worst-case’ with DT50 of 25 days (A) and 1.3 days (B).

|  | **Estimate** | **Std. error** | ***t* value** | ***P*-value** |
| --- | --- | --- | --- | --- |
| 1. Model significant at *p* = 0.02, adjusted *R-squared* = 0.77 | | | | |
| (Intercept) | 1.31E+01 | 2.34E+00 | 5.620 | 0.002 |
| diversity_land | -5.18E+00 | 4.02E+00 | -1.287 | 0.254 |
| diversity_farm | -3.15E+00 | 1.80E+00 | -1.752 | 0.140 |
| fields_no | 2.06E-03 | 9.90E-04 | 2.085 | 0.092 |
| animal_farms_px | -1.73E-07 | 3.50E-08 | -4.944 | 0.004 |
| 1. Model significant at *p* < 0.01, adjusted *R-squared* = 0.88 | | | | |
| (Intercept) | 1.45E+01 | 1.77E+00 | 8.197 | 0.000 |
| diversity_land | -5.83E+00 | 3.04E+00 | -1.917 | 0.113 |
| diversity_farm | -3.12E+00 | 1.36E+00 | -2.288 | 0.071 |
| fields_no | 1.96E-03 | 7.49E-04 | 2.618 | 0.047 |
|  |  |  |  |  |
