# Supplementary material for: Is the effectiveness of policy-driven mitigation measures on carabid populations driven by landscape and farmland heterogeneity? Applying a modelling approach in the Dutch agroecosystems
Source: PLoS One. 2022 Dec 27;17(12):e0279639. doi: 10.1371/journal.pone.0279639 (PMC9794068; doi:10.1371/journal.pone.0279639)
Supplement: Attachment — Submitted filename: Carabid model manuscript.pdf [file pone.0279639.s007.pdf]

Manuscript

Click here to access/download;Manuscript;Manuscript.docx

Title: Is the effectiveness of policy-driven mitigation measures on carabid populations driven by landscape and farmland heterogeneity? Applying modelling approach in the Dutch agroecosystems

Short title: Landscape drives response of carabid populations to policy-driven mitigation measures

Elżbieta Ziółkowska[1*], Aaldrik Tiktak[2], Christopher J. Topping[3]

[1] Jagiellonian University in Kraków, Institute of Environmental Sciences, Gronostajowa 7, 30-387, Kraków, Poland

[2] PBL Netherlands Environmental Assessment Agency, Department of Water, Agriculture and Food, Bezuidenhoutseweg 30, 2594 AV The Hague, The Netherlands

[3] Department of Ecoscience – Biodiversity and Conservation, Århus University, C.F. Møllers Alle 8, 8000 Aarhus C, Denmark

Corresponding author: Elżbieta Ziółkowska

e-mail: e.ziolkowska@uj.edu.pl

Abstract

The growing challenges of protecting biodiversity in agro-ecosystems and maintaining high agricultural productivity has become an important issue within the European Union, shaping both European and national agro-policies. Presented study is part of a broader evaluation of the interim targets of the 2013 Dutch policy plan on sustainable use of pesticides, carried out in 2019 by the PBL Netherlands Environmental Assessment Agency. We aimed to assess the effectiveness of selected mitigation measures suggested in the policy plan on non-target terrestrial arthropods using common carabid beetle *Bemdidion lampros* as a model species. We combined dynamic landscape models with detailed agent-based population modelling to simulate impacts of reduction of toxicity of insecticides, reduction of spray drift to the off-crop area, and increase in area of field margins on the beetle population dynamics in ten agricultural landscapes representing different farming systems. Our simulations showed that shift towards low-risk products should be the priority if the goal is to increase beetle range, but to promote local beetle abundance this needs be coupled with increasing amount of field margins in a landscape. Overall, the observed treatment and landscape effects were highly context-specific and therefore we suggest that care is used when defining and interpretting metrics based on population effects of policy measures. This caveat notwithstanding, the use of simulation to assess complex interactions between landscape, ecology and behaviour of species, and policy measures can be a powerful tool supporting innovative policy management. This should include the development of landscape-context specific targets and/or mitigation measures.

Keywords

ALMaSS, agent-based modelling, agro-policy, dynamic landscape model, non-target arthropods, population dynamics

1. Introduction

There is an increased focus within European Union (EU) policies and strategies on protection of biodiversity whilst maintaining agricultural sustainability. Recently, the Green Deal sets targets for reduction in pesticide use by 50% by 2030. Formulated in the Farm to Fork and Biodiversity strategies, this reduction, together with other goals such as having 10% of the utilized agricultural area (UAA) reserved for wildlife, have their roots in the alarming pattern of decrease in abundance, biomass and range of insects in Europe [1–5] and worldwide [6,7]. Trends, although differing with taxa and regions [8], indicate that the most severe declines occur in landscapes dominated by agriculture [9–13].

Insect declines in agricultural landscapes are mainly driven by land-use changes associated with the transition from natural to agricultural lands and intensification of agricultural practices including land consolidation and increase in pollution by pesticides and fertilizers [6–8,14]. At the same time, maintaining high species diversity and population abundance within agricultural landscapes is crucial for many ecosystem services. Many of these functions are related directly to crop production, such as natural (biological) pest control and pollination [15,16]. Consequently protecting biodiversity in agro-ecosystems has become an important issue at the EU level, e.g. the EU Biodiversity Strategy for 2030 focuses on halting biodiversity loss and building healthy and environmentally friendly food systems in Europe. This and the Common Agricultural Policy (CAP) provide a general framework, but more and more it is up to member states to implement this framework in their specific contexts. Hence, protection of biodiversity in agricultural landscapes is reflected in national agro-policies, as well as agri-environmental programs. One such example, although pre-dating the Green Deal, is the policy document 'Healthy Growth, Sustainable Harvest' [17], drawn up by the Dutch government. This document sets up a more sustainable crop protection policy in the Netherlands for the 2013-2023 period. The aim is to achieve a sustainable use of

pesticides by reducing the risks and impacts of pesticide use on human health and the environment through promoting the use of Integrated Pest Management (IPM). At the same time, the ambition of this policy plan is to 'strengthen the economic prospects for Dutch agriculture and horticulture'.

In 2019, the policy interim targets related to integrated pest management, ecological water quality, drinking water quality, biodiversity, occupational safety, and food safety were evaluated by the PBL Netherlands Environmental Assessment Agency [18]. In terms of biodiversity, the interim target for 2018 was to reduce risks posed by chemical plant protection products to non-target arthropods by reducing the use of hazardous substances in exchange for low-risk products and non-chemical measures, and creation of habitats (refuges) for pollinators and pest control species. The PBL interim evaluation concluded that this target, among others, has not been achieved and, moreover, the situation has slightly deteriorated between 2013 and 2018 [18]. The high intensity of farming in the Netherlands may be blamed for this situation. The sale of plant-protection products per hectare of UAA is still very high in the Netherlands compared to other EU Member States. According to Eurostat statistics, in 2018 the Netherlands was in third place in terms of both plant-protection products sale (with approximately 5 kg/ha of UAA) and insecticides sale (with approximately 0.9 kg/ha of UAA). In addition, no substantial shift towards 'low-risk' substances has been observed, and the sale of insecticides (which account for the largest share of environmental burden) has not decreased in recent years [19]. The pesticide-related environmental risk is, however, not equally distributed across arable land, but rather largely depends on dominant farm type, being highest in areas where intensively sprayed crop and plant species, such as potatoes, onions, lilies and tulips are grown [18].

Both spatial distribution of crop and non-crop habitats and management of crop habitats (including applications of pesticides) drive source-sink dynamics and performance of non-

target organisms in complex agricultural landscapes. To secure crucial agro-ecosystem functions, a broader, system-based perspective is needed which considers large-scale effects of plant protection products [20]. Coupling landscape models with agent-based population modelling and detailed knowledge on ecology and ecotoxicology allows simulation and better understanding of population-level impacts of pesticide applications [21,22]. These techniques can be also use for conducting in silico experiments to test management implications and to answer policy questions regarding biodiversity protection in agricultural landscapes [23–26].

The aim of this study was to evaluate the effectiveness of selected mitigation measures from the Dutch policy document 'Healthy Growth, Sustainable Harvest' on non-target terrestrial arthropod, ground beetle *Bembidion lampros*, using combination of spatial analysis and simulation techniques. Mitigation measures selected included: (1) substitution of harmful pesticides by less harmful pesticides (simulating shift to low-risk products), (2) reducing spray drift emission to the off-crop area (part of the Dutch regulation), and (3) introducing grassy field margins to a percentage of field parcels within a landscape (a common mitigation strategy). The first two measures aim to reduce the negative impacts of applying pesticides, while the third is related to landscape management with objective of providing additional habitats for pest control agents and pollinators.

We used the spatially explicit agent-based Animal, Landscape and Man Simulation System (ALMaSS) [27] to answer the following questions: (1) what drives beetle populations in diverse agricultural landscapes of the Netherlands, (2) which mitigation measure supports beetle at best, and (3) to what extent impacts of mitigation measures are modified by the landscape context. This third point is important because if landscape structure alters the utility of a policy instrument, then it might suggest that even the national-level tailoring of EU regulation may be operating at too large scale.

2. Methods

The modelling environment ALMaSS is an open-source project hosted on GitLab (https://gitlab.com/ALMaSS/) with online documentation (https://projects.au.dk/almass/documentation/) using the ODdox (Overview Design doxygen) protocol [28]. The ALMaSS itself is a large system comprised of two main components, animal representations and landscape model designed to service the simulated animal. As modelled species we used the carabid beetle *B. lampros*, representing a large, diverse, and important group of ecosystem providers, responsible for natural pest control [29,30] and weed control [31,32] in agro-ecosystems. *B. lampros* was specifically chosen as a representant of field-edge polyphagous predators widespread in the agricultural landscapes of the Netherlands [33]. In addition, the well-documented agent-based model of *B. lampros* already existed in the ALMaSS modelling framework [34].

Before carrying out simulations in ALMaSS, the specific context of the Dutch agricultural system needed to be incorporated into the modelling framework. Therefore, we divided the methods into three sections describing (1) the design of landscape model in ALMaSS and its parametrization for the Dutch landscapes; (2) the details of species model used; and (3) scenarios design and analysis for evaluating mitigation measures and landscape effects.

2.1 Parametrization of the ALMaSS landscape model for the Dutch landscapes

2.1.1 General landscape model

Highly dynamic and heterogenous character of agro-ecosystems necessitates a detailed, spatio-temporal landscape representation to provide a realistic environment for the modelled species. In ALMaSS, the spatial landscape heterogeneity is described by a detailed raster land cover map with complete coverage and spatial resolution of 1 m^2. Such level of accuracy is appropriate for insect species, like *Bembidion*, that utilize narrow habitats such as field

margins. Each unit in the raster land cover map is classified in accordance with its landscape element type (e.g., natural or permanent grassland, field in rotation, built-up area), including detailed structures important for the species under consideration, such as hedgerows or field margins. Field boundaries are delineated, with each field belonging to a given farm unit (managed by the same farmer). Farm units are classified into different types, e.g., cattle, pig or arable farms, based on structure of crops grown and animals present on the farm.

The temporal landscape heterogeneity in ALMaSS includes both crop management throughout a year, described through individually tailored management plans for each crop, and the cropping system understood as a pluriannual crop rotation. Associated vegetation growth models for all modelled vegetation types and crops supply vegetation height, green and total biomass on a daily basis, and are fed by weather conditions (mean daily temperature, mean daily wind speed and daily sum of precipitation). Crop management plans consist of logical and ordered combinations of farm activities (related to soil cultivation, and application of fertilizers and pesticides), as well as time windows and probabilities of carrying out activities. These conditions may relate to weather, crop growth, soil type or previous farming activities. Pluriannual crop rotations are defined separately for each farm type based on the proportion of each cultivated crop or group of crops assumed to be grown, and associated agronomic constraints (e.g. for disease prevention, or logistics). Such an approach gives a detailed dynamic landscape model with vegetation growing on a daily basis in response to the weather, and the pattern of farming activities related to each specific crop, farm, and field [27,35].

2.1.2 'Capturing' the Dutch agricultural system *in silico*

All details related to the generation of the landscape model for the Netherlands are described in Appendix A with an overview given here. This approach broadly followed [35] but required specific Dutch conditions and datasets to be taken into account.

Four main types of data were gathered and processed to obtain the necessary ALMaSS landscape inputs:

i. *Land cover / land use information*. The data were derived from the digital object-oriented topographic database of the Netherlands (TOP10NL) with level of details corresponding with topographic maps at the scale of 1:10 000 (available for browsing at https://www.pdok.nl/viewer/). We used nine classes of objects from the TOP10NL database to map 45 different layers of spatial information. Individual layers of land use / land cover information together with information on agricultural fields derived from the Agricultural Area Netherlands (Agrarisch Areaal Nederland) were then combined into a single raster landscape map in a step-by-step process.

ii. *The Land Parcel Identification System (LPIS)*. LPIS records information on all agriculturally managed reference parcels (geographically delimited areas with unique identification codes) in the EU Member States, and serves as a controlling mechanism under the CAP. In the Netherlands, LPIS is managed by the Netherlands Enterprise Agency (Rijksdienst voor Ondernemend Nederland). Information on type of crops cultivated in reference parcels, ID numbers of agricultural holdings enabling the grouping of individual reference parcels into farm units, as well as farm types were obtained for 2015 from the agricultural register 'Basisregistratie Percelen'. View and download services for the most recent agricultural register are available at https://data.overheid.nl/.

The original farm classification from the Netherlands Enterprise Agency including 38 different farm types was simplified (based on cluster analysis taking into account similarities in acreage of different crop types, processed in R 3.4.2) to 15 farm types: animal grazing, arable grazing, arable vegetable, vegetable, greenhouse vegetable,

as part of this study?

wheat, starch potato, fruit, flower, poultry, pig and calf, horse and sheep, permanent crop, vineyards and other farms (Appendix A, Table A4).

iii. *Up-to-date crop management plans*. These plans for dominant Dutch crops (winter wheat, spring barley, maize, beet, cabbage, carrot, potatoes, tulips, and managed grassland – permanent and in rotation with other crops) including time windows and probabilities of occurrence of main soil cultivation practices, as well as fertilizer and pesticide applications (together with information about the product used and its dosage) were obtained from farmer advisors from the Delphy company. To allow ALMaSS landscape simulator to modify soil cultivation depending on soil type, dominant soil type for each field parcel was defined based on the map of soil types (Grondsoortenkaart) derived from the soil map of 1:50,000 (view and download services available at https://data.overheid.nl/).

Based on proportions of crops cultivated by farms of different types, crop rotation schemes were prepared for each farm type individually (Appendix A, Table A5). The crop rotation scheme consists of 100 crop entries with multiple entries of each crop type (1 crop for each 1% by area). The order of crops followed typical agronomic practices and issues such as late harvest leading to impossible sowing conditions were controlled by the built-in ALMaSS farm code. In addition, catch crops (i.e., crops grown between successive plantings of main crops) were included in the rotations following the specific rules defined by the farmer advisors from Delphy. At the start of each simulation, a random crop in the rotation is taken as the starting point for each arable field in a given farm and the next crop in the list is assumed to be grown in the same field in the following year. After 100 simulation years, all fields of one farm type would have raised each of the 100 crops in the rotation list once on each field, but in a different order. If a specific crop, e.g., maize for silage, occurs 13 times

out of 100 in the rotation, it will on average occur on 13% of all fields covered by that rotation at any point in time.

All handling and analysis of spatial data were done using Python 2.7 and the Python library arcpy to access ArcGIS features, or directly in ArcGIS 10.4 [36]. The entire process of producing Dutch landscape models for ALMaSS has been programmed in Python and R scripts.

2.1.3 Representative study areas

As all procedures for generating Dutch landscape models for ALMaSS are automated or semi-automated, any landscape in the Netherlands can be 'captured' and used for simulation. However, since running the CPU intensive simulations for the entire country is not feasible, we selected 10 study areas of 10 x 10 km^2 representing a gradient from heterogeneous (small-scale) to rather homogeneous landscapes with different farm management and soil properties (Fig. 1; see Appendix B for a visual overview of study areas).

Structural and farmland heterogeneity of selected study areas were described through a set of landscape- and class-level metrics calculated at 1-m resolution using the software package FRAGSTATS v4 [37] (Tab. 1, Tab. B1 in Appendix B). While some study areas were more fine-grained structured with a high coverage of semi-natural habitats (> 15%), high number of small fields (and therefore high field margins density) and high farming diversity (study areas 7, 8 and 9; Fig. 2), others exhibited little structural and/or farming diversity, being dominated by large fields (mean field size > 4 ha) managed in a similar way (study areas 4 and 5 situated in the Dutch polder regions with predominantly arable farming; Fig. 1 and Fig. 2).

2.2 Animal model

The original ALMaSS model for the *B. lampros* was described in [34]. Due to the high real world beetle density, the model uses the super-individual concept, which means that each

beetle agent in the model represents 100 real world beetles [22]. *Bembidion* behaviour is simulated on a daily time-step. All individuals are categorized as being members of four life-stages: egg, larvae, pupae, and adult female (males are not modelled as they do not limit the population size). Behaviour is characterized by annual dispersal and aggregation phases with aggregation linked to non-cultivated habitats and dispersal and breeding largely occurring in open areas. Primary drivers in the model are temperature-controlled developmental rates of eggs, larvae and pupae, together with adult female interactions with the landscape. At high densities species model invoke density dependence via intraspecific predation. Beetle mortality values for different crop management operations were defined based on [38].

The response to the pesticide is included in the model by assuming a threshold environmental concentration above which there is a daily probability of mortality (*p*). This probability is calculated from the following equation:

$$(1-m) = (1-p)^d, \qquad [Eq.\ 1]$$

where *m* is the proportion of beetles assumed to die (e.g. 0.9 for 90% mortality over the test period) and *d* is the number of days over which the test was carried out. If the beetle finds itself in a 1-m^2 grid cell with an environmental concentration above the trigger, then it is assumed to die with the probability *p*. There is no dose–response, so the maximum death rate is set as *m* over *d* days.

2.3 Simulation scenarios

We implemented the following mitigation measures in ALMaSS (summary provided in Figure 3): (i) substitution of harmful pesticides by less harmful pesticides; (ii) reduction of spray drift emission to the off-crop area; and (iii) introduction of grassy field margins to a certain percentage of fields within a landscape. Measure (i) aims to provide a direct reduction in emission and/or impact of pesticides on the fields, while measure (ii) and (iii) are used to

reduce the emission to off-crop areas, such as field ditches. In addition, measure (iii) provides additional overwintering, reproduction or foraging habitats for modelled species.

2.3.1 Pesticide-related mitigation measures

Toxicity: We used realistic pesticide application scenarios provided by extension advisors (see chapter 2.1.2). However, to reduce computation time, we classified the pesticides into three groups based on toxicity instead of values related to specific active ingredients and/or products to account for broad diversity of products used in the Netherlands. For insecticides, we assumed high, medium or low toxicity, and for normal fungicide and herbicide application we assumed no impact on the carabid beetles. We defined the high toxicity level of insecticides as related to beetle field lethality rate of 90% (LR90) measured for a foliar insecticide-spray application over seven days, which gives daily probability of mortality p of 0.28. For a subset of scenarios, decreased field lethality to medium (LR50 corresponding with $p = 0.09$) or low (LR25 corresponding with $p=0.04$) level was assumed; Fig. 3), simulating e.g. decreased sensitivity of the beetles, or equivalent decreased concentration or less toxic insecticides.

Environmental decay: In simulations, we considered two significantly different values for environmental degradation time (DT_{50}) in 20°C: 1.3 days and 25 days (Fig. 3). These values represent two different groups of insecticides with high and low environmental degradation properties and were chosen based on measured DT_{50} values of insecticides most commonly used in the Netherlands. The temperature dependence of DT_{50} value was defined according to the following equation [39]:

$$DT50(t) = DT50(20°) \times exp[0.094779 \times (20°-t)] \qquad [Eq. 2]$$

where t is a given mean daily temperature.

To allow direct comparisons of the toxicity-levels applied when considering different DT_{50}, a toxic unit approach was used. To ensure that beetles could be exposed above the trigger

threshold for at least the period defined by DT_{50} (1.3 or 25 days), a treatment rate of 41.78 of the trigger concentration at each LR was used for insecticides with DT_{50} = 1.3 days, and a treatment rate of 1.22 of the trigger concentration at each LR was used for insecticides with DT_{50} = 25 days. These rates (41.78 and 1.22) were calculated as follows:

$$treatment\ rate = 1\ /\ [0.5^{1/DT50\ [days]} \times duration\ of\ effect\ [days]] \qquad [Eq.\ 3]$$

This means that for low DT_{50} the probability of dying if exposed in the first day is very high whereas for high DT_{50} the daily chance is lower but effect period longer.

Spray drift: During the application of pesticides, part of the spray liquid may be carried out of the treated area by wind or the air stream of the air-blast sprayer and may be further spread by drift and result in a contamination of adjacent crops and waters. In all simulations we assumed that drift occurs up to 12 m from the edge of any sprayed field, following the equation by Rautmann et al. [40]:

$$\%\ drift = A \times dist^{B} \qquad [Eq.\ 4]$$

where A and B are coefficients that depend on crop and *dist* is the distance between the field border and a point downwind the field (m). For field crops assuming downward spraying, and no drift reducing technologies, A = 2.7705 and B = -0.9787 [40]. In the Netherlands, farmers must reduce the spray drift by at least 50%, and the newest requirements (from January 1, 2018) refer to 75%. However, a survey amongst 600 farmers showed that 70% of Dutch farmers reduced the spray drift by 90% [18,41]. This was partly voluntarily, but mandatory when they used toxic pesticides for which there is an additional restriction to reduce the spray drift by at least 90%. In our study, we used two scenario's for spray drift, i.e. the baseline with 50% spray drift reduction and a scenario in which all farmers reduce the spray drift by 90%. The latter is the aim of the new policy document "Vision on crop protection 2030" [42]. We re-calculated the coefficients of [Eq. 4], for 50% and 90% drift reduction and tested their influence in a subset of scenarios for the ground beetles (Fig. 3).

2.3.2 Landscape-related mitigation measures

According to Dutch legal requirements, a crop-free and spray-free buffer zone is to be maintained alongside surface water. The width of the buffer zone ranges from 0.5 m to 1.5 m, depending on crop type. Because the resolution of ALMaSS is 1 m, the crop-free buffer zone was set to 1 m for all crops. We assumed that these crop-free zones are fallow at the beginning of each simulation year with grass re-growing during the year.

To test different landscape management strategies land-cover map of each study area was used in two artificially manipulated forms. The first included fields that were completely covered by the crop, the second was to create a field boundary in-field around the crops (not subjected to the same agricultural practice as the crop itself such as ploughing or harvesting or pesticide application). Grassy boundaries of 4-m width were applied to 50% of fields in each study area, in a subset of scenarios (Fig. 3).

2.3.3 Simulation set-up

The simulation for each combination (16 combinations of scenarios, see Fig. 3) was replicated 10 times, as this number of replicates was shown to be sufficient to account for between-replicates variability, which in case of the *Bembidion* model is generally very low [22]. Initial conditions for each replicate differed in the distribution of beetles across the study area and the initial allocation of crops in the fields, but not in the starting number of super-individuals which was set to 200 000 per 100 km^2. All simulations were run for 30 simulation years with no burn-in period.

Weather conditions were selected to represent the 1998–2017 period from De Bilt climatic station, which is considered representative for Dutch conditions (data from European Climate Assessment & Dataset; www.ecad.eu).

2.3.4 Simulation data extraction and analysis

From each simulation, three endpoints were analysed: overall beetle population density (i.e., total number of adult female beetles divided by the landscape area, i.e. 100 km^2), Occupancy (i.e., beetle distribution defined as proportion of grid cells in the landscape with at least 100 adult female beetles) and Abundance (mean density of adult females in the occupied areas). The latter two endpoints are presented as Abundance-Occupancy Relationship (AOR) plots [43]. Although the spatial resolution of landscape model in ALMaSS is 1 m^2, for Occupancy and Abundance calculation grid cells of 50 m^2 were used. Overall beetle population density, and beetle Abundance and Occupancy were measured at day 59 of each year (March 1st) of each simulation, and then means over 30 years of the simulations were calculated. These endpoints were then averaged across 10 replicate runs for each scenario. To determine the effect size of each mitigation measure on *B. lampros* population, the impact of each scenario relative to the baseline was used and compared over time. To highlight improvements related to the treatments, we set baseline conditions according to the 'worst-case' scenario with high insecticide toxicity (LR90), insecticide drift reduction of 50% and no additional field margins added to the landscape (FM_OFF). Two such baselines were created, one for low and high (1.3 and 25 days) environmental half-life, respectively.

The influence of metrics describing landscape and farmland heterogeneity (Tab. 1) on mean overall beetle density, abundance and occupancy in the baseline ('worst-case') scenarios A and B, and on changes of mean overall beetle density, abundance and occupancy in response to applied mitigation measures was tested with multiple regression models. Metrics of landscape and farmland heterogeneity were first checked for correlations and those with Pearson correlation coefficient > |0.7| were removed from the analysis. As a result, the following metrics were used and explanatory variables in the regression models: landscape shape index, landscape diversity, farming diversity, percentage of animals farms and number of fields in a landscape. Separate multiple regression models were constructed for pesticide-

related and landscape-related mitigation measures, for each of the endpoints considered. The best performing models were chosen with backward stepwise algorithm based on the Akaike information criterion. For each model, we reported adjusted *R-squared* value and *p*-value. Models and variables with p-value ≤ 0.05 were considered significant. Calculations were performed in R environment [44].

3. Results

This section first describes the beetle populations simulated under the baseline scenarios. Then, the effectiveness of different mitigation measures on the population of *B. lampros* in 10 study areas is presented.

3.1 Beetle populations under the baseline scenarios

The beetle populations under the baseline scenarios (LR90 combined with spray drift reduction of 50%, and no additional field margins) varied across study areas (Fig. 4). Beetle populations were slightly lower when a pesticide with a longer half-life was applied, although the difference is very small (Fig. 4). The mean overall beetle density and Occupancy in the baseline scenario A increased with the landscape diversity ($p < 0.01$) and percentage of animal farms in a landscape (i.e., with the amount of managed grasslands; $p < 0.01$; Table C1 and C3 in Appendix C). Mean beetle Abundance was influenced only by farmland heterogeneity. It increased with the area of animal farms in a landscape ($p < 0.01$), but decreased with increasing number of fields (and decreasing field size) in a landscape; $p = 0.03$; Table C2 in Appendix C). Similar trends were observed when the DT_{50} was decreased to 1.3 day (baseline scenario B; see Tables C1-C3 in Appendix C).

3.2 Effects of mitigation measures on beetles

The impacts of applying each mitigation measure separately are described below. The results presented in sections 3.2.1 – 3.2.3 refer first to scenarios in which DT_{50} was set to 25

days, and then comparison to scenarios with DT_{50} of 1.3 days is described. The combined effects are shown in section 3.2.4.

3.2.1 Decreasing the toxicity of applied insecticides

Decreasing the toxicity of applied insecticides had positive impact on beetle populations in all analysed study areas, but its magnitude varied considerably and was substantially influenced by landscape and farmland heterogeneity. In all scenarios reducing insecticides' toxicity, both the mean overall beetle density and Occupancy increased substantially relative to the baseline (Fig. 5). This was not however, the case for the mean beetle Abundance, which in some study areas was almost stable or even somewhat decreased (Fig. 5) due to increased Occupancy. The magnitude of observed impacts was higher in scenarios with DT_{50} of 1.3 days (Fig. 5B) than 25 days (Fig. 5A).

In scenarios with DT_{50} of 25 days, decreasing insecticides' toxicity from high (LR90) to medium (LR50) increased the mean overall beetle density up to 11%, and mean Occupancy up to 10%. Further reduction of insecticides' toxicity to LR25 resulted in relative increases exceeding 20% and 15% for the mean overall beetle density and Occupancy, respectively. The relative change in the mean overall beetle density and Occupancy decreased with the area of animal farms in a landscape ($p < 0.01$; Table C4 and C6 in Appendix C). The study areas most positively impacted were those with the lowest baseline beetle populations, i.e., those with the lowest area of managed grasslands in a landscape and, at the same time, high proportion of arable vegetable farms (study areas 4, 5 and 10). In addition, a high increase in mean overall beetle density and Occupancy was found in the study area 3 dominated by potato farms. Mean beetle Abundance was impacted more substantially only when insecticides' toxicity was decreased to LR25, but still for study areas 4 and 5 the Abundance had not reached the baseline values (Fig. 5A). The relative change in the mean beetle

Abundance increased with the number of fields in a landscape ($p = 0.02$; Table C5 in Appendix C).

The relative increase in mean beetle Occupancy in response to decreasing insecticides toxicity was up to 5% higher in scenarios with DT_{50} of 1.3 days (Fig. 5B) than 25 days (Fig. 5A), while for beetle Abundance the difference was up to + 3% (Fig. 5B). In scenarios with DT_{50} of 1.3 days, the relative change in mean overall beetle density and Occupancy not only decreased with the area of animal farms in a landscape ($p < 0.01$ for both beetle density and Occupancy) as in scenarios with DT_{50} of 25 days, but also with the farm diversity ($p = 0.02$ for beetle density, and p = 0.03 for beetle occupancy). In addition, in scenarios with DT_{50} of 1.3 days relative change in mean beetle Occupancy was negatively impacted by the landscape diversity ($p = 0.01$; Table C4 and C6 in Appendix C). The regression model for the relative change in mean beetle Abundance was non-significant (Table C5 in Appendix C).

3.2.2 Increasing the level of drift reduction

No effect of spray drift reduction was detectable when insecticides were applied with low application rate (1.22) corresponding with DT_{50} of 25 days. When high application rate of insecticides (41.78) corresponding with DT_{50} of 1.3 days was used, effects of spray drift reduction on mortality were visible; however, its effect on beetle mortality was already very small with drift reduction of 50%. Therefore, increasing the reduction of pesticide drift from 50% to 90% had no significant effect on beetle populations in analysed study areas (the effect was smaller than in-between replicates variation).

3.2.3 Introducing grassy field margins

Introducing grassy field margins of 4-m width to 50% of cultivated fields in a landscape increased beetle population size in all study areas, but the impacts were smaller in comparison to toxicity-related scenarios. In addition, the magnitude of impacts did not differ substantially between scenarios with DT_{50} of 25 (Fig. 6A) and 1.3 days (Fig. 6B).

In scenarios with DT_{50} of 25 days, the relative changes in the mean overall beetle density and Occupancy decreased with the share of animal farms in a landscape ($p = 0.02$ for beetle density, and $p < 0.01$ for occupancy; Table C7 and C9 in Appendix C). Similar trends were found in scenarios with DT_{50} of 1.3 days, however the relative change in mean overall beetle density was also negatively influenced by farm diversity ($p = 0.02$; Table C7 in Appendix C), and relative change in mean beetle Occupancy increased with the number of fields in a landscape ($p = 0.05$; Table C9 in Appendix C). Introducing grassy field margins only slightly increased the mean beetle Abundance (up to 3%; Fig. 6), and the regression models for the relative change in mean beetle Abundance were found non-significant in scenarios with DT_{50} of both 25 and 1.3 days (Table C8 in Appendix C).

3.2.4 Combined effects

Application of combined mitigation measures (i.e., decreasing of insecticides toxicity and introduction of field margins) resulted in a substantial relative increase in both mean beetle Occupancy and Abundance (Fig. 7). The biggest impacts were noted for study area 10 with relative increase exceeding 15% for mean beetle abundance and 35% for beetle occupancy. In scenarios where insecticides with short environmental degradation time were used, those impacts were magnified, especially for mean beetle Abundance (Fig. 7).

4. Discussion and conclusions

Biodiversity-related agricultural polices aim at reducing negative impacts of pesticide use on farmland habitats and, at the same time, at increasing natural pest control through supporting populations of natural pest enemies. However, it has been suggested that the mitigation measures proposed in agri-environmental programs, even if tailored for a certain group of species, may not be successful if not also tailored to the ecological and spatial context of the specific agricultural landscape of interest [45]. In this study, by using a

combination of high-resolution dynamic landscape models and detailed spatially-explicit agent-based model, we were able to realistically simulate the behavior and population dynamics of non-target arthropod species *B. lampros* in the various agricultural landscapes of the Netherlands. The subsequent analysis allowed us to determine the major drivers of beetle populations in relation to different farming systems, as well as to assess the effectiveness of selected mitigation measures under various initial conditions (including varying landscape and farmland heterogeneity). The measures tested were suggested in the document 'Healthy Growth, Sustainable Harvest' on sustainable crop protection policy in the Netherlands for the 2013-2023 period [17] but are generally relevant at the EU level.

Below we consider the major facets of the beetle responses before drawing conclusions for agricultural policy measures.

4.1 Factors controlling beetle population size in agricultural landscapes

Simulated beetle populations under initial conditions (i.e., before mitigation measures were applied) differed considerably between landscape and farming systems. This confirms the importance of landscape structure for carabids populations in agroecosystems [9,46–48]. Beetle populations were larger and distributed over larger areas in more diverse landscapes with a high proportion of animal farms (i.e., extensive area of managed grasslands) and, at the same time, low area coverage of arable vegetable farms. Highly diverse landscapes have higher quantity of non-arable habitats, and therefore extended arable to non-arable boundaries supporting effective colonization of arable fields by beetles [10,49]. This implies that the starting conditions for assessment of policy instruments should be considered before an assessment of effects are made. In the Dutch farming systems, managed grasslands provide large areas free of insecticide spraying and intense soil cultivation. They can be thus favorable for beetles, especially if embedded in a landscape with high intensity use. [50] concluded that in mixed farming systems "grasslands and croplands complement each other" by providing

continuous and different resources to the species throughout the year". It is, however, important to note that in our simulations we did not consider possible negative effects of intensive grazing on beetles. Large herbivores may impact arthropods in various ways, by directly increasing mortality rate and disturbance or, indirectly, by changing vegetation structure of grasslands and their abiotic conditions [51]. However, if managed carefully, grasslands can make an important contribution to the biodiversity of agricultural landscapes, at the same time supporting population-based regulating services (e.g., pollination and biological control) [52]. More diverse landscapes also promote other non-target arthropods, including spiders [53], and butterflies [54,55]. The amount and proximity of grassy habitats are known to strongly drive carabids communities [50,56] but also to support, in general, biodiversity in agricultural landscapes [57,58]. This suggests that policy goals could partly be achieved by promotion of mixed farming strategies.

Local beetle Abundance, unlike Occupancy, was harder to predict as it is a result of dynamics between source and sink habitats, and their distribution within the landscape. The fact that beetle density in occupied areas was negatively influenced by the number of fields in a landscape seems incongruous at first. However, the number of fields in a landscape was correlated with number of farms in our study areas, and larger number of farms could increase crop rotation and diversity. This would normally be considered a good thing, but as a result insecticide applications were more evenly distributed within the landscape (occurring in many small but scattered fields). This effectively increased the treated area:edge ratio and subsequent mortality occurring at this boundary [21]. Like flying insects that are evenly redistributed across agricultural landscapes [59], if we have an evenly distributed population of even somewhat mobile beetles, and the distribution of toxic locations are many and evenly spread, the probability of beetle exposure increases considerably.

4.2 Key factors in the effectiveness of mitigation measures

Here we compared effectiveness of selected measures from two groups, pesticide- and landscape-related. Effects of drift-reducing measures at the population-level were of little importance. This is almost certainly due to the fact that at the time of spraying the population was largely present in the field, hence exposure was reduced marginally by the drift-reduction. Much larger benefits resulted from reduction of insecticides toxicity, and these generally exceeded those associated with introduction of additional field margins of 4-m width to 50% of cultivated fields (Fig. 8). Such effects were expected, as already indicated by other ALMaSS simulation studies [22,26]. The magnitude of these effects and the interplay between beetle Abundance and Occupancy were, however, strongly driven by the landscape context. Depending on the starting context (landscape and farmland heterogeneity), the results of the mitigation measures maybe to predominantly increase the range of the beetles or increase their local abundance, or influence both strongly. For a high initial beetle Occupancy (> 70%), decreasing insecticide toxicity had minimal impacts on increased Occupancy because insecticide pressure on beetles was low even before the mitigation measure were applied, and populations were limited by other factors. However, Abundance could increase in the same scenario. On the other hand, in areas with low initial beetle Occupancy, decreasing insecticidal toxicity permitted more rapid colonization of new areas but with low Abundance. In such study areas substantial increase in beetle Abundance (> 5%) could only be achieved when pesticide- and landscape-related measures were combined (Fig. 8). This confirms that in mixed farming systems field margins are important rather as a supporting not stand-alone mitigation measure, as they generally improve effects of reduction of insecticide toxicity [26].

The impacts of mitigation measures analyzed were modified by the insecticide's half-life (DT_{50}). In general, in scenarios with a low DT_{50} of 1.3 days, positive effects of toxicity-related mitigation measures were higher compared to long-persistence insecticides (Fig. 8).

Pesticides with shorter half-lives have shorter periods during which toxic levels are exceeded, and therefore allow beetle populations to recover more easily. Because of the toxic-unit approach used, initial mortality rates were not very different for high and low persistence pesticide, but the period of population influence was. In agreement with the general assumptions, this may suggest shifting towards low persistent insecticides would be beneficial, but only if this did not result in an increase in number of treatments, which was not considered in this study.

4.3 Consequences for agro-policy

The important lesson from this study is that population responses to applied mitigation measures can be very complex and depending on a landscape context. The decision should be made in relation to farming system, policy aim, and landscape structure. This would suggest the need for implementation of locally-adapted agro-polices. Nevertheless, some general recommendations can be made based on our results.

Shift towards 'low-risk' products as a priority. In general, decreasing insecticides toxicity was more effective in supporting beetle populations. Promoting shift towards 'low-risk' plant protection products, proposed in the Duch policy, should be, thus, the foremost priority, especially in areas dominated by intensively sprayed crop and plant species, such as potatoes or flower bulbs.

Drift reduction not a useful measure for promoting in-field ecosystem services. Perhaps obvious, but drift reduction is targetted at species living in the surround habitats, not those that live in the field itself at the time of spraying. This result does not mean that drift reduction is not a good thing, but it needs to be considered in the context of the policy goals.

Mitigation measure should reflect management goal. The simulations showed that mitigation strategies tested individually (especially decreasing toxicity of applied insecticides) were more effective at supporting increase in beetle's Occupancy than Abundance in study

areas dominated by rotational farming system. This is a desirable outcome, as by increasing beetle occupancy we are increasing number of fields subject to service, rather than focusing on improvement of pest control only in a few, selected fields. In an agricultural system dominated by permanent crops with coincidence between beetle distribution and crops, abundance increases may be more desirable. Such management goals require the implementation of combined mitigation strategies, which would not only decrease the pesticide pressure but also increase the amount of source habitats in a landscape. However, once again, we should consider the management goal; here we assumed pest control, but if it was conservation then an increase in occupancy is likely to be more highly favored.

The effectiveness of mitigation measures are modified by the spatial dynamic factors. This means that, although general effects could be predictable, the actual magnitude of these effects is highly variable and modified by various spatial dynamic factors (spatial distribution of source and sink habitats or underlying habitat suitability). In general, increasing heterogeneity in agricultural landscapes through introduction of refuges such as field margins, beetle banks or hedgerows is desirable. Even in areas highly exposed to insecticides, the non-target arthropods can be supported by ensuring enough source habitats, allowing populations to recover [26]. However, an important but often neglected factor here is the spatial distribution of insecticide application in a landscape. In farming systems with many scattered fields with intensively treated crops, the relative beetle populations are much more affected [21], even though local density in other areas can be high. This context needs to be taken into account when promoting farmland heterogeneity and crop diversity, and when determining both policy goals and the metrics used to measure policy impact. Once again, the specific context is important. Musters et al. [59] suggest that for flying insects the scale should be spatially large because of the dispersal ability, but for *Bembidion* the smaller landscape scale seems sufficient.

In conclusion, if we have a well-defined population target, the simulation approach used here provides a powerful tool to assess policy measures and their impact on key ecosystem service providers. These tools could be used to support innovative policy management towards goals such as those outlined in the Green Deal. However, care must be taken to properly define the specific metrics to be used to measure effects, and to understand the implications of changes in these metrics in population terms. Due to the degree of context specificity, once defined these metrics should not be taken out of the context of the key ecosystem service provider they are targeted towards. For example, *Bembidion* metrics should not be used to assess likely impacts of policy on pollinators.

Appendices

Appendix A: Parametrization of ALMaSS landscape model for the Dutch landscapes

Appendix B: Structural and farming heterogeneity of studied landscapes

Appendix C: Results – supplementary data

Acknowledgments

This research was supported by the PBL Netherlands Environmental Assessment Agency through the project "Developing and application of a methodology to assess impacts of pesticides on key ecosystem services".

References

1. Biesmeijer JC, Roberts SPM, Reemer M, Ohlemüller R, Edwards M, Peeters T, et al. Parallel declines in pollinators and insect-pollinated plants in Britain and the Netherlands. Science. 2006;313(5785): 351–354. doi: 10.1126/science.112786.
2. Hallmann CA, Sorg M, Jongejans E, Siepel H, Hofland N, Schwan H, et al. More than 75 percent decline over 27 years in total flying insect biomass in protected areas. PLoS One. 2017;12(10): e0185809. doi: 10.1371/journal.pone.0185809.
3. Hallmann CA, Zeegers T, Klink R, Vermeulen R, Wielink P, Spijkers H, et al. Declining abundance of beetles, moths and caddisflies in the Netherlands. Insect Conserv Divers. 2020;13(2): 127–139. doi: 10.1111/icad.12377.
4. Schuch S, Meyer S, Bock J, van Klink R, Wesche K. Drastische Biomasseverluste bei Zikaden verschiedener Grasländer in Deutschland innerhalb von sechs Jahrzehnten. Natur und Landschaft. 2019;94(4): 141–145. doi: 10.17433/4.2019.50153677.
5. Van Swaay CAM, Dennis EB, Schmucki R, Al. E. The EU Butterfly Indicator for Grassland species: 1990-2017. Technical Report. Butterfly Conservation Europe; 2019.
6. Sánchez-Bayo F, Wyckhuys KAG. Worldwide decline of the entomofauna: A review of its drivers. Biological Conservation. 2019;232: 8–27. doi: 10.1016/j.biocon.2019.01.020.
7. Wagner DL. Insect Declines in the Anthropocene. Annu Rev Entomol. 2020;65(1): 457–480. doi: 10.1146/annurev-ento-011019-025151.
8. Brooks DR, Bater JE, Clark SJ, Monteith DT, Andrews C, Corbett SJ, et al. Large carabid beetle declines in a United Kingdom monitoring network increases evidence for a widespread loss in insect biodiversity. J Appl Ecol. 2012;49(5): 1009–1019. doi: 10.1111/j.1365-2664.2012.02194.x.

9. Holland JM, Luff ML. The effects of agricultural practices on Carabidae in temperate agroecosystems. Integr Pest Manag Rev. 2000;5: 109–129. doi: 10.1023/A:1009619309424.

10. Bianchi FJJ, Booij CJH, Tscharntke T. Sustainable pest regulation in agricultural landscapes: a review on landscape composition, biodiversity and natural pest control. Proc Biol Sci. 2006;273(1595): 1715–1727. doi: 10.1098/rspb.2006.3530.

11. Carvalheiro LG, Veldtman R, Shenkute AG, Tesfay GB, Pirk CWW, Donaldson JS, et al. Natural and within-farmland biodiversity enhances crop productivity. Ecol Lett. 2011;14(3): 251–259. doi: 10.1111/j.1461-0248.2010.01579.x.

12. Chagnon M, Kreutzweiser D, Mitchell ED, Morrissey C, Noome D, Van der Sluijs JP. Risks of large-scale use of systemic insecticides to ecosystem functioning and services. Environ Sci Pollut Res. 2014;22: 119–134. doi: 10.1007/s11356-014-3277-x.

13. Hass AL, Kormann UG, Tscharntke T, Clough Y, Baillod AB, Sirami C, et al. Landscape configurational heterogeneity by small-scale agriculture, not crop diversity, maintains pollinators and plant reproduction in western Europe. Proc R Soc B Biol Sci. 2018;285: 20172242. doi: 10.1098/rspb.2017.2242.

14. Cardoso P, Barton PS, Birkhofer K, Chichorro F, Deacon C, Fartmann T, et al. Scientists' warning to humanity on insect extinctions. Biol Conserv. 2020;242: 108426. doi: 10.1016/j.biocon.2020.108426.

15. Geiger F, Bengtsson J, Berendse F, Weisser WW, Emmerson M, Morales MB, et al. Persistent negative effects of pesticides on biodiversity and biological control potential on European farmland. Basic Appl Ecol. 2010;11(2): 97–105. doi: 10.1016/j.baae.2009.12.001.

16. Perdikis D, Fantinou A, Lykouressis D. Enhancing pest control in annual crops by conservation of predatory Heteroptera. Biological Control. 2011;59: 13–21. doi: 10.1016/j.biocontrol.2011.03.014.

17. Nota Gezonde Groei, Duurzame Oogst [policy document 'Healthy Growth, Sustainable Harvest']. Ministry of Economic Affairs. The Hague; 2013.

18. Tiktak A, Bleeker A, Boezemanm van DJ, et al. A closer look at integrated pest management. Interim assessment of the policy document 'Healthy Growth, Sustainable Harvest'. English summary of the Dutch report "Geïntegreerde gewasbescherming nader beschouwd. Tussenevaluatie van de nota Gezonde Groei, Duurzame Oogst". PBL Netherlands Environmental Assessment Agency; 2019.

19. Eurostat. Agriculture statistics - Farm structure for 2018. Available from https://ec.europa.eu/eurostat/web/agriculture/data/database.

20. Topping CJ, Aldrich A, Berny P. Overhaul environmental risk assessment for pesticides. Science. 2020;367(6476): 360–363. doi: 10.1126/science.aay1144.

21. Topping CJ, Lagisz M. Spatial Dynamic Factors Affecting Population-Level Risk Assessment for a Terrestrial Arthropod: An Agent-Based Modeling Approach. Hum Ecol Risk Assess. 2012;18(1):168–180. doi: 10.1080/10807039.2012.632292.

22. Topping CJ, Craig PS, de Jong F, Klein M, Laskowski R, Manachini B, et al. Towards a landscape scale management of pesticides: ERA using changes in modelled occupancy and abundance to assess long-term population impacts of pesticides. Sci Total Environ. 2015;537: 159–169. doi: 10.1016/j.scitotenv.2015.07.152.

23. Dalkvist T, Sibly RM, Topping CJ. Landscape structure mediates the effects of a stressor on field vole populations. Landsc Ecol. 2013;28(10): 1961–1974. doi: 10.1007/s10980-013-9932-7.

24. Langhammer M, Grimm V, Pütz S, Topping CJ. A modelling approach to evaluating the effectiveness of Ecological Focus Areas: The case of the European brown hare. Land use policy. 2017;61: 63–79. doi: 10.1016/j.landusepol.2016.11.004.

25. Topping CJ, Dalby L, Valdez JW. Landscape-scale simulations as a tool in multi-criteria decision making to support agri-environment schemes. Agric Syst. 2019;176: 102671. doi: 10.1016/j.agsy.2019.102671.

26. Ziółkowska E, Topping CJ, Bednarska AJ, Laskowski R. Supporting non-target arthropods in agroecosystems: Modelling effects of insecticides and landscape structure on carabids in agricultural landscapes. Sci Total Environ. 2021;774:145746. doi: 10.1016/j.scitotenv.2021.145746.

27. Topping CJ, Hansen TS, Jensen TS, Jepsen JU, Nikolajsen F, Odderskær P. ALMaSS, an agent-based model for animals in temperate European landscapes. Ecol Modell. 2003;167(1–2): 65–82. doi: 10.1016/S0304-3800(03)00173-X.

28. Topping CJ, Hoye TT, Odderskaer P, Aebischer NJ. A pattern-oriented modelling approach to simulating populations of grey partridge. Ecol Modell. 2010;221(5): 729–737. doi: 10.1016/j.ecolmodel.2009.11.004.

29. Kromp B. Carabid beetles in sustainable agriculture: a review on pest control efficacy, cultivation impacts and enhancement. Agric Ecosyst Environ. 1999;74(1–3): 187–228. doi: 10.1016/S0167-8809(99)00037-7.

30. Symondson WOC, Sunderland KD, Greenstone MH. Can Generalist Predators be Effective Biocontrol Agents? Annu Rev Entomol. 2002;47(1): 561–594. doi: 10.1146/annurev.ento.47.091201.145240.

31. Bohan DA, Boursault A, Brooks DR, Petit S. National-scale regulation of the weed seedbank by carabid predators. J Appl Ecol. 2011;48(4): 888–898. doi: 10.1111/j. 1365-2664.2011.02008.x.

32. Kulkarni SS, Dosdall LM, Willenborg CJ. The Role of Ground Beetles (Coleoptera: Carabidae) in Weed Seed Consumption: A Review. Weed Sci. 2015;63(2): 355–376. doi: 10.1614/WS-D-14-00067.1.

33. Saska P, Vodde M, Heijerman T, Westerman P, van der Werf W. The significance of a grassy field boundary for the spatial distribution of carabids within two cereal fields. Agric Ecosyst Environ. 2007;122(4): 427–434. doi: 10.1016/j.agee.2007.02.013.

34. Bilde T, Topping C. Life history traits interact with landscape composition to influence population dynamics of a terrestrial arthropod: A simulation study. Ecoscience. 2004;11(1): 64–73. doi: 10.1080/11956860.2004.11682810.

35. Topping CJ, Dalby L, Skov F. Landscape structure and management alter the outcome of a pesticide ERA: evaluating an endocrine disruptor using the ALMaSS European Brown Hare model. Sci Total Environ. 2016;541: 1477–1488. doi: 10.1016/j.scitotenv.2015.10.042.

36. ESRI. ArcGIS Desktop: Release 1041, Redlands, CA: Environmental Systems Research Institute; 2020.

37. McGarigal K, Cushman SA, Ene E. FRAGSTATS v4: Spatial Pattern Analysis Program for Categorical and Continuous Maps. Computer software program produced by the authors at the University of Massachusetts, Amherst [Internet]. 2012. Available from: http://www.umass.edu/landeco/research/fragstats/fragstats.html.

38. Thorbek P, Bilde T. Reduced numbers of generalist arthropod predators after crop management. J Appl Ecol. 2004;41(3): 526–538. doi: 10.1111/j.0021-8901.2004.00913.x.

39. EFSA PPR Panel. Opinion on a request from EFSA related to the default Q10 value used to describe the temperature effect on transformation rates of pesticides in soil. EFSA J. 2007;622: 1–32. doi: 10.2903/j.efsa.2008.622.

40. Rautmann D, Streloke M, Winkler R. New basic drift values in the authorization procedure for plant protection products. In: Forster R, Streloke M, editors. Workshop on risk assessment and risk mitigation measures in the context of authorization of plant protection products (WORMM). Berlin-Dahlem: Mitteilungen Biologische Bundesanstalt Land-Forstwirtschaft; 2001. p. 133–41.

41. Leendertse P, Lageschaar L, Hoftijser E, Al. E. Tussenevaluatie Gezonde Groei, Duurzame Oogst (GGDO): Geïntegreerde gewasbescherming. CLM-publicatie 968, CLM, Culemborg; 2019.

42. Uitvoeringsprogramma Toekomstvisie gewasbescherming 2030 [policy document 'Vision on crop protection 2030']. Ministry of Agriculture, Nature and Food Quality. The Hague; 2019.

43. Høye TT, Skov F, Topping CJ. Interpreting outputs of agent-based models using abundance-occupancy relationships. Ecol Indic. 2012;20: 221–227. doi: 10.1016/j.ecolind.2012.01.017.

44. R Core Team. R: A language and environment for statistical computing [Internet]. Vienna, Austria: R Foundation for Statistical Computing; 2013. Available from: www.R-project.org.

45. Kleijn D, Berendse F, Smit R, Gilissen N, Smit J, Brak B, et al. Ecological Effectiveness of Agri-Environment Schemes in Different Agricultural Landscapes in The Netherlands. Conservation Biology. 2004;18: 775-786. doi: 10.1111/j.1523-1739.2004.00550.x.

46. Vanbergen AJ, Woodcock BA, Watt AD, Niemelä J. Effect of land-use heterogeneity on carabid communities at the landscape scale. Ecography. 2005;28(1): 3–16. Available from: https://www.jstor.org/stable/3683443.

47. Vasseur C, Joannon A, Aviron S, Burel F, Meynard J-M, Baudry J. The cropping systems mosaic: How does the hidden heterogeneity of agricultural landscapes drive arthropod populations? Agric Ecosyst Environ. 2013;166:3–14. doi: 10.1016/j.agee.2012.08.013.

48. Bertrand C, Burel F, Baudry J. Spatial and temporal heterogeneity of the crop mosaic influences carabid beetles in agricultural landscapes. Landsc Ecol. 2016;31(2): 451–466. doi: 10.1007/s10980-015-0259-4.

49. Bianchi FJJA, Van Der Werf W. The Effect of the Area and Configuration of Hibernation Sites on the Control of Aphids by *Coccinella septempunctata* (Coleoptera: Coccinellidae) in Agricultural Landscapes: A Simulation Study. Environ Entomol. 2003;32(6): 1290–1304. doi: 10.1603/0046-225X-32.6.1290.

50. Massaloux D, Sarrazin B, Roume A, Tolon V, Wezel A. Complementarity of grasslands and cereal fields ensures carabid regional diversity in French farmlands. Biodivers Conserv. 2020;29(9–10): 2861–2882. doi: 10.1007/s10531-020-02002-9.

51. van Klink R, van der Plas F, van Noordwijk CGET, Wallisdevries MF, Olff H. Effects of large herbivores on grassland arthropod diversity. Biol Rev. 2015;90(2): 347–366. doi: 10.1111/brv.12113.

52. Bengtsson J, Bullock JM, Egoh B, Everson C, Everson T, O'Connor T, et al. Grasslands—more important for ecosystem services than you might think. Ecosphere. 2019;10(2): e02582. doi: 10.1002/ecs2.2582.

53. Schmidt MH, Roschewitz I, Thies C, Tscharntke T. Differential effects of landscape and management on diversity and density of ground-dwelling farmland spiders. J Appl Ecol. 2005;42(2): 281–287. Available from: https://www.jstor.org/stable/3505721.

54. Weibull A-C, Bengtsson J, Nohlgren E. Diversity of butterflies in the agricultural landscape: the role of farming system and landscape heterogeneity. Ecography. 2000;23: 743–750. doi: 10.1111/j.1600-0587.2000.tb00317.x.

55. Archaux F, Lorel C, Villemey A. Landscape drivers of butterfly and burnet moth diversity in lowland rural areas. Landsc Ecol. 2018;33(10): 1725–1739. doi: 10.1007/s10980-018-0697-x.
56. Hendrickx F, Maelfait JP, Van Wingerden W, Schweiger O, Speelmans M, Aviron S, et al. How landscape structure, land-use intensity and habitat diversity affect components of total arthropod diversity in agricultural landscapes. J Appl Ecol. 2007;44(2): 340–351. doi: 10.1111/j.1365-2664.2006.01270.x.
57. Arponen A, Heikkinen RK, Paloniemi R, Pöyry J, Similä J, Kuussaari M. Improving conservation planning for semi-natural grasslands: Integrating connectivity into agri-environment schemes. Biol Conserv. 2013 Apr;160: 234–241. doi: 10.1016/j.biocon.2013.01.018.
58. Harlio A, Kuussaari M, Heikkinen RK, Arponen A. Incorporating landscape heterogeneity into multi-objective spatial planning improves biodiversity conservation of semi-natural grasslands. J Nat Conserv. 2019;49: 37–44. doi: 10.1016/j.jnc.2019.01.003.
59. Musters CJM, Evans TR, Wiggers JMR, van 't-Zelfde M, de Snoo GR. Distribution of flying insects across landscapes with intensive agriculture in temperate areas. Ecol Indic. 2021;129: 107889. doi: 10.1016/j.ecolind.2021.107889.

Tables

Table 1. Metrics used to characterize heterogeneity of studied landscapes.

Type	Metric	Explanation	Range (min – max)
Landscape heterogeneity	Coverage of herbaceous habitats	% of herbaceous (without managed grasslands) in a landscape.	4.57 – 11.70 [%]
	Coverage of woodland habitats	% of woodland habitats in a landscape.	1.33 – 12.46 [%]
	Landscape diversity	Shannon's diversity index (≥ 0, without limit) of landscape element types including six categories: arable land, herbaceous semi-natural habitats, woodland (woody semi-natural habitats), build-up areas, water and others. Shannon's diversity index = 0 when landscape contains only 1 patch (i.e., no diversity), and increases as the number of different patch types increases and/or proportional distribution of area among patch types becomes more equitable.	0.71 – 1.35
	Landscape shape index	Normalized ratio of edge (i.e., patch perimeters) to area (class or landscape) in which the total length of edge is compared to a landscape with a standard shape (square) of the same size and without any internal edge. Values greater than one indicate increasing levels of internal edge and corresponding decreasing aggregation of patch types.	93.58 – 170.92
Farmland heterogeneity	Farming diversity	Shannon diversity index calculated for fields categorized into farm types (see Table A4 in Appendix A).	0.81 – 1.81
	Share of animal farms	% of farms in a landscape with >70% of grassland (permanent and perennial grassland) in the production profile (see Table A4 and Table A5 in Appendix A).	8.02 – 85.16 [%]
	Number of fields	Number of fields in a landscape.	1195 – 3718
	Mean field size	Mean field size.	1.40 – 6.65 [ha]
	Field boundaries density	Perimeter of field boundaries to total cultivated area.	0.025 – 0.048 [1/m]

Figure 6. Changes in mean beetle occupancy and abundance of *B. lampros* populations (plot of Abundance to Occupancy Relationship, AOR) in scenarios introducing additional field margins and with DT_{50} of (A) 25 days or (B) 1.3 days: (1) absolute changes, (2) relative changes in comparison to the baseline scenario (with LR of 90%, drift reduction of 50%, and no additional field margins added). Dots mark consecutively, starting from the bottom, the baseline scenario (0, 0), and changes in AOR resulted from decreasing the insecticides' toxicity to LR50, and LR25.

Figure 7. Relative changes in mean beetle occupancy and abundance of *B. lampros* populations (plot of Abundance to Occupancy Relationship, AOR) in analysed study areas when comparing the "worst" and the "best" scenario, i.e., the toxicity is reduced from high to low, and field boundaries of 4-m width are added to 50% of fields in a landscape. Effects showed for scenarios with DT_{50} of (A) 25 days or (B) 1.3 days.

Figure 8. Relative impacts of analysed mitigation strategies on *Bembidion lampros* populations in relation to landscape structure. Mitigation strategy of increasing reduction of drift was omitted due to non-significant effect. LSI stands for landscape shape index and is a proxy of disaggregation of habitat patches. Fields fragmentation was defined as a measure combining number of fields, mean size of fields and field boundaries density (for more explanation see Appendix B, Tables B2-B3).

Figure Legends

Figure 1. Farming structure in the study areas, and study areas location in relation to soil type map. Source of soil data: Grondsoortenkaart published by Alterra, Wageningen University & Research (https://www.wur.nl/nl/show/Grondsoortenkaart.htm).

Figure 2. Structural (A) and farming (B) heterogeneity in studied test areas. Numbers in circles indicate study areas as shown in Fig. 1.

Figure 3. Summary of implemented mitigation measures. Three levels of insecticide toxicity defined in relation to insecticide-driven beetle field lethality rates, high (LR90), medium (LR50) and low (LR25), were tested in combination with drift reduction of 50% or 90% (only for scenario LR90) and additional grassy field margins not included (FM_OFF) or included (FM_ON) in a landscape. Eight combinations of these scenarios were tested for insecticides with two different environmental decay values: (A) DT_{50} of 25 days, and (B) DT_{50} of 1.3 day. These 16 combinations of scenarios were evaluated in ten study areas (Fig. 1).

Figure 4. The mean overall population density of *B. lampros* in 10 study areas (as shown in Fig. 1) according to the baseline ('worst-case') scenario (LR of 90%, insecticide drift reduction of 50%, and no additional field margins added to the landscape) with DT_{50} of 25 days (baseline scenario A; blue bars), and DT_{50} of 1.3 days (baseline scenario B; red bars).

Figure 5. Changes in mean beetle occupancy and abundance of *B. lampros* populations (plot of Abundance to Occupancy Relationship, AOR, in scenarios decreasing the insecticides toxicity and with DT_{50} of (A) 25 days or (B) 1.3 days: (1) absolute changes, (2) relative changes in comparison to the baseline scenario (with LR of 90%, drift reduction of 50%, and no additional field margins added). Dots mark consecutively, starting from the bottom, the baseline scenario (0, 0), and changes in AOR resulted from decreasing the insecticides' toxicity to LR50, and LR25.

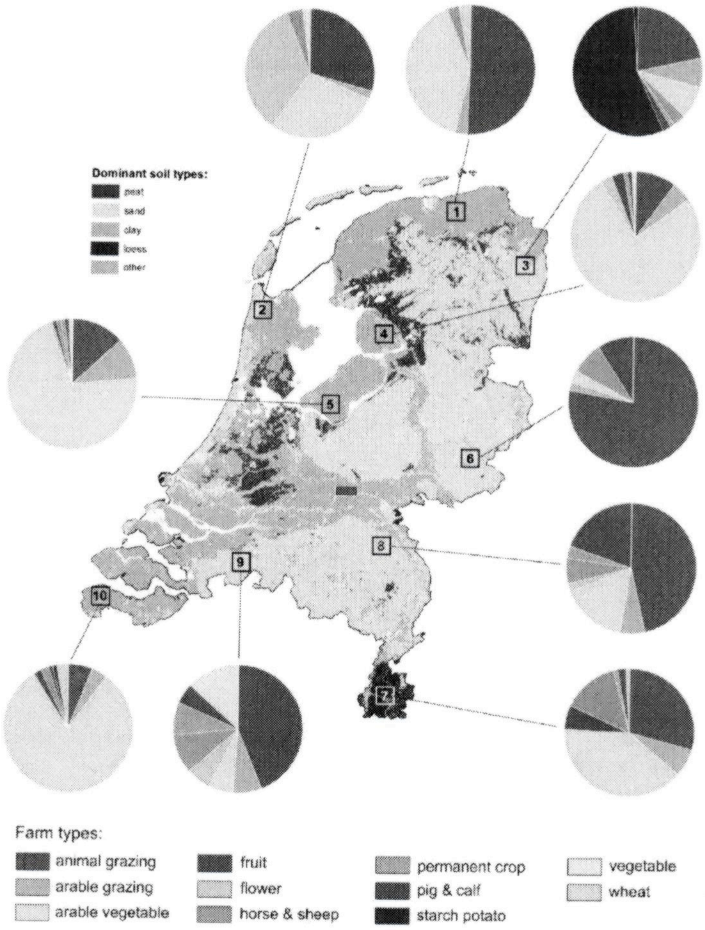
Dominant soil types:
peat
sand
clay
loess
other
1
2
3
4
5
6
7
8
9
10
Farm types:
animal grazing
arable grazing
arable vegetable
fruit
flower
horse & sheep
permanent crop
pig & calf
starch potato
vegetable
wheat

Figure 1

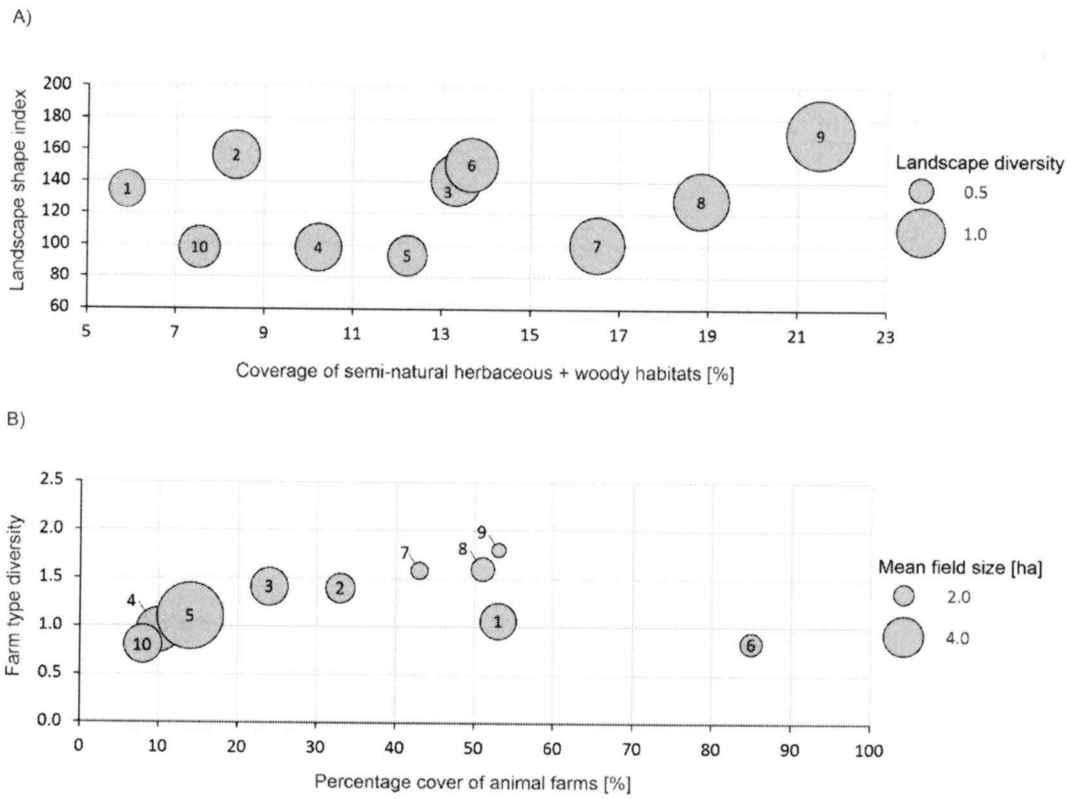
A)
Landscape shape index
Coverage of semi-natural herbaceous + woody habitats [%]
Landscape diversity
0.5
1.0
B)
Farm type diversity
Percentage cover of animal farms [%]
Mean field size [ha]
2.0
4.0

Figure 2

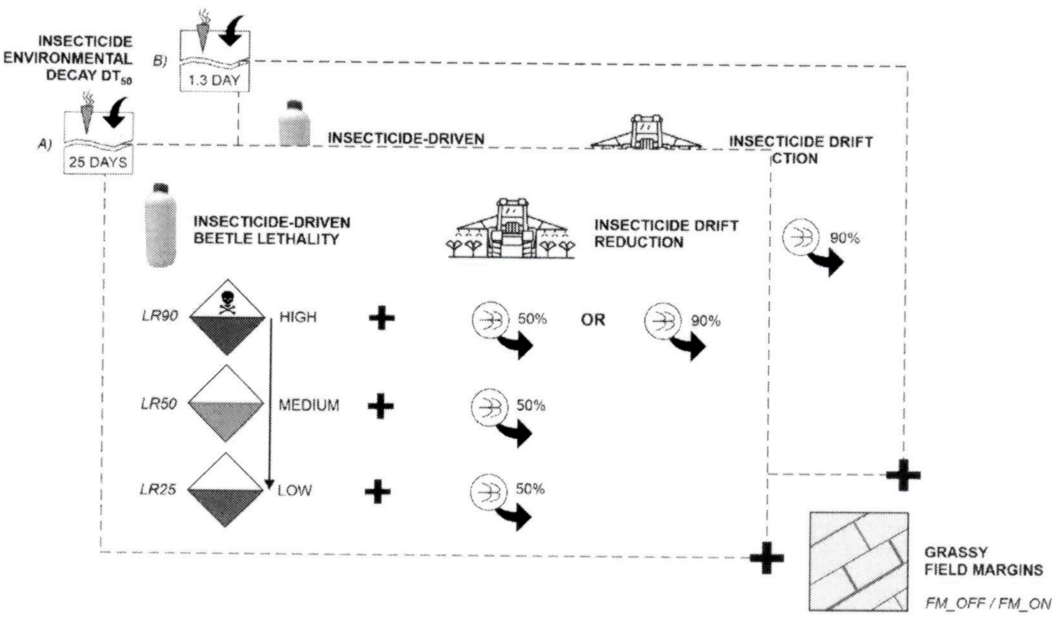
INSECTICIDE ENVIRONMENTAL DECAY DT50
B)
1.3 DAY
A)
25 DAYS
INSECTICIDE-DRIVEN
INSECTICIDE DRIFT
CTION
INSECTICIDE-DRIVEN BEETLE LETHALITY
INSECTICIDE DRIFT REDUCTION
90%
LR90
HIGH
50%
OR
90%
LR50
MEDIUM
50%
LR25
LOW
50%
GRASSY FIELD MARGINS
FM_OFF / FM_ON

Figure 3

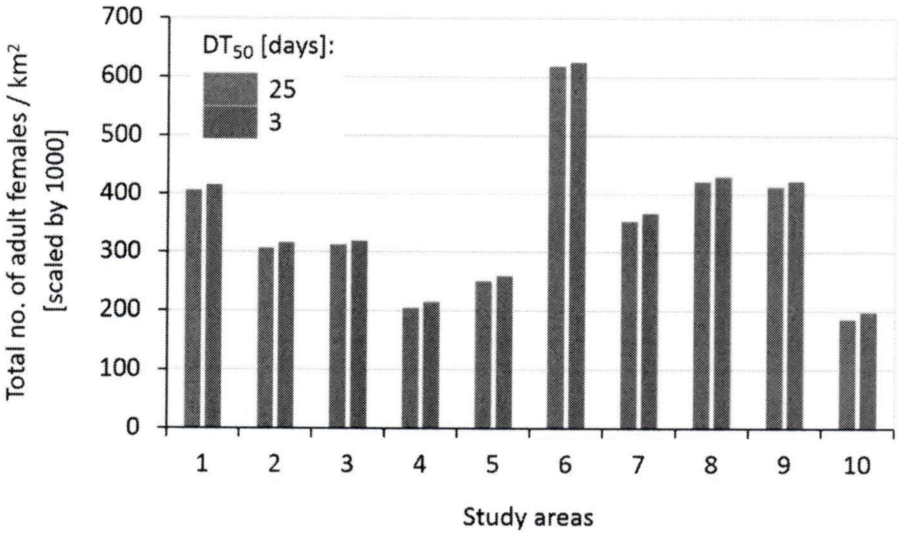

Total no. of adult females / km²
[scaled by 1000]
DT50 [days]:
25
3
0
100
200
300
400
500
600
700
1
2
3
4
5
6
7
8
9
10
Study areas

Figure 4

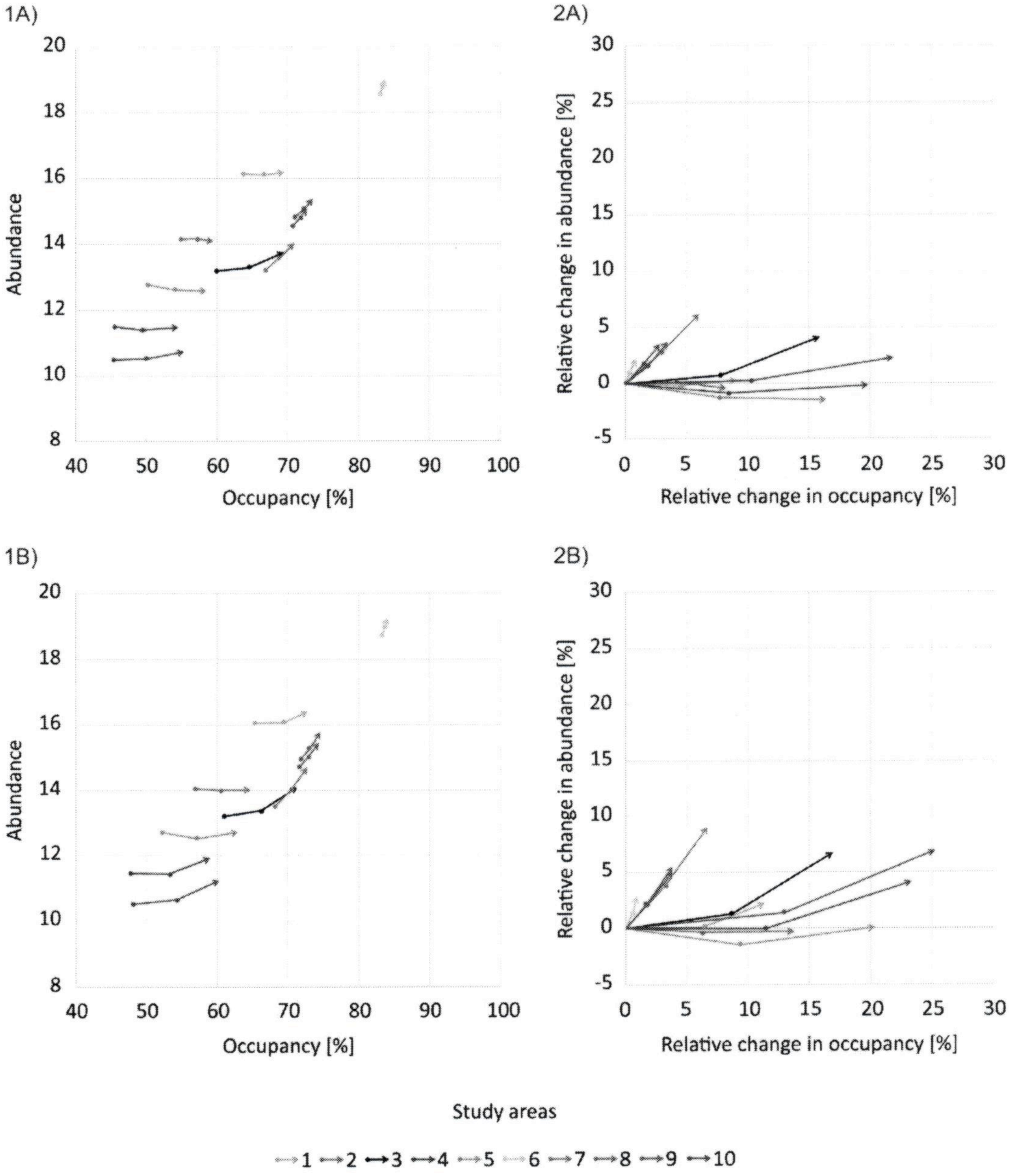
1A)
2A)
1B)
2B)
Abundance
Occupancy [%]
Relative change in abundance [%]
Relative change in occupancy [%]
Study areas
1 2 3 4 5 6 7 8 9 10

Figure 5

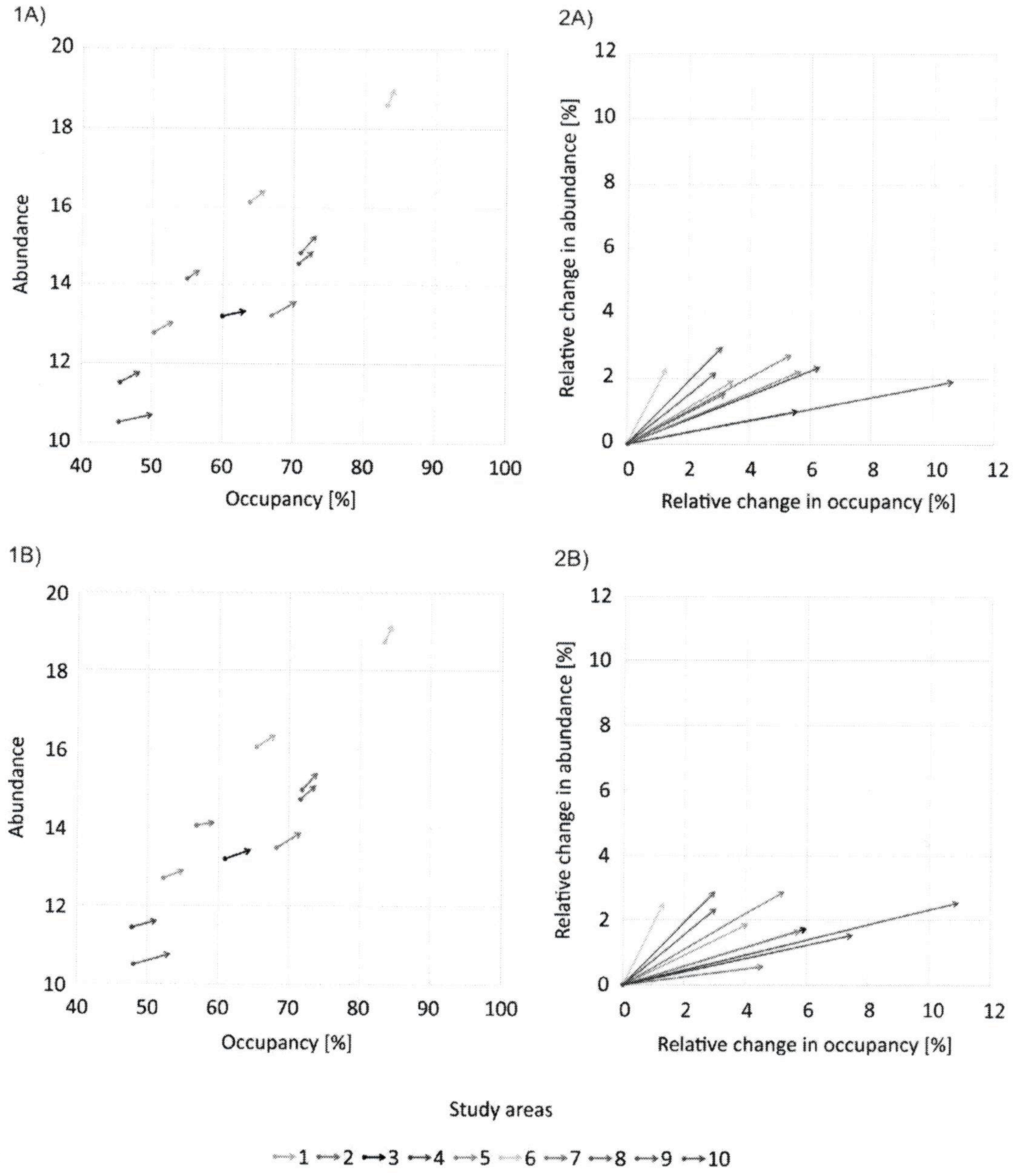
1A)
Abundance
Occupancy [%]
2A)
Relative change in abundance [%]
Relative change in occupancy [%]
1B)
Abundance
Occupancy [%]
2B)
Relative change in abundance [%]
Relative change in occupancy [%]
Study areas
1 2 3 4 5 6 7 8 9 10

Figure 6

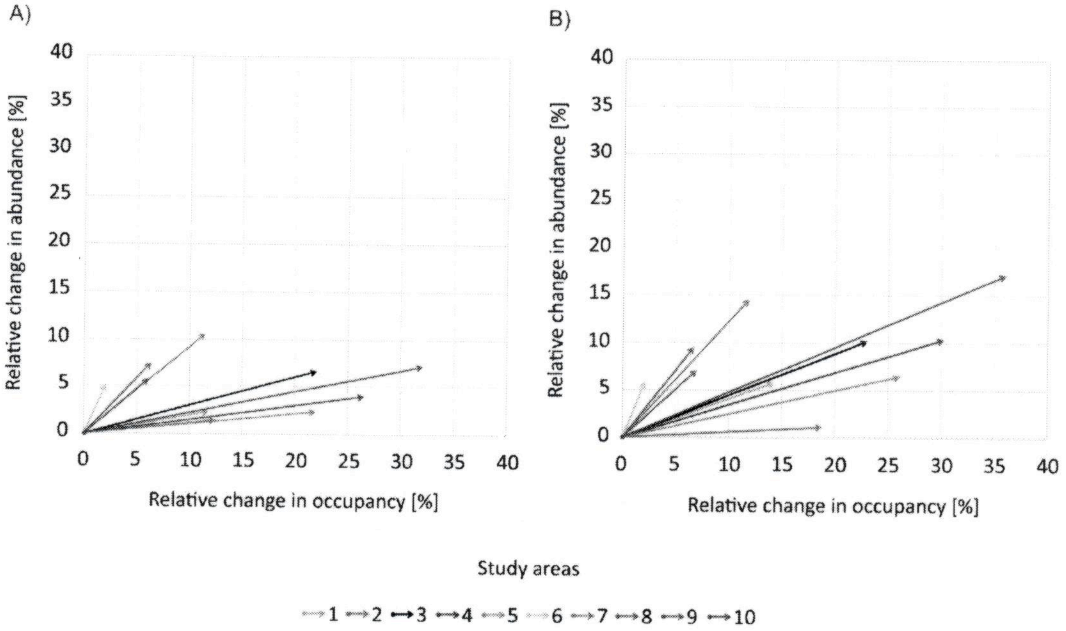
A)
B)
Relative change in abundance [%]
Relative change in occupancy [%]
0
5
10
15
20
25
30
35
40
Study areas
1 2 3 4 5 6 7 8 9 10

Figure 7

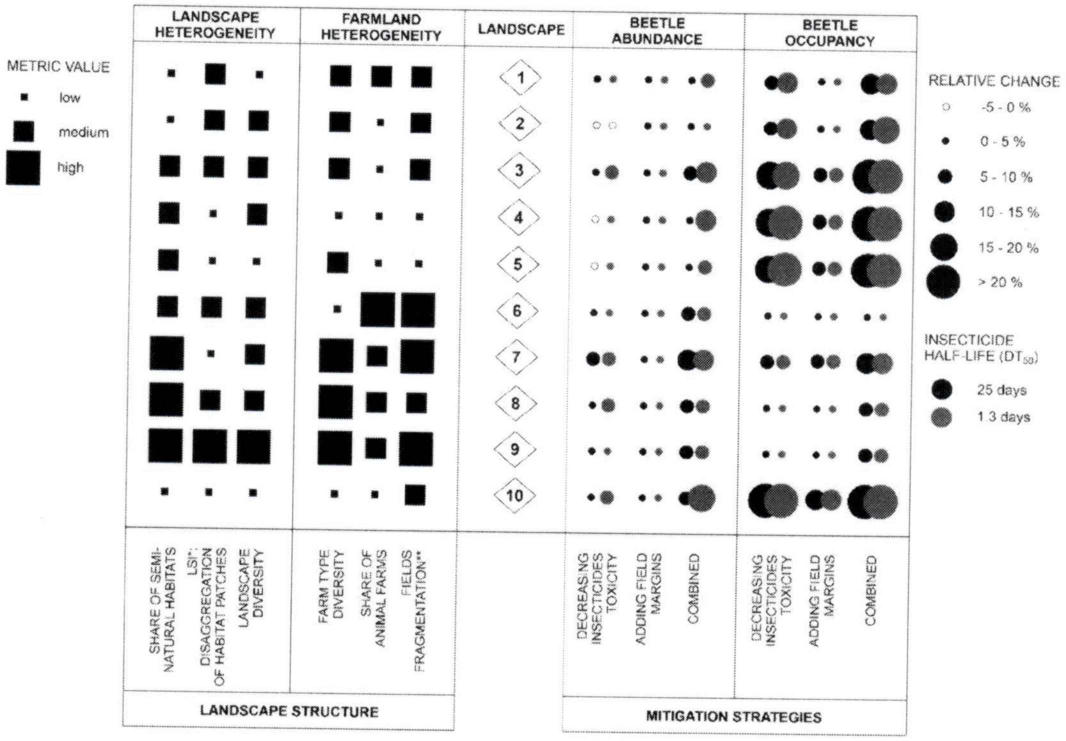
METRIC VALUE
low
medium
high
LANDSCAPE HETEROGENEITY
FARMLAND HETEROGENEITY
LANDSCAPE
BEETLE ABUNDANCE
BEETLE OCCUPANCY
1
2
3
4
5
6
7
8
9
10
RELATIVE CHANGE
-5 - 0 %
0 - 5 %
5 - 10 %
10 - 15 %
15 - 20 %
> 20 %
INSECTICIDE HALF-LIFE (DT50)
25 days
1.3 days
SHARE OF SEMI-NATURAL HABITATS
LSI*: DISAGGREGATION OF HABITAT PATCHES
LANDSCAPE DIVERSITY
FARM TYPE DIVERSITY
SHARE OF ANIMAL FARMS
FIELDS FRAGMENTATION**
DECREASING INSECTICIDES TOXICITY
ADDING FIELD MARGINS
COMBINED
DECREASING INSECTICIDES TOXICITY
ADDING FIELD MARGINS
COMBINED
LANDSCAPE STRUCTURE
MITIGATION STRATEGIES

Figure 8
