# Supplementary material for: Is the effectiveness of policy-driven mitigation measures on carabid populations driven by landscape and farmland heterogeneity? Applying a modelling approach in the Dutch agroecosystems
Source: PLoS One. 2022 Dec 27;17(12):e0279639. doi: 10.1371/journal.pone.0279639 (PMC9794068; doi:10.1371/journal.pone.0279639)
Supplement: Attachment — Submitted filename: Response_to_Reviewers_final.docx [file pone.0279639.s008.docx]

**Response to Reviewers and Editor**

We thank Referees and Editor for the helpful comments. Below we provide point-by-point answers to the points raised by the Reviewer #1 and Reviewer #2. All the line numbers refer to the revised manuscript with tracked changes.

**REVIEWER #1:**

1. *Overall, this is a nicely thought through and executed study and very relevant for current developments in environmental risk assessments and management. The main finding that reducing impacts on beetle survival and increasing field margins had the highest impact on population abundance and range is consistent with other studies exploring how perturbations of life history traits impacts population growth rate. In those studies, perturbations of survival rates consistently had the highest impact of population growth which is in line with the findings of this study, whereas here an additional factor was evaluated which essentially increased the capacity of the system. I agree with the authors about the role simulation tools should increasingly play in risk assessment and management and environmental policy and the authors provide a nice example of how this could be done.*

Thank you for these comments. We are happy that our manuscript was well received.

2. *The manuscript is written well, but what I am missing is sufficient information on the biology and ecology of the modeled species which prevents from fully evaluating the analysis and results. The reader should be at least somewhat familiar with the basic ecology and habitat use of the modeled species, yet not even rudimentary descriptions have been provided. Rather the authors refer to old publications and to the model documentation which I checked, but it still does not provide enough relevant information on the species.*

Thank you for pointing this issue out. We agree with the Reviewer that more information on biology and ecology of the modelled species will allow the reader to better evaluate and understand the analysis and results. This information has been now added to section 2.2. The section has been divided into two sub-sections: 2.2.1 describing the species biology and ecology and 2.2.2 explaining implementation and model procedures in ALMaSS.

*Specific comments:*

3. *LN 22 – Bembidion*

Done.

4. *LN 116-126 - I can appreciate the decades of work that went into developing ALMaSS and given its complexity there is no really practical way of fully describing it in each and every publication. The model documentation should be a repository of all relevant information on the conceptual model, as well as on model testing and validation. I found the amount of information in the ODdox underwhelming and the reference to the Bilde&Topping 2004 paper not sufficient. That paper does not contain a detailed account of species biology and how it was implemented, the model evaluation is not included either. I would strongly urge the authors to provide a bit more information on the species, either in section 2.2 or in the Appendix, specifically on its life cycle, life history traits, habitat use, population regulation mechanisms, and dietary preferences. And of course, how such information was implemented in the model. This type of document needs to be produced once and can be re-used for any new publication.*

Thank you for this comment. As already mentioned in our answer to comment (2), we have substantially re-written section 2.2 to include more information on species biology and ecology, including specific points raised by the Reviewer.

5. *LN 223 - 10 x 10 km, not km2*

Done.

6. *LN 245 - Please provide a more information, i.e. the full function, for intraspecific predation.*

Details on intraspecific predation were added to section 2.2.2 (lines 290-294).

7. *LN 247 – 255 - I understand this is a case study and not meant as a realistic assessment of risk from pesticides, but the implementation of effects is very simplistic and at odds with the much more elaborate implementation of exposure in the landscape.*

We agree with the Reviewer that a more detailed implementation would be possible and the model may be always supplemented with new details. However, the implementation of effects used in this study matches the implementation presented in the EFSA Scientific Opinion addressing the state of the science on risk assessment of plant protection products for non-target arthropods (EFSA Journal 2015; 13(2):3996). It is even more elaborated than in the ‘traditional’ risk assessment of pesticides. It was shown that with this implementation it is possible to successfully investigate the potential of source–sink dynamics to indirectly affect untreated populations, the impact of long-term year-on year application of the pesticide and the effect of landscape structure on effects (see EFSA Scientific Opinion mentioned above).

8. *LN 283 - Always better to pull the actual parameter values out of equations and describe/define them in text.*

Thank you for this suggestion. We rewrote the equation as suggested (lines 332-339).

9. *LN 336-365 - In order to make this study somewhat reproducible, you should provide initial state and conditions of the model, as well as the scripts used for this specific study. You should also provide all the simulation data that are presented in this manuscript and ideally the scripts used for analysis and plotting. It doesn’t look like any of this was provided, making this study completely irreproducible.*

Thank you for this comment. We have uploaded landscape generation scripts, script producing landscape inputs for the ALMaSS and other associated files to an open repository on GitLab (https://gitlab.com/ALMaSS/almassauxillary/). The results of simulation runs are now available in supplementary data files. Supplementary Data File 1 contains results of simulations for DT50 of 25 days, while Supplementary Data File 2 for DT50 of 1.3 days. We have also made available all the ALMaSS configuration files for simulation scenarios (Appendix C).

10. *LN 373-375 - Did you conduct any testing to support the claim about very small effect when a longer half-life was applied?*

Thank you for this comment. What we reported is a small difference in results when insecticides with short (1.3 day) versus long (25 days) half-life were applied. This could be explained by the fact that the applied treatment rate was much higher for insecticides with DT50 = 1.3 days than for insecticides with DT50 = 25 days (see explanation in lines 340-345). We did not conduct any further testing in that regard.

11. *LN 401-405 - It is really hard to understand the impacts of different farm types when we weren’t provided enough information on habitat use by the modeled species.*

The information about habitat preferences was added to sub-section 2.2.1. In landscapes with high proportion of animal farms (and therefore managed grasslands) the total area treated by insecticides is low and thus the proportion of the landscape favourable for beetles is high. In arable vegetable farms and potato farms, the proportion of crops with high number of insecticide treatments is large, therefore in landscapes with high share of these types of farms populations of beetles are low.

12. *LN 429 - Information about baseline dynamics and variation across replicates should be more explicit and provided at the beginning of the results section.*

Thank you for this comment. We added this information at the beginning of section 3 (lines 425-429).

**REVIEWER #2:**

1. *My main criticism of the study is that, as with most modeling ventures, the model is only as good as the assumptions made in it. In this particular case, little attention was given to the biology of the “model” species the authors chose. Little to no data or background is provided about the biology and ecology of this species. While I do agree it is abundant in the Netherlands, it must be pointed out it is classified primarily as a heathland species, and not one of major agricultural habitation (see Turin et al., 2022 – The Ecology and Conservation of Dutch Ground Beetles). The authors really provide no detail on why it was chosen as a model species, other than it is abundant and had been used in this type of model previously. However, because of the importance of ecosystem services to policy decisions, simulations should have included granivorous or omnivorous species as well, such as genera of Amara or Harpalus. Using a model species that is* *carnivorous missed important potential ecosystem services and would suggest the species is, perhaps, not truly a good model species for this modelling scenario.*

Thank you for this comment. It is important to underline that *Bembidion lampros* was used in this study as a representative species for group of spring-breeding, carnivorous ground beetles of temperate agricultural landscapes. This species is considered to be a useful natural enemy of pests in agricultural fields, and relevant for risk assessment of pesticides according to the EFSA Scientific Opinion addressing the state of the science on risk assessment of plant protection products for non-target arthropods (EFSA Journal 2015; 13(2):3996). We changed the Introduction and Methods sections to add this information:

*“The aim of this study was to evaluate the effectiveness of selected mitigation measures from the Dutch policy document ‘Healthy Growth, Sustainable Harvest’ on non-target terrestrial arthropod, focusing on spring-breeding carnivorous ground beetles (Carabidae) being important for biological control of insect pests.”* (lines 97-100)

*“We used the carabid beetle Bembidion lampros (Herbst, 1784) as a model species as it represents a group of spring-breeding, carnivorous ground beetles of temperate agricultural landscapes important for natural pest control [29,30] in agro-ecosystems. (…) It has been also indicated as relevant for risk assessment of pesticides according to the EFSA Scientific Opinion addressing the state of the science on risk assessment of plant protection products for non-target arthropods [34].”* (lines 123-133)

We agree that as the results and conclusions drawn in our study are based on the use of *Bembidion lampros* as an example, the actual numbers and precise mechanisms used to arrive at these predictions will not match other species with differing life cycles. However, the fact that the spatial dynamics of this species can result in the observed results is of general concern and allows to draw more general conclusions presented in our study.

According to our knowledge, *Bembidion lampros* is one of the key species in arable farmlands of the Netherlands, along with *Bembidion properans,* which have similar life history and habitat preferences (Turin and Alebeek 2007; Saska et al. 2007). In Turin et al. (2022) both of species are mentioned among the “25 most agriculture tolerant species, abundant in farmland and ruderal sites” (Fig. 5.18). Thank you for pointing us to this latest publication on beetles in the Netherlands. We added this reference to the text (see line 130).

As both Reviewers suggested that including more information on species biology and ecology would help to better evaluate and understand the analysis and results, this information has been now added to section 2.2. The section has been divided into two sub-sections: 2.2.1 describing the species biology and ecology and 2.2.2 explaining implementation and model procedures in ALMaSS.

2. *A second criticism is that the model was not validated, nor was a sensitivity analysis done on model parameters. Without such procedures, it is impossible to know how sensitive model parameters are to changing input. Likewise, a validation would provide confidence in the predictions of the model. If the authors have these data, they are encouraged to present them. My final comment concerns the discussion section, which nicely describes and contextualizes the results. However, important caveats of the study must be presented, including the fact that only a single, carnivorous carabid was used, model parameters were not tested for sensitivity, and no validation was done on model output.*

The model used in this study is well established and has been commonly accepted for regulatory purposes (see EFSA Scientific Opinion addressing the state of the science on risk assessment of plant protection products for non-target arthropods; EFSA Journal 2015; 13(2):3996). The model has been reviewed many times in scientific publications (list provided below) and it has never been suggested that the model parametrization is unrealistic. The model was validated, and the sensitivity analyses were conducted, please see original publications cited in the text (e.g., Bilde and Topping 2004).

List of scientific publications using the Bembidion ALMaSS model:

- Bilde T., Topping C.J. (2004) Life history traits interact with landscape composition to influence population dynamics of a terrestrial arthropod: A simulation study. Ecoscience 11 (1): 64-73.
- Jepsen J.U., Topping C.J., Odderskær P., Andersen P.N. (2005). Evaluating consequences of land-use strategies on wildlife populations using multiple-species predictive scenarios. Agriculture, Ecosystems & Environment 105 (4): 581-594.
- Sibly R.M., Nabe-Nielsen J., Forchhammer M.C., Forbes V.E., Topping C.J. (2009) The effects of spatial and temporal heterogeneity on the population dynamics of four animal species in a Danish landscape. BMC ecology 9, 18.
- Nabe-Nielsen J., Sibly R.M., Forchhammer M.C., Forbes V.E., Topping C.J. (2010). The Effects of Landscape Modifications on the Long-Term Persistence of Animal Populations. PlosOne 5 (1): e8932.
- Topping C.J., Lagisz M. (2012) Spatial Dynamic Factors Affecting Population-Level Risk Assessment for a Terrestrial Arthropod: An Agent-Based Modeling Approach. Human and Ecological Risk Assessment: An International Journal 18 (1): 168-180.
- Topping C.J., Kjær L.J., Hommen U., Høye T.T., Preuss T.G., Sibly R.M., van Vliet P. (2014) Recovery based on plot experiments is a poor predictor of landscape-level population impacts of agricultural pesticides. Environmental Toxicology and Chemistry 33 (7): 1499-1507.
- Aagaard A., et al. (2015) EFSA Scientific Opinion addressing the state of the science on risk assessment of plant protection products for non-target arthropods. EFSA Journal 13 (2): 3996.
- Topping C.J., et al. (2015) Towards a landscape scale management of pesticides: ERA using changes in modelled occupancy and abundance to assess long-term population impacts of pesticides. Science of The Total Environment 537: 159-169.
- Ziółkowska E., Topping C.J., Bednarska A.J., Laskowski R. (2021) Supporting non-target arthropods in agroecosystems: Modelling effects of insecticides and landscape structure on carabids in agricultural landscapes. Science of The Total Environment 774: 145746.

3. *Another critical caveat of the model is that it treats all crops equally when it comes to pesticide exposure but in reality, crop canopies differ markedly among crops. As such, pesticide penetration to the lower canopy and thus, the habitat of the ground beetle, will be different among crops. This detail is important, but not considered in the study.*

As the model uses crop management plans provided by farmer advisors, the timing and number of applications of pesticides is relevant to the specific crop. As indicated by the Reviewer, the crop canopies (crop interceptions) were not included when calculating the insecticides’ impacts, hence assuming the worst case estimate of exposure. However, including crop interception should not change the results substantially as, according to the crop management plans, the timing of application of insecticides is often when the crop canopy is already complete or close to complete.

*Specific comments:*

4. *At first mention of B. Lampros, mention that it is carnivorous. Clarify why omnivorous/granivorous carabid species were not included as the model species. Of particular importance as well is the fact B. Lampros is classified as a heathland species, and not one of particular importance in cropped fields (Turin et al. 2022). This contrasts with the objects of the model and makes it an odd choice for a model species.*

As already explained in response to comment (1), *Bembidion lampros* was used in this study as a representative species for spring-breeding, carnivorous ground beetles of temperate agricultural landscapes, being useful natural enemy of pests in agricultural fields, and relevant for risk assessment of pesticides. It is regarded as important aphid predator at the start of infestations, especially of cereals. In addition, according to our knowledge it is abundant species in agricultural landscapes in many European countries, including the Netherlands (e.g., in Turin et al. 2022 it was mentioned among the 25 most agriculture tolerant species, abundant in farmland and ruderal sites; see Fig. 5.18).

5. *L99 – provide nomenclature of species at first mention – in this case for B. Lampros, it is Herbst, 1784)*

Done.

6. *L133 – I believe manuscript would benefit from a schematic showing the model and its parameters, as well as the process and statistics used in the model*

Thank you for this suggestion but, as already mentioned in response to comment (1), the *Bembidion* model we used was published before together with detailed description of the model’s parametrization, procedures and statistics (see Bilde and Topping 2004, and ALMaSS ODdox documentation for the species: https://projects.au.dk/fileadmin/dmu.dk/en/animalsplants/almass/ALMaSS/Carabid_B/index.html).

7. *L185-187 – As part of this study?*

Yes, the hierarchical cluster analysis was part of this study. We rephrased the sentence to clarify this issue.

8. *L238 – Explain ‘real world beetles’*

It means “in the real world”, i.e., because the beetle densities are high in real landscapes, one agent in the model represents 100 beetles in the real world. Such procedure was necessary to make the simulation computationally feasible. We rephrased the sentence to make it more clear (lines 269-271).

9. *L240 – The assumption that males do not limit population size is erroneous. Populations cannot grow without males present, so how can a female only population be a realistic scenario upon which to build a model for policy decisions?*

We believe that the Reviewer misunderstood our intentions. We do not claim that the population can grow without males present. As the sex ratio in the *Bembidion* populations is close to 50:50, in the model we are assuming that all the females are being fertilized and can produce eggs. Therefore, it has no sense to track males in the model, hence they are not included. Including both females and males would make sense if the model considers energetic component and/or direct food intake, which is not the case for the *Bembidion* model. We changed the sentence as follows (lines 272-275):

*“All individuals are categorized as being members of four life-stages: egg, larvae, pupae, and adult female. We assume that all the adult females are being fertilized and can produce eggs, hence adult males are not being followed in the model.”*

10. *L270 – Please provide a citation to show there is no impact of fungicides on carabid beetles*

We do not claim that fungicides have no impacts on NTA’s. In our study we assumed that there is no direct impacts (acute toxicity), and we did not model indirect effects on beetles. We added this clarification to the text (see line 319).

11. *L290 – beetles were exposed to a pesticide for a period defined by at least 1-25 days. However, carabids are highly mobile and therefore would not be exposed to the same level of toxicity over time. They could move out of the field, move to an area where the canopy intercepted more of the pesticide, etc. I don’t understand how they can be modeled to have continuous exposure for 25 days.*

The Reviewer is completely right but the implementation of pesticide application is as described in the manuscript. There is an environmental degradation rate for insecticides which is applied. The beetles move through the landscapes and whenever they enter the contaminated field, the toxicity level is accumulated and when it exceeds the threshold they die.

As we explained in our response to comment (7) by Reviewer #1, such implementation matches the implementation presented in the EFSA Scientific Opinion addressing the state of the science on risk assessment of plant protection products for non-target arthropods (EFSA Journal 2015; 13(2):3996). It is even more elaborated than in the ‘traditional’ risk assessment of pesticides. It was shown that with this implementation it is possible to successfully investigate the potential of source–sink dynamics to indirectly affect untreated populations, the impact of long-term year-on year application of the pesticide and the effect of landscape structure on effects (see EFSA Scientific Opinion mentioned above).

12. *L327 – the authors do not define what is a replicate in this study*

A replicate in our study is one simulation run of a given combination of applied scenarios (mitigation measures). Each combination of applied scenarios (16 in total) was replicated 10 times, as explained in lines 380-383.

13. *L365 – Which R package was used here and how were the analyses conducted? Please provide more depth*

We used ‘stats’ and ‘MASS’ packages (Venables and Ripley 2002). This information together with the reference were added to the manuscript (line 420).

14. *L447 – I don’t tend to agree with this. As you state in the previous section, the results were mainly due to the large impact of pesticide toxicity and not the impact of field margin. It is likely that the effects of pesticide toxicity masked those of field margin, but this possibility seems not considered here.*

We removed the word ‘substantial’ from the sentence. In our study, we first analysed effects of decreasing toxicity of applied insecticides separately from effects of increasing the amount of field margins in a landscape, and this showed that effects of the first mitigation measure are larger than of the second one. However, if measures are applied together the effects are not simply additive but rather magnified.

15. *L461 – what is meant by “realistically simulate”? The model is based on assumptions that are unlikely to be true for other carabid species, and has provided limited justification for including B. lampros as the model species in this study. Clarification is needed here*

We do not agree with this comment. As already explained in our response to comment (1), *Bembidion lampros* was used in this study as a representative species for a group of spring-breeding, carnivorous ground beetles of temperate agricultural landscapes. The results and conclusions drawn in our study may be considered valid for the species with similar life history. The parameters in the model are based on field and laboratory data on *Bembidion lampros* from the literature. When necessary data were not available, the parameters were estimated based on published data from other ground beetles with a similar life history. The model has been reviewed many times in scientific publications (list provided above) and it has never been suggested that the model parametrization is unrealistic.

16. *L468 – Are these measures actually relevant at the EU level? Please provide evidence for this. Member countries currently have and continue to implement their own guidelines with full autonomy. Again, the results obtained here are based on a simulation with Dutch conditions.*

The sentence refers to the measures, not the results described. Measures tested in our study are used widely across the EU member countries, e.g. field margins are compulsory across the EU according to the water framework directive, and commonly applied as greening measures inside Common Agricultural Policy. Decreasing of spray drift is also commonly used, recommended or even mandatory in most countries; and reducing the use and risk of chemical pesticides by 50% by 2030 is one of the primary objectives of the European Green Deal.

17. *L485 – this paragraph nicely addresses the caveat for animal farms in mixed farming. It is prudent to also include a caveat that crop architecture was considered equivalent for all crops in the model, which may provide poor estimates of pesticide sensitivity to due interception by foliage. In addition, provide a caveat that beetles were assumed to be largely stationary and thus, the model makes not provision for immigration or emigration of beetles into a particular field or cell.*

The Reviewer misunderstood the study, as simulation of beetle movement is included in the model. The adult beetle starts life when it emerges from the pupa, most likely in the middle of a field. It then spends time moving and foraging until late summer when aggregation behaviour is triggered and it will move towards field margins and other beetles. When it finds a suitable overwintering site, or when winter arrives the beetle enters hibernation. In spring the beetles come out of hibernation and disperse away from field boundaries and other beetles. During this time they will also lay eggs as they move. Once all the eggs are laid, or if the beetle lives to aggregation, then the beetle dies. When moving, beetles may enter fields which were sprayed and then they affected by pesticide’s toxicity.

As we already explained in our response to comment (3), the canopy architecture (crop interception) was not considered when calculating the impact of pesticides, hence assuming the worst case estimate of exposure.

18. *L583 – this is not true. B. lampros is wing dimorphic and disperses by flight (Turin et al, 2022) and thus, the smaller landscape scale is not sufficient and at worst, potentially erroneous.*

According to our knowledge from the field studies, as well as from the literature, the *Bembidion lampros* is indeed wing dimorphic and polymorphic species but the majority of the individuals do not exhibit functional wings and/or functional flight musculature (see Hendrickx et al. 2009. *Pervasive effects of dispersal limitation on within- and among-community species richness in agricultural landscapes*. Global Ecology and Biogeography 18: 607-616). Development of functional wings and/or functional flight musculature is a genetic response to the poor environmental conditions. Due to these reasons we decided not to model dispersal by flight.

We added information on wing dimorphism in *Bembidion lampros* to the description of species biology and ecology in section 2.2 (lines 247-248).

19. *L585 – I don’t disagree with the conclusion, but it must be pointed out that the assumptions and biology of the model are not necessarily realistic. I realize this may preclude publication, but this can be handled by re-running the model based on more realistic biological parameters.*

We do not agree with this comment. As already mentioned in our previous responses, the parametrization of the model was based on field and laboratory data. The *Bembidion* model has been well accepted for regulatory purposes, has been reviewed many times in scientific publications and it has never been suggested that the model parametrization is unrealistic.

20. *L592 – most certainly Bembidion metrics should not be used to assess impacts on pollinators, the question is should the even be used to assess impacts on other carabids? This should be justified here.*

As we mentioned before, *Bembidion lampros* was used in this study as a representative species for a group of spring-breeding, carnivorous ground beetles of temperate agricultural landscapes. The results and conclusions drawn in our study may be considered valid for the species with similar life history. Turin et al. (2022) also lists Bembidion lampros as species often used in pesticide regulation, due to its sensitivity (chapter 7.3.5).

21. *Figure 4 – please show the SE on each bar, and include the names of the 10 study areas in the figure caption.*

Done.

22. *Figures 5-7 – It seems this data would be better represented in a series of heatmaps, where 1 cell of the heatmap would be used to show the results in each study area.*

We do not think that heatmaps are a good way of presenting our results. We used AOR graphs because it is accepted way of presenting complex responses of the model (see paper by Hoye et al. 2012. *Interpreting outputs of agent-based models using abundance–occupancy relationships*. Ecological Indicators 20: 221-227) while heatmaps are just presenting the static view at a given point of time.

23. *Several editorial comments appear in the attached document.*

Thank you for these comments. We included them in the revised version of the manuscript.
