# Supplementary material for: Is the effectiveness of policy-driven mitigation measures on carabid populations driven by landscape and farmland heterogeneity? Applying a modelling approach in the Dutch agroecosystems
Source: PLoS One. 2022 Dec 27;17(12):e0279639. doi: 10.1371/journal.pone.0279639 (PMC9794068; doi:10.1371/journal.pone.0279639)
Supplement: Attachment — Submitted filename: Response_to_Reviewers.docx [file pone.0279639.s009.docx]

**Response to Reviewers and Editor**

We thank Referees and Editor for the helpful comments. Below we provide point-by-point answers to the points raised by the Reviewer #1 and Reviewer #2. All the line numbers refer to the revised manuscript with tracked changes.

**REVIEWER #1:**

1. *I see from other comments that the question of sensitivity analysis was brought up - and even though I can see the point of the authors that this model has been thoroughly analyzed elsewhere, I think it would be good to hear from the authors what they think how the assumptions about the biology, especially movement, influence the simulation results. There should be a section in the discussion that addresses this, perhaps including the main findings from previous analyses, as we don't expect readers to dive in all the previous publications, but would still like to be informed about the analyses relevant for the simulations presented.*

Thank you for this comment. We agree with the Reviewer that summarizing shortly the sensitivity of the *Bembidion* model to some of the crucial parameters related to the species biology (such as dispersal) would help the readers to better understand the model itself and would show the results of our study in a broader perspective. We have therefore modified the Discussion section by adding the subsection 4.3 ‘Impacts of model beetle dispersal abilities on simulations’ (lines 597-614).

**REVIEWER #2:**

1. *While the authors provide more evidence for the use of their chosen species, their conclusions are wide-ranging and do not reflect the fact only a single beetle species was used. Should policy be based on a model tested with only a single species? Moreover, L30-31 suggest results were highly context-specific, which they are likely to be carabid species as well. Yes, they have used a species that is in the top 25 most trapped in ag fields, but species #25, if it was, is a substantial difference from the top 10 species. L32-34 suggests this model as a powerful tool to support policy, but how can we conclude this is so when only a single carabid species was used? Until the model can be proved to be non taxon-specific, (it is currently contextually-specific), I remain on the sidelines as to the validity of its predictions and use in policy development. I believe the authors can accommodate this is the markedly tone down the conclusions and add the caveats suggested by the reviewers.*

Thank you for this comment and for sharing your doubts regarding selection of the species for our modelling study. We agree that the policy shouldn’t be driven by results for a single species, and we do not claim this in our study. We rather say that “*the use of simulation to assess complex interactions between landscape, ecology and behaviour of species, and policy measures can be a powerful tool supporting innovative policy management*” (lines 32-34). This means that the methods we presented in our study provide an integrated framework for simulation of landscapes, farming and physical chemical environment and allow to better understand complex interactions between landscape, ecology and behaviour of species, and therefore can support policy.

This was, in fact, the case of our study as the results supported the claims presented in the interim assessment of the policy document ‘Healthy Growth, Sustainable Harvest’ (please see Tiktak A, Bleeker A, Boezemanm van DJ, et al. *A closer look at integrated pest management. Interim assessment of the policy document ‘Healthy Growth, Sustainable Harvest’*. English summary of the Dutch report *“Geïntegreerde gewasbescherming nader beschouwd. Tussenevaluatie van de nota Gezonde Groei, Duurzame Oogst”*. PBL Netherlands Environmental Assessment Agency; 2019). Besides results described in our study, the report also used modelling results for other focal species (hare and skylark) which our team conducted, as well as many others.

Our study focuses on *Bembidion lampros* being a focal species for Coleoptera used in environmental risk assessment. We do not claim that our findings and conclusions can be easily transferred to other focal species, and we emphasize that in the conclusions: “*Due to the degree of context specificity, once defined these metrics should not be taken out of the context of the key ecosystem service provider they are targeted towards. For example, Bembidion metrics should not be used to assess likely impacts of policy on pollinators.*” (lines 663 – 666). Regarding the reasons for selection of this specific focal species for our study, we believe that we presented them in details in our response to previous comments of the Reviewer.

2. *In addition, the model needs a sensitivity analysis done when it is reparameterized, was done here. I understand this was done upon completion of the model in previous work, but readers cannot have confidence in the model unless it is shown just how sensitive the parameters are to the starting values of model runs. This is especially true for the canopy effects (pesticide interception), where data were not tested and were not included from field scenarios. If this parameter is overly sensitive, it needs data and assumptions are not enough to produce accurate model results. I would more happily accept their argument on this parameter if I could be assured it was not that sensitive and as such, their absence of data on the subject would have little impact on the overall model predictions.*

Thank you for this comment. We would like to emphasize that the model for *Bembidion lampros* used in this study has not been re-parametrized since its original publication. The environment (landscape) in which the individual beetles (agents) are placed in is, of course, different and the steps of landscape generation process for the Netherlands are described in details in the Appendix A. The parameters related to impacts of pesticide applications on beetles (such as the application rate or the rate of degradation of pesticides) are part of the scenario definition and are described in section 2.3.

The decreasing probability of field mortality simulates substitution of harmful insecticides by less harmful insecticides but can be considered equivalent to a reduction in exposure or application rate, or toxicological impact of insecticides in the field. It can therefore also simulate the impacts of canopy cover in the sense that if canopy is more dense then less insecticides reach the beetles moving on the ground meaning that the probability of field mortality will decrease. We have now added this explanation in section 2.3.1 (lines 315-320).

3. *I appreciate the citation of the Bilde and Topping (2004) publication but I think this manuscript would benefit from even brief description of the model schematic and its parameters. Readers should not have to go to another publication to understand the model being used here, and this can be easily added in a supplemental file.*

Thank you for this comment. We believe than the re-written section 2.2 of the Methods (after changes we made in response to the previous Reviewers’ comments) provides a good overview of the *Bembidion* model. The readers who are interested in more details are re-directed to ALMaSS Bembidion ODdox documentation (see lines 262 – 265) and the previous publications. Although if the Reviewer and the Editor believe that more details are necessary, we can provide then ALMaSS Bembidion ODdox as an appendix to this manuscript (but it is the same as the published one).

4. *The author response to my initial comments on point #15, regarding realistic simulations, is mostly valid. I do agree that their simulations apply to all beetles with similar life history, but the agro ecosystem is rife with species that have a similar life history to the species used here but that may feed at different trophic levels and as such, the impacts of the model may be different for the agroecosystem as a whole based on model predictions. For example, would omnivorous or granivorous respond similar to model predictions, where these species have similar life histories? Again, it may be useful to couch the conclusions in this regard, and limit them to the species studied and point out conclusions may vary depending on species used.*

Thank you for this comment. As we already mentioned, the *Bembidion lampros* was chosen for this study as it is one of the focal species for environmental risk assessment of arthropods. It is important for natural pest control, and widespread in the agricultural landscapes in central and northern Europe, including the Netherlands. Of course you are right that beetles with similar life history may feed at different trophic levels. This may indeed impact model predictions but only if the model considers energetic component and/or direct food intake, which is not the case for the *Bembidion* model we are presenting in this study. In our model we assume that the beetles always get enough food, which we think is a reasonable approach due to rather broad food preferences of *Bembidion lampros* (it feeds on Collembola, Diptera, mites, earthworms, small beetles and eggs from different vermin flies, for example frit fly eggs, *Ocinella frit* L.) and its abilities to survive long periods of starving (our experience from laboratory studies).
